# Isolation and Comparative Transcriptome Analysis of Human Fetal and iPSC-Derived Cone Photoreceptor Cells

**DOI:** 10.1016/j.stemcr.2017.10.018

**Published:** 2017-11-16

**Authors:** Emily Welby, Jorn Lakowski, Valentina Di Foggia, Dimitri Budinger, Anai Gonzalez-Cordero, Aaron T.L. Lun, Michael Epstein, Aara Patel, Elisa Cuevas, Kamil Kruczek, Arifa Naeem, Federico Minneci, Mike Hubank, David T. Jones, John C. Marioni, Robin R. Ali, Jane C. Sowden

**Affiliations:** 1 Stem Cells and Regenerative Medicine Section, UCL Great Ormond Street Institute of Child Health, 30 Guilford Street, London WC1N 1EH, UK; 2 NIHR Great Ormond Street Hospital Biomedical Research Centre, UCL Great Ormond Street Institute of Child Health, 30 Guilford Street, London WC1N 1EH, UK; 3 Department of Genetics, UCL Institute of Ophthalmology, 11-43 Bath Street, London EC1V 9EL, UK; 4 Cancer Research UK Cambridge Institute, University of Cambridge, Li Ka Shing Centre, Robinson Way, Cambridge CB2 0RE, UK; 5 Department of Computer Science, University College London, Gower Street, London WC1E 6BT, UK; 6 UCL Genomics, UCL Great Ormond Street Institute of Child Health, 30 Guilford Street, London WC1N 1EH, UK; 7 EMBL-European Bioinformatics Institute, Wellcome Genome Campus, Hinxton, Cambridge CB10 1SD, UK

**Keywords:** retinal dystrophies, cone photoreceptor cells, transcriptome, human pluripotent stem cells, retinal organoids, cell surface markers, cell transplantation therapy

## Abstract

Loss of cone photoreceptors, crucial for daylight vision, has the greatest impact on sight in retinal degeneration. Transplantation of stem cell-derived L/M-opsin cones, which form 90% of the human cone population, could provide a feasible therapy to restore vision. However, transcriptomic similarities between fetal and stem cell-derived cones remain to be defined, in addition to development of cone cell purification strategies. Here, we report an analysis of the human L/M-opsin cone photoreceptor transcriptome using an AAV2/9.pR2.1:GFP reporter. This led to the identification of a cone-enriched gene signature, which we used to demonstrate similar gene expression between fetal and stem cell-derived cones. We then defined a cluster of differentiation marker combination that, when used for cell sorting, significantly enriches for cone photoreceptors from the fetal retina and stem cell-derived retinal organoids, respectively. These data may facilitate more efficient isolation of human stem cell-derived cones for use in clinical transplantation studies.

## Highlights

• Definition of an L/M-opsin cone-enriched gene signature from human fetal cones
• Single-cell analysis revealed heterogeneity based on cone maturation stage
• Fetal and stem cell-derived cones show similar expression of cone gene signature
• FACS with SSEA1/CD133/CD26/CD147 markers enriches for human cones

## Graphical Abstract

**Figure:**
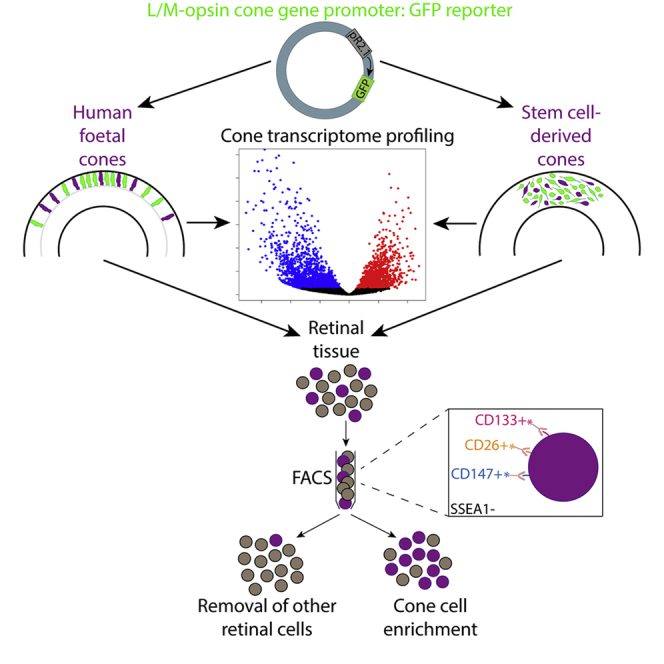

## Teaser

Welby et al. define a cone-enriched gene signature within a human fetal L/M-opsin cone population, which is used as a baseline reference to demonstrate similar cone gene expression between *bona fide* and stem cell-derived L/M-opsin cone cells. Furthermore, profiling of cell surface molecules in human fetal cones led to the generation of a cluster of differentiation marker panel, which provides enrichment of fetal and stem cell-derived cones.

## Introduction

Retinopathies featuring the progressive degeneration of the rod and cone photoreceptor cells lead to permanent blindness. Inherited retinal dystrophies affect 1 in 3,000 people worldwide (Bessant et al., 2001), with the most common form, retinitis pigmentosa, presenting with primary rod degeneration followed by the loss of cones. Age-related macular degeneration results in the primary loss of cones in the macula. Cone degeneration has the greatest impact on sight since cones are fundamental for the detection of color, daylight vision, and high visual acuity. The human retina contains three different types of cone photoreceptors; approximately 5%–10% of the cone population express S-opsin, while the majority of cones (90%–95%) express either L-opsin or M-opsin light-sensitive proteins (Craft et al., 2014). Even though cone photoreceptors are a rare population, forming 2%–4% of total retinal cells, humans are dependent on these cells for optimal vision.

There is a paucity of effective treatments for retinal degeneration. Cell-replacement therapy offers an approach for advanced disease with extensive cone photoreceptor loss. As inner retinal neurons are initially preserved within the diseased retina, photoreceptor cell replacement might provide a feasible approach following proof-of-concept studies in the mouse (MacLaren et al., 2006, Bartsch et al., 2008, Santos-Ferreira et al., 2016b), which showed improvements in visual function following transplantation (Pearson et al., 2012, Lamba et al., 2009). However, recent studies suggest that functional recovery may be achieved by transplanted photoreceptor cells predominantly transferring genetic material, rather than making new connections with the host retina (Pearson et al., 2016, Santos-Ferreira et al., 2016a, Decembrini et al., 2017), and thus developing ways to improve functional connectivity of transplanted cells remains a priority. Combining induced pluripotent stem cell (iPSC) generation (Takahashi et al., 2007) and retinal organoid culture systems (Meyer et al., 2009, Nakano et al., 2012, Zhong et al., 2014, Reichman et al., 2014) has allowed recapitulation of human retinogenesis and the generation of photoreceptors from human somatic cell sources which could provide a renewable source of autologous cells for cone transplantation therapy. Nevertheless, there has yet to be a direct comparative study of the transcriptome of human fetal and PSC-derived cone photoreceptors to assess their degree of similarity.

In addition to providing baseline data for assessing suitability of iPSC-derived cones for therapeutic applications, establishing the transcriptome of human developing L/M-opsin cones would allow exploration of the molecular determinants needed for early specification and maintenance of the cone cell fate, which remains to be fully elucidated. In the human retina, from as early as fetal week 10.9, cones and rods can be distinguished based on cell morphology, nuclei condensation, and proximity to the central retina where the fovea forms (Xiao and Hendrickson, 2000, Hendrickson et al., 2008). To date, the most well-documented human cone-specific markers are the cone opsins (Xiao and Hendrickson, 2000, Cornish et al., 2004), with S-cones appearing first, followed by L/M-cones. L- and M-opsin-expressing photoreceptors can be distinguished via high-resolution retinal imaging and retinal densitometry (Roorda and Williams, 1999, Hofer et al., 2005), but cannot be separated via immunohistochemistry due to the high homology (98%) of the *OPN1LW* and *OPN1MW* genes (Nathans et al., 1986, Cornish et al., 2004). Several studies in the mouse, suggest that rod and cone photoreceptors share a common cell lineage and define S-cones as a default cell fate pathway, which is driven through the interaction of the cone-rod homeobox gene (*Crx*) and the nuclear receptor RAR-related orphan receptors (*Rora/Rorb*) (Fujieda et al., 2009, Srinivas et al., 2006, Kim et al., 2016). To become M-cones, precursor cells must express transcription factor genes, including retinoid-x-receptor gamma (*Rxrg*) and thyroid hormone receptor beta 2 (*Thrb2*), which regulate cone cell identity (Ng et al., 2001, Roberts et al., 2005). Other lines of evidence define the Onecut homeobox 1 (*ONECUT1*) and spalt-like transcription factor (*Sall3*) genes, as necessary for generating progenitors biased to produce cone and horizontal cells (Emerson et al., 2013, de Melo et al., 2011, Suzuki et al., 2013). Moreover, in the human retina the characterization of cone-associated regulatory factors remains to be explored. Cone transcriptome data only exist for adult mouse cone photoreceptors (Siegert et al., 2009); in human, only total retina (Hornan et al., 2007, Cai et al., 2012, Bowes Rickman et al., 2006, Whitmore et al., 2014, Li et al., 2014) and isolated Crx.GFP-expressing embryonic stem cell (ESC)-derived cells (Kaewkhaw et al., 2015) have been analyzed. An equivalent dataset for human fetal cone photoreceptors is currently lacking. Furthermore, single-cell RNA sequencing (RNA-seq) (Lun et al., 2016) offers a novel approach to explore the heterogeneity of human cone cell populations.

The enrichment of cone photoreceptors without genetic manipulation (e.g., GFP reporter gene labeling) will be crucial prior to transplantation into human patients. We, and others, have previously described the enrichment of mouse rod photoreceptor precursor cells by targeting antigens expressed on the outer cell membrane, with corresponding antibodies conjugated to fluorophores in cell-sorting strategies (Eberle et al., 2011, Eberle et al., 2014, Lakowski et al., 2011, Eberle et al., 2014). An extended cell surface biomarker panel was developed to enrich rod photoreceptors from mouse ESC-derived retinal cultures prior to transplantation (Lakowski et al., 2015). However, little progress has been made toward defining cell surface markers profiles or enrichment approaches for cone photoreceptors.

In this study, we used an adeno-associated virus (AAV) GFP reporter, driven by a human L/M-opsin gene promoter (pR2.1:GFP), to specifically label and isolate human fetal cones and an equivalent population from iPSC-derived retinal differentiation cultures. We report transcriptome analysis of developing L/M-opsin cone photoreceptor populations and single cells, identifying genes that are upregulated in early and late L/M-opsin cones. A direct comparative analysis of fetal and iPSC-derived cells demonstrated similar cone gene expression. From these transcriptome data and using fluorescence-activated cell-sorting (FACS) analysis, we established the cell surface marker expression profile of L/M-opsin cone photoreceptors, which led to the identification a cone biomarker panel (SSEA1−CD26+CD133+CD147+) that positively enriches for fetal L/M-opsin cones and a stem cell-derived cone photoreceptor population.

## Results

### Cone Photoreceptor Marker Expression in the Developing Human Fetal Retina

**Figure 1:**
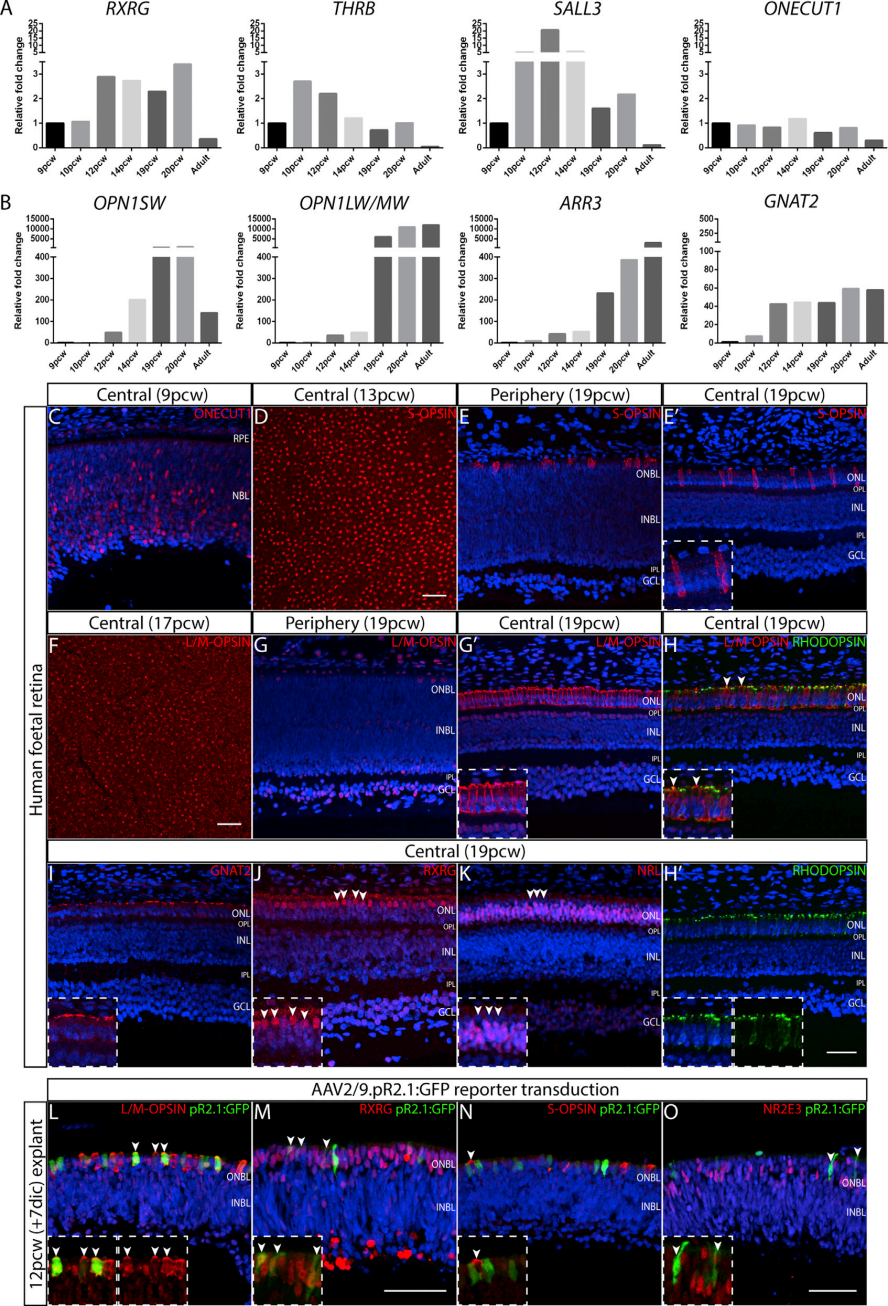
Analysis of Cone Photoreceptor Markers in the Developing Human Retina qRT-PCR analysis of early cone photoreceptor genes, *RXRG*, *THRB*, *SALL3*, and *ONECUT1* (A), and mature cone photoreceptor genes *OPN1SW*, *OPN1LW/MW*, *ARR3*, and *GNAT2* (B), in fetal (9–20 pcw) and adult retinal samples (n = 1 per time point). Immunohistochemistry analysis of cone photoreceptor markers, ONECUT1 (C), S-OPSIN (D–E′), L/M-OPSIN (F–H), GNAT2 (I), and RXRG (J), and rod photoreceptor markers, NRL (K) and RHODOPSIN (H and H′), within 9–19 pcw fetal retinae. Analysis was performed on retinal tissue sections (C, E, E′, and G–H’). Scale bar, 50 μm. Whole-mount retina (D and F). Scale bar, 100 μm. White arrowheads in (H) highlight mutually exclusive expression of L/M-OPSIN and RHODOPSIN and in (J and K) indicate different location of RXRG- and NRL-expressing cells. The AAV2/9 pR2.1:GFP reporter labels L/M-opsin cones of 12 pcw (+7 days *in vitro*) fetal retinal explant (L) (arrowheads). GFP+ cells also co-label with the early cone marker RXRG (M) (arrowheads) but do not co-localize with S-OPSIN (N) (arrowheads) or NR2E3 (O) (arrowheads). RPE, retinal pigment epithelium; NBL, neuroblastic layer; ONBL, outer neuroblastic layer; INBL, inner neuroblastic layer; ONL, outer nuclear layer; OPL, outer plexiform layer; INL, inner nuclear layer; IPL, inner plexiform layer; GCL, ganglion cell layer (see Figures S1–S3).

We first assessed the developmental expression profile of a set of cone photoreceptor markers in a series of fetal (9–20 post conception weeks [pcw]) and adult retinal samples by qRT-PCR (Figure 1). Transcription factor genes implicated in early cone photoreceptor development, *RXRG*, *THRB*, *SALL3*, and *ONECUT1*, showed varied expression levels during retinal development relative to 9 pcw and all were downregulated in the adult retina (Figure 1A). By contrast, markers of mature cone photoreceptors, the cone opsins (*OPN1SW* and *OPN1LW/MW*) and cone arrestin (*ARR3*) showed substantial upregulation in late fetal retinal samples (19 and 20 pcw; Figure 1B) and in the mature adult retina, with *OPN1LW/MW* expression showing the greatest fold change (11,000-fold) during development. Cone transducin (*GNAT2*) and the pan-photoreceptor markers, cone-rod homeobox gene (*CRX*) and recoverin (*RCRVN*), showed modest upregulation during development (Figures 1B and S1A).

Immunohistochemical analysis of cone, pan, and rod photoreceptor markers in fetal retinal tissue highlighted differences in retinal maturity and photoreceptor cell density in the central compared with the peripheral retina, with later detection of protein compared with transcript detection (Figures 1C–1K and S1). CRX and RECOVERIN protein were detected in the 9–19 pcw retina, with a greater density of highly expressing cells in the central retinal region compared with the periphery (Figure S1B). Conversely, proliferation marker KI67 was detected in the peripheral, but not central, retinal cells by 19 pcw (Figure S1C). Of the cone markers, only ONECUT1 protein was detected by 9 pcw in cells across the neuroblastic layer (Figure 1C). S-OPSIN protein was detected from 13 pcw onward (Figures 1D–1E′), followed by L/M-OPSIN from 17 pcw, each appearing first only in the central retina (Figures 1F–1H). By 19 pcw, L/M-OPSIN- and S-OPSIN-labeled cone photoreceptors of the central retina showed a distinct morphology compared with the peripheral regions (Figures 1E, 1E′, 1G, and 1G′). L/M-OPSIN and rod marker RHODOPSIN showed mutually exclusive immunostaining (Figures 1H and 1H′). GNAT2 localized to the apical region of the outer nuclear layer (ONL) in cone developing outer segments (Figure 1I); RXRG localized to the outermost cone cell bodies of the central ONL (Figure 1J), whereas rod markers, NRL and NR2E3, showed distinct immunostaining of developing rod cell bodies (Figures 1K and S1D).

### Transcriptome Analysis of Human Fetal L/M-Opsin Cone Photoreceptors

Having defined the progressive appearance of L/M-OPSIN-expressing cones in the human fetal retina and the paucity of early human cone markers, we sought to isolate and characterize the transcriptome of these cells using the AAV2/9.pR2.1:GFP reporter. The pR2.1 promoter contains the locus control region and enhancer regions found 5′ upstream of L- and M-opsin genes (*OPN1LW, OPN1MW*, and *OPN1MW2*) on the X chromosome (Nathans et al., 1989, Wang et al., 1992). It drives expression in canine and rat L/M-opsin cone photoreceptors (Komaromy et al., 2008, Li et al., 2008), but also within mouse M- and S-cones (Wang et al., 1992, Fei and Hughes, 2001). To test the specificity of the reporter within the human retina, we developed a free-floating explant culture system that supports the viability and integrity of fetal retina during the 7 day period required to achieve AAV vector expression (Figures S2A–S2C). GFP+ cells were observed 3–4 days *in vitro* after delivery of the reporter to early (12 pcw) and late (19 pcw) human fetal retinal explants (Figures S2D and S2E); cryosections showed GFP+ cells in the ONL, co-labeling with L/M-OPSIN protein (Figure 1L). GFP+ cells were also positive for early cone-specific marker, RXRG (Figure 1M), but negative for S-OPSIN (S-cone marker; Figure 1N), NR2E3 (rod marker; Figure 1O) and proliferation marker, KI67 (Figure S2F), indicating the specificity of the reporter virus to post-mitotic L/M-opsin cone cells (Figure S2G). The reporter also effectively labeled L/M-opsin cones in fetal retinae (14% of 14 pcw and 4% of 18 pcw cones labeled; Figures S3A and S3B).

**Figure 2:**
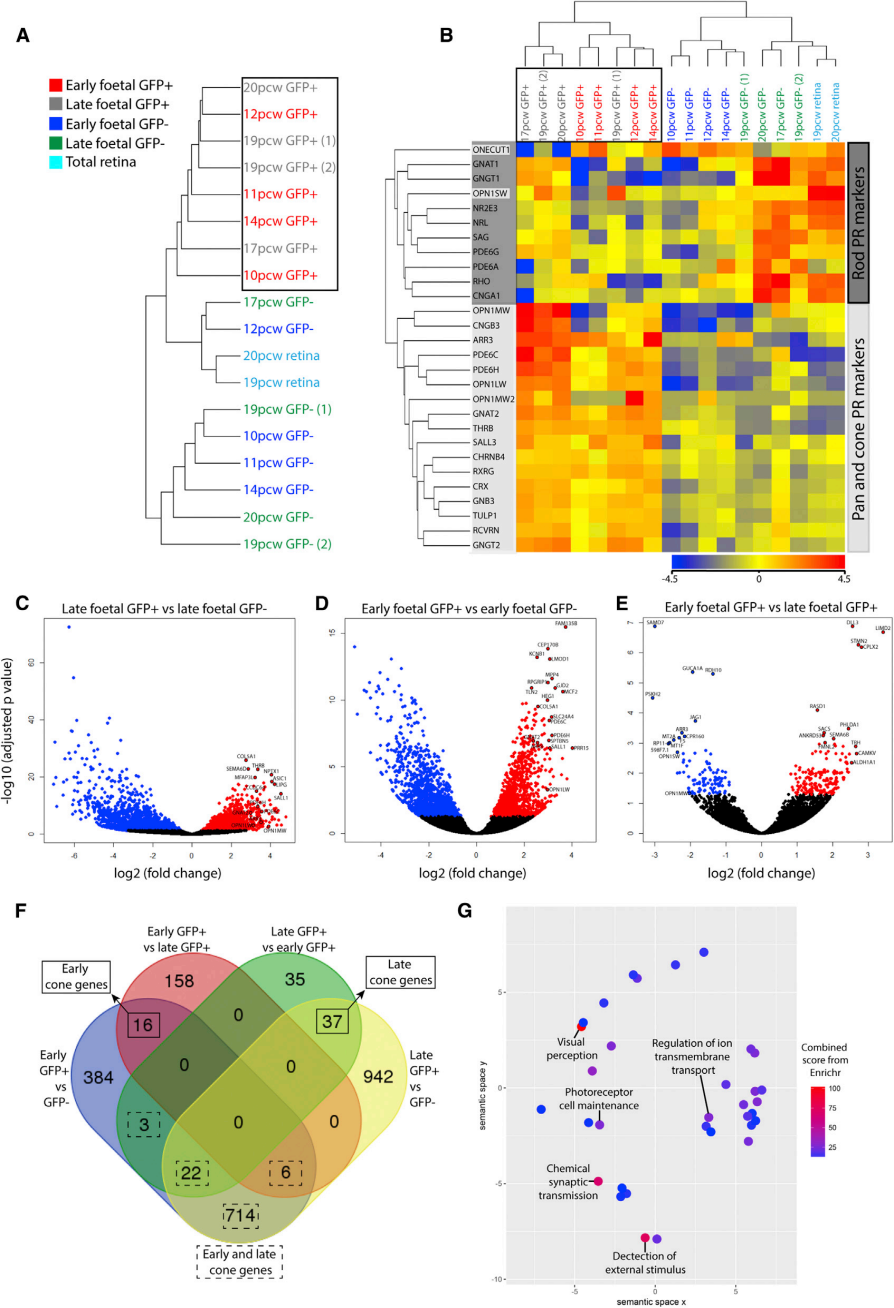
Transcriptome Analysis of Human Fetal AAV2/9 pR2.1:GFP-Labeled Cells (A–E) All fetal AAV2/9 pR2.1:GFP+ samples cluster together based on total transcript expression (A) (black box) and high expression of cone and pan-photoreceptor (PR) genes (B) (light gray box). Lower levels of rod-associated genes are detected in pR2.1:GFP+ samples (B) (dark gray box; *ONECUT1* and *OPN1SW* also group with the rod genes). Volcano plots representing the differential gene expression between late GFP+ and GFP− samples (C), early GFP+ and GFP− samples (D) and early GFP+ and late GFP+ samples (E). Significantly upregulated and downregulated genes (adjusted p value < 0.05) are highlighted in red and blue. (F) Venn diagram represents the overlap between significantly upregulated genes identified for the fetal GFP+ samples, revealing the 798 cone-enriched gene signature. (G) Revigo semantic plot demonstrates the enriched biological process GO terms associated with the cone gene signature. Color represents the combined score from Enrichr (see Figures S2 and S3; Table S1).

Early (n = 4) and late (n = 4) fetal retinal samples labeled with the AAV2/9 pR2.1:GFP reporter were treated by FACS (Figure S3C) to isolate the GFP+ and GFP− cells for RNA-seq, enabling the identification of highly expressed and enriched genes of the human L/M-opsin cone cell transcriptome. Unbiased hierarchical clustering analysis based on normalized gene expression showed that all GFP+ samples cluster together (Figure 2A, black box). Similarly, hierarchical clustering analysis based on the expression of selected established markers of cone, pan, and rod photoreceptors (n = 28) revealed all GFP+ samples cluster together (Figure 2B, black box), based on their high expression of cone- and pan-associated genes (Figure 2B, light gray genes). By contrast, the late GFP− and total retinal samples showed a higher expression of rod genes, particularly at later time points (Figure 2B, dark gray genes). Noteworthy, was the expression of *OPN1SW* in some of the late GFP+ samples. Together, these data support a cone identity for the isolated GFP+ cells from human fetal retinal explants labeled by the AAV2/9.pR2.1:GFP reporter.

Differential gene expression analyses were performed to identify genes highly enriched in early and late fetal GFP+ cones and genes differentially expressed between early and late cone populations (adjusted p value < 0.05). We identified 1,721 and 1,145 genes that were significantly upregulated in the late and early GFP+ cone populations, respectively, compared with the GFP− samples (Figures 2C and 2D, red data points; Table S1). Comparison of early versus late GFP+ expression profiles identified 180 and 96 genes upregulated in the early and late GFP+ cones, respectively (Figure 2E, red and blue data points, respectively; Table S1). Significantly upregulated genes with high fold changes are labeled on volcano plots and highlighted in Table S1. Merging these datasets identified 745 genes that are enriched in both early and late cone populations, in addition to 16 and 37 genes that are enriched in the early or late human cone populations, respectively (Figure 2F; Table S2). This set of 798 genes defines a unique cone-enriched gene signature, and includes genes with high fold enrichment that have established roles in cone and pan-photoreceptor functioning, developmental processes, synaptic processing and neurotransmission, extracellular matrix (ECM), and cell adhesion, in addition to genes with currently undefined function (Tables 1 and S2).

**Table 1:** Notable Genes from Cone-Enriched Gene Signature with High Fold Changes

Cone Population	Function	Genes	Fold Change
Upregulated in early and late cones	known cone photoreceptor associated genes	*OPN1MW*	15
		*PDE6C*	11.7
		*GUCA1A*	12.8
		*PDE6H*	10.3
		*ARR3*	12.1
		*RXRG*	5
		*THRB*	10.1
		*GNAT2*	7.9
		*CNGB3*	18.5
		*PDE6H*	10.3
	known photoreceptor associated genes	*MPP4*	16
		*RP1L1*	8.9
		*CACNA2D4*	10.6
		*LRIT3*	11.1
		*SLC12A5*	10.1
		*SAMD7*	5.8
		*SLITRK6*	6.2
		*CC2D2A*	7.8
		*RGS11*	18.6
		*RPGRIP1*	12.6
		*EGFLAM*	10.1
		*RD3*	9.1
	development	*SALL1*	23.3
		*PRR15*	16.1
		*GREM2*	9.1
		*KREMEN2*	11
		*HEG1*	9.8
		*LIPG*	18.5
		*BMP7*	7.1
		*THBS4*	8.5
		*SLC39A5*	18.1
	synaptic processing/neurotransmission	*NPTX1*	16.7
		*KCNB2*	13.8
		*KCNB1*	5.8
		*ASIC1*	16.7
		*KCNG2*	9.6
	non-coding RNA genes	*CTD-2521M24.11*	27.5
		*CTD-2034I21.1*	21.5
		*CTD-2050N2.1*	12.4
		*TNK2-AS1*	9.8
Upregulated in early cones	neural function	*DDN*	6
	negative regulator of Wnt signaling	*AMER2*	2.7
	nervous system development	*SEMA6B*	3.4
	synaptic processing	*CPLX2*	2.5
Upregulated in late cones	ECM/cell adhesion	*TNC*	3.2
		*PCDHAC1*	8.5
		*MATN2*	7.2
	photoreceptor outer segment	*RGS9BP*	5.6
	phototransduction	*RGS9*	5.1
	cilia function	*RSPH4A*	4
	development	*GSC2*	19.9
	interkinetic nuclear migration	*CEP120*	3
	cell differentiation and proliferation	*ST3GAL5*	2.7

Function and fold changes are provided of significantly upregulated genes with high fold changes that feature within cone-enriched gene signature. The full cone-enriched gene signature is provided in Table S2.

Gene ontology (GO) analysis on the 798 gene cone signature identified a high enrichment of biological processing GO terms for visual perception, detection of external stimulus, chemical synaptic transmission, photoreceptor cell maintenance, and regulation of ion transmembrane transport (Figure 2G; Table S3). GO terms containing the known cone regulatory genes, *RXRG*, *THRB*, and *RORA*, identified other genes associated with the “steroid hormone-mediated signaling pathway,” including *BMP7* and *NR2F6* (Table S3, genes in bold), which are without known function in cones.

### Single-Cell Transcriptome Analysis of Human Fetal L/M-Opsin Cone Photoreceptors

**Figure 3:**
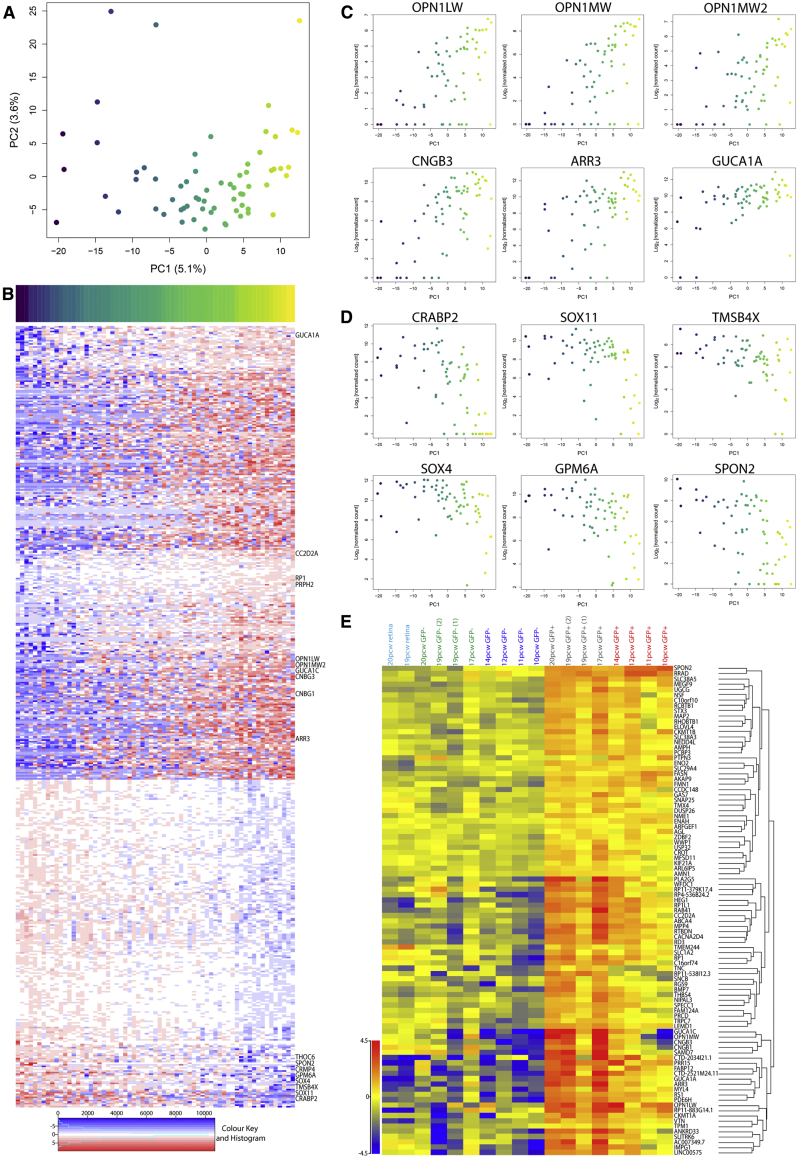
Single-Cell Transcriptome Analysis of AAV2/9 pR2.1:GFP+ Cells (A) Principal component analysis (PCA) plot of 65 individual AAV2/9 pR2.1:GFP+ cells from 15 pcw fetal retina, where each cell is colored based on its PC1 location (blue to yellow). The proportion of variance explained by the first two principal components is shown in brackets. (B) Heatmap of genes that are significantly differentially expressed (false discovery rate < 0.05) with respect to PC1. Columns represent single cells, which are ordered from left to right by their location on PC1. Each row represents a gene that is upregulated (blue to red; first block) or downregulated (red to blue; second block) along PC1. (C and D) Scatterplots showing gene expression (log2 normalized counts) profiles of key significantly differentially expressed genes which show upregulation (C) or downregulation (D) along PC1. (E) Heatmap displaying expression profiles of the 93 differentially expressed genes identified from single-cell RNA-seq analysis which feature within the cone-enriched gene signature (see Figure S4; Tables S2 and S4).

We next performed single-cell RNA-seq on the AAV2/9. pR2.1:GFP reporter-labeled population to explore the degree of cell heterogeneity at a single time point. The bulk RNA-seq analysis indicated the labeling of cells expressing *OPN1LW*, *OPN1MW*, *OPN1MW2*, and *OPN1SW* genes. Principal component analysis was performed to determine any systematic substructure within the pR2.1:GFP+ cell population, which revealed a continuum of cells with modest variability based on principal component 1 (PC1) (Figure 3A). Differential gene expression analysis correlated to PC1 revealed 503 significantly differentially expressed genes (false discovery rate < 0.05 Table S4), which showed an upregulation or downregulation as cells progress through PC1 (Figure 3B). We found that the expression profiles of genes that were significantly differentially expressed along PC1 correspond to increasing maturation across this population. Within the upregulated gene group, we noted a number of established genes associated with photoreceptor maturation including outer segment disc morphogenesis (*RP1* and *PRPH2*), primary cilium development (*CC2D2A*), phototransduction proteins (*CNBG3*, *CNGB1*, *GUCA1A*, *GUCA1C*, and *ARR3*), in addition to the L- and M-opsin genes (*OPN1LW* and *OPN1MW2*) (Figures 3B and 3C). In contrast, a number of genes that showed an overall downregulation across PC1 were associated with developmental processes, including transcription factors, *SOX4* and *SOX11*, retinoic acid binding protein, *CRABP2*, neurodevelopment disease gene, *THOC6*, cell migratory and neural differentiation-related genes, *TMSB4X*, *GPM6A*, *CRMP4,* and neural expressed ECM gene, *SPON2* (Figures 3B and 3D). Established pan (*RCVRN* and *CRX*) and cone photoreceptor genes (*RXRG* and *THRB*) did not significantly differ across PC1 (Figures S4A and S4B), which is consistent with their cone cell identity. S-cone (*OPN1SW*) and rod photoreceptor (*NRL* and *NR2E3*) gene expression, required for other photoreceptor cell fates, was detected in a small number of individual cells (Figure S4C).

We next examined whether the continuum of naive and mature cell states identified at a single time point represent true developmental trajectories. We found that 18.5% (93/503) of the differentially expressed genes identified in the single-cell analysis showed changed expression across developmental time when considering the early and late bulk mRNA transcriptome data (Figure 3E), including a large number of genes not previously reported in cone cells (Table S2, green highlighted genes). Hence, these data are consistent with the proposal that, at a single point in developmental time, individual cone cells exist in the retina at different stages of their developmental trajectory as revealed by their gene expression profiles.

### Comparative Transcriptomic Analysis with Human iPSC-Derived Cone Photoreceptors

**Figure 4:**
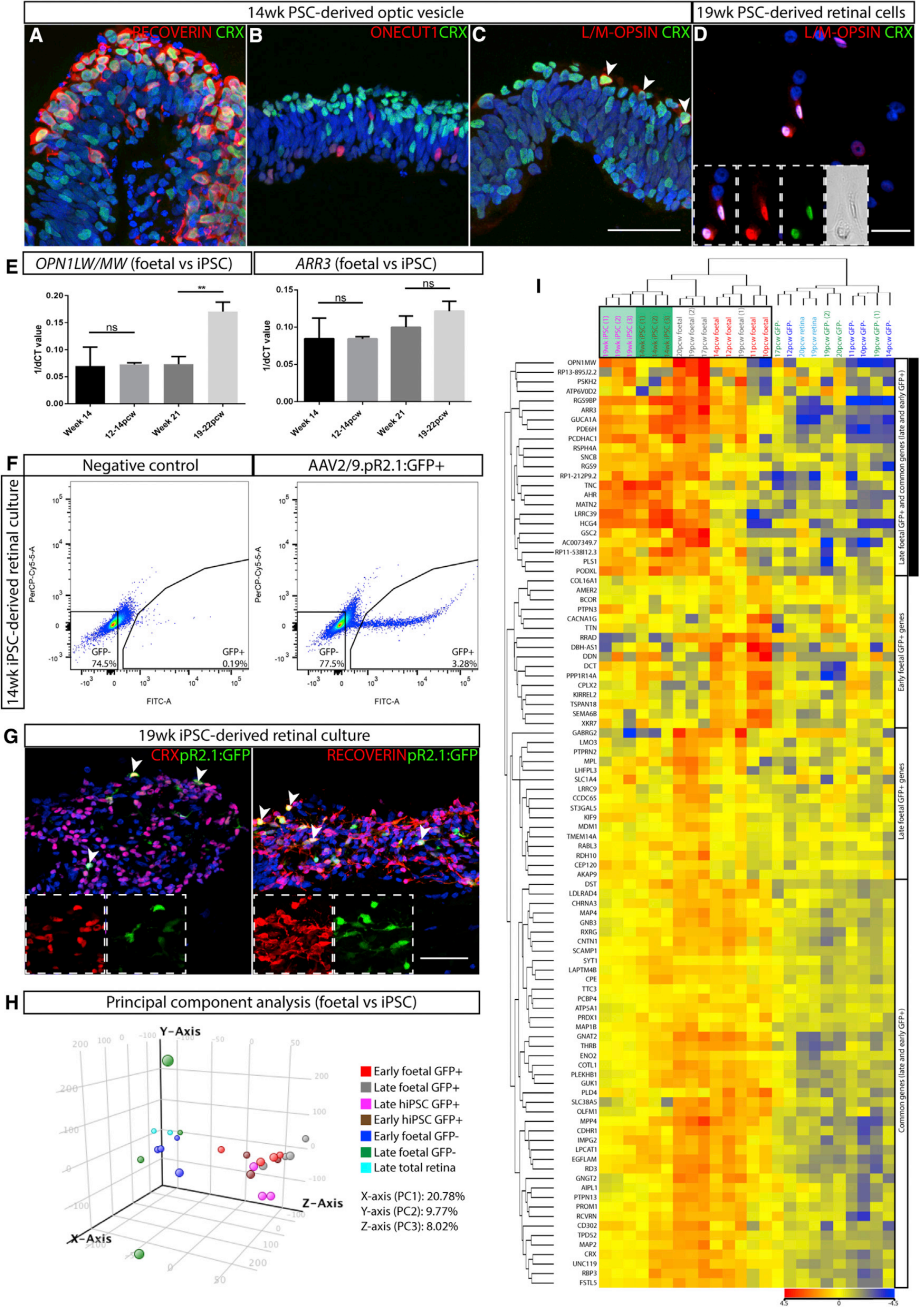
Generation of PSC-Derived Cone Photoreceptors via 3D Retinal Differentiation Culture System (A–D) Immunohistochemistry analysis showing detection of photoreceptor markers, RECOVERIN and CRX (A), and cone markers, ONECUT1 (B) and L/M-OPSIN (C), within 14-week PSC-derived vesicles. At 26 weeks, L/M-OPSIN can also be detected within dissociated cultures (D), which also express CRX (C and D; arrowheads). (E) Comparative qRT-PCR analysis of *OPN1LW/MW* and *ARR3* at age-matched time points between fetal and iPSC-derived retinal samples (14 weeks and 12–14 pcw; 19 weeks and 19–22 pcw), ^∗∗^p = 0.0008. All error bars represent the SD. (F) Representative FACS traces of negative control and 14 weeks iPSC-derived retinal differentiation culture labeled with the AAV2/9.pR2.1:GFP reporter. (G) Representative images showing co-labeling of iPSC GFP+ cells with photoreceptor markers CRX and RECOVERIN (arrowheads) within 20 week retinal differentiation culture. (H) PCA plot of fetal and iPSC-derived GFP+ samples based on total gene expression. (I) Heatmap representation of hierarchical clustering analysis performed with fetal and iPSC-derived samples based on the cone-enriched gene signature. Genes featured on the heatmap represent all the early (16 genes), late (37 genes), and most enriched genes common to early and late cones (44 genes with p < 0.05, highest fold changes and high expression from early and late GFP+ versus GFP− comparisons). Scale bar, 50 μm. See Figure S5 and Table S2.

Having defined the transcriptomic signature of a human fetal L/M-opsin cone photoreceptor population, we applied the same approach to human iPSC-derived cone photoreceptor cells to assess the degree of similarity between *in vivo* and *in vitro* generated cones. To generate cone photoreceptors from iPSCs, we used a retinal organoid differentiation culture system (Meyer et al., 2009) that generates optic vesicle structures containing RECOVERIN and CRX-expressing photoreceptor cells from week 6 of differentiation (Figure S5A). Expression of cone markers, detected in the human fetal retinal sample series, were confirmed by qRT-PCR (*RXRG, THRB, SALL3, ONECUT1*, *OPN1LW/MW, ARR3,* and *GNAT2;* Figure S5B). *OPN1LW/MW* expression was upregulated by week 14 of differentiation and maintained into later stages of culture (weeks 21–28). Immunohistochemistry revealed labeling for photoreceptor markers, RECOVERIN and CRX, in addition to cone markers, ONECUT1 and L/M-OPSIN, within optic vesicle structures by week 14 (Figures 4A–4C) and in dissociated late-stage cultures for L/M-OPSIN (Figure 4D). Comparable levels of *OPN1LW/MW* and *ARR3* expression were detected at early (12–14 pcw) and late (19–20 pcw) developmental time points compared with weeks 14 and 21 iPSC-derived retinal differentiation cultures, respectively; however, OPN1LW/MW showed significantly higher expression within the late-stage human fetal retina (Figure 4E).

The AAV2/9.pR2.1:GFP virus was used to transduce early (weeks 14–15) and late (weeks 19–20) iPSC-derived retinal differentiation cultures, and pR2.1:GFP+ cells were isolated for RNA-seq analysis using FACS (Figure 4F) and showed co-labeling with photoreceptor markers, CRX and RECOVERIN (Figure 4G, white arrows). Principal component analysis showed clustering of the fetal and iPSC-derived GFP+ samples together, away from total fetal retina and GFP− samples (Figure 4H). Hierarchical clustering analysis performed with the newly identified fetal cone-enriched gene signature revealed that iPSC-derived GFP+ samples were most similar to the fetal GFP+ samples (Figure 4I; black box). Specifically, genes enriched within the late fetal cones show the most comparable expression profiles within both weeks 14–15 and 19–20 iPSC-derived GFP+ samples and the late-stage fetal samples (Figure 4I, 21/37 genes; top cluster; filled black box). Notably, the early cone-enriched genes (Figure 4I, 16 genes; second cluster) showed overall low expression within the iPSC-derived cells, together with the remainder of the late cone-enriched genes and the common cone gene set (Figure 4I, third and fourth clusters). Overall, these data suggest a cone identity of the iPSC-derived cells and provide valuable information on their stage of differentiation and degree of similarity to fetal cones.

### Identification and Application of Cell Surface Markers to Isolate Human Fetal and iPSC-Derived Cone Photoreceptors

**Figure 5:**
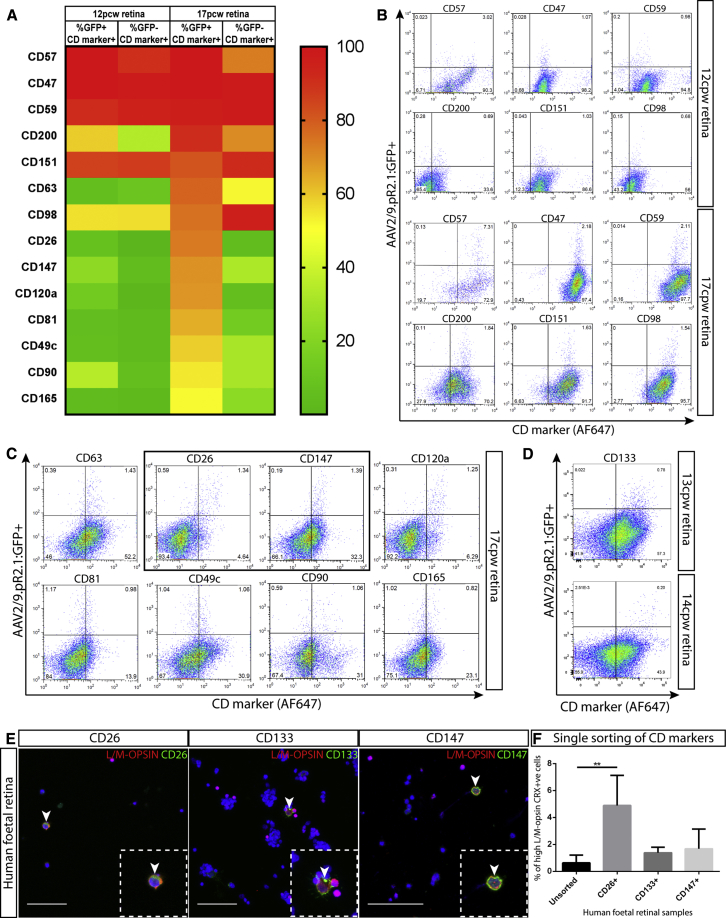
Identifying and Profiling CD Makers in the Human Fetal Retina (A) Heatmap showing the cell percentage labeled by the 14 CD markers identified from the antibody screening panels. GFP+/CD marker+ percentage is representative of the total labeled pR2.1:GFP+ population and the GFP−/CD marker proportion is based on the remaining retinal cell population. (B) Flow cytometry traces of the 6 CD markers labeling 12 and 17 pcw pR2.1:GFP+ cells. (C) Flow cytometry traces of the 8 CD markers labeling GFP+ cells within the 17 pcw retina; CD26 and CD147 are highlighted by the black box. (D) Flow cytometry traces of CD133 labeling of 13 and 14 pcw pR2.1:GFP+ cells. (E) CD26, CD133, and CD147 labeling of L/M-OPSIN-expressing cells (arrowheads indicate double-labeled cells) in the 17 pcw fetal retina via immunocytochemistry. Scale bar, 50 μm. (F) Counting of L/M-OPSIN/CRX+ cells in single FACS experiments with CD26, CD133, and CD147 (n = 3; 17–20 pcw), ^∗∗^p = 0.0044. All error bars represent the standard deviation. See Table S5.

Next, we used a dual approach to identify cell surface markers expressed by fetal pR2.1:GFP+ cells, which could provide tools to enrich for human cone photoreceptors without the need for genetic manipulation. We created a cell surface marker protein query database of 3,367 putative cell surface proteins, which was used to identify 99 and 170 potential cell surface marker genes enriched in the early and late fetal pR2.1:GFP+ cells, respectively (Table S5). Of these, 16 were cluster of differentiation (CD) markers (Table S5; highlighted); 13 and 7 were identified in the late and early enriched cone gene sets respectively, with 4 CD markers, *DPP4* (CD26), *PROM1* (CD133), *ITGA4* (CD49d), and *JAM2* (CD322), featuring in both populations. Of these, *DPP4* (CD26) and *PROM1* (CD133) were highly expressed and significantly upregulated in the late-stage cones and the remainder represent potential cone cell surface markers (Table S5). In addition, we directly screened early (12 pcw) and late (17 pcw) fetal retinae labeled with the AAV2/9 pR2.1:GFP reporter for 242 different human CD marker antibodies using the BD Lyoplate screening panels. We identified markers that labeled and caused a discrete flow cytometry shift of at least 50% of the pR2.1:GFP+ cell population (Figure 5A). Six CD markers (CD57, CD47, CD59, CD200, CD151, and CD98) were common across the 12 and 17 pcw samples (Figure 5B), and eight additional markers (CD63, CD26, CD147, CD120a, CD81, CD49c, CD90, and CD165) labeled the 17 pcw pR2.1:GFP+ cells (Figure 5C). CD26 overlapped with the CD markers identified from the cone gene signature and the remaining 12 CD markers were detected within the cone transcriptome data, except CD165. Notably, some CD markers displayed greater specificity to pR2.1:GFP+ cells at later fetal time points, i.e., labeling a higher percentage of GFP+ cells and lower percentage of GFP− cells, including markers CD26 and CD147 (Figure 5C, black box). CD133 robustly labeled 13 and 14 pcw pR2.1:GFP+ cells (Figure 5D).

**Figure 6:**
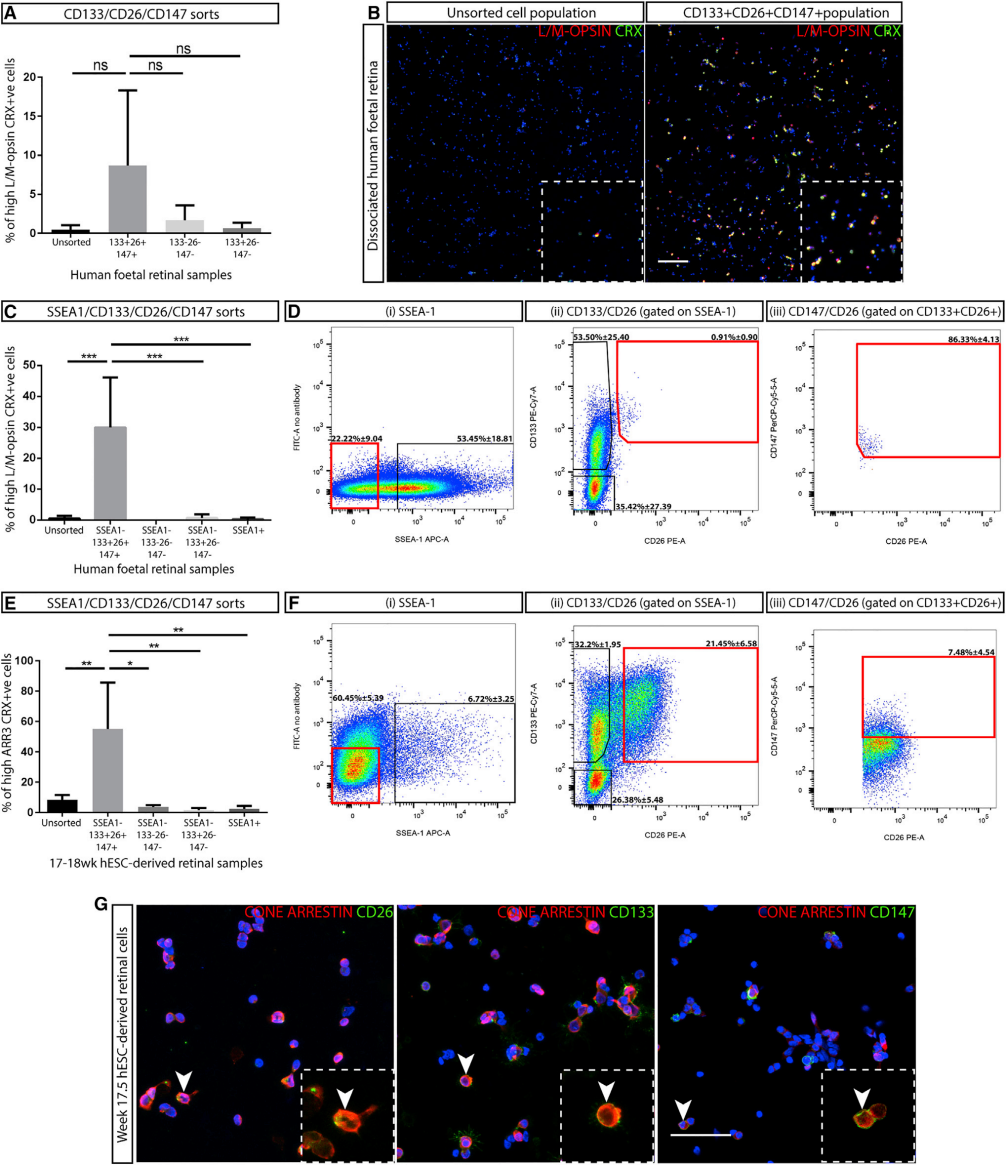
Application of Cone CD Marker Sorting in Human Fetal and ESC-Derived Retina (A) Counting of fetal L/M-OPSIN/CRX+ cells post FACS with the CD133/CD26/CD147 combination (n = 4); p = 0.1067. ns, not significant. All error bars represent the SD. (B) Representative images of L/M-OPSIN/CRX immunocytochemistry within the unsorted and CD133+/CD26+/CD147+ sorted cell population. Scale bar, 100 μm. (C) Counting of fetal L/M-OPSIN/CRX+ cells post FACS with the SSEA1/CD133/CD26/CD147 combination (n = 4; 17–20 pcw); ^∗∗∗^p < 0.0002. All error bars represent the SD. (D) Representative FACS traces for the SSEA1/CD133/CD26/CD147 combination within the fetal retinae, with cell population percentages. The red rectangular outlines represent gated cell populations in the FACS experiments. (E) Counting of ESC-derived CONE ARRESTIN/CRX+ cells post FACS with the SSEA1/CD133/CD26/CD147 combination (n = 4, 17–18 weeks); ^∗^p = 0.0146, ^∗∗^p = 0.0036. All error bars represent the SD. (F) Representative FACS traces for the SSEA1/CD133/CD26/CD147 combination for the ESC-derived retinal cultures. Cell population percentages are provided for (D) and (F), mean represents ± SD. (G) Immunocytochemistry of CD26, CD133, CD147, and CONE ARRESTIN within 17.5 weeks dissociated ESC-derived retinal cells (white arrowheads indicate co-localization). Scale bar, 50 μm.

CD26, CD133, and CD147 were selected for testing to determine if they could enrich for late-stage human L/M-opsin cones. Double immunocytochemistry on dissociated human fetal retina revealed that CD26, CD133, and CD147 co-label with some, but not all, L/M-OPSIN-expressing cells (Figure 5E, white arrows). Some L/M-OPSIN-negative cells were also labeled by the CD markers. In FACS experiments, the CD markers individually labeled a proportion of the total fetal retinal cells (CD26+ 2.9% ± 1.6%; CD147+ 23.1% ± 12.4% and CD133+ 71.1% ± 7.2%); these sorted cells showed a greater percentage of L/M-OPSIN and CRX+ cells compared with the unsorted population (CD26+ 4.9% ± 2.23%; CD133+ 1.38% ± 0.41%; CD147+ 1.67% ± 1.47%, unsorted 0.63% ± 0.59%), with the CD26+ population showing statistical significance (Figure 5F). This overall low level of L/M-opsin cone enrichment prompted the combined sorting of these CD markers to assess if a greater enrichment could be achieved. We detected a triple-positive cell population for all three CD markers via flow cytometry within late-stage fetal retinae and performing immunocytochemistry on this population post FACS revealed enrichment of L/M-OPSIN/CRX+ cells (CD133+/CD26+/CD147+ 8.69% ± 9.62%) compared with the other collected cells (CD133–CD26–CD147– 1.67% ± 1.93%; CD133+/CD26–/CD147– 0.64% ± 0.72%) and the unsorted population (0.45% ± 0.59%; Figures 6A and 6B). To enhance enrichment, we added a further CD marker for negative selection, SSEA-1, which was not detected in pR2.1:GFP+ cells in the proteomic analysis (Figure S6) and has been previously used to remove mitotically active cells (Lakowski et al., 2015, Tucker et al., 2011). By sorting fetal retinal cells negative for SSEA-1, but positive for CD133, CD26, and CD147, an improvement in the percentage of L/M-OPSIN/CRX+ cells within the enriched cell population was achieved (30% ± 16.13%; p < 0.0002) when compared with the unsorted population (0.54% ± 0.8%) and the remaining sorted cell populations (CD133–/CD26–/CD147–/SSEA1– 0%; CD133+/CD26–/CD147–/SSEA1– 0.69% ± 1.2%; and SSEA1+ 0.27% ± 0.49%; Figures 6C and 6D).

Finally, we tested the fetal cone CD marker combination on stem cell-derived retinal differentiation cultures generated using the standard protocol (Meyer et al., 2009) for iPSC, or a newly developed protocol for ESC retinal differentiation, which shows improved levels of cone photoreceptor maturation (Gonzalez-Cordero et al., 2017) (Figures S7A and S7B), to assess if a similar CD marker-labeled population of cells was generated by the *in vitro* systems. Figure S7C shows representative flow cytometry traces comparing the SSEA1–CD133+ CD26+ CD147+-labeled populations between fetal and PSC-derived retina. The ESC-derived retinal cultures using the newly developed protocol showed the most similar FACS trace to those observed from the fetal retina and was used for subsequent cell-sorting experiments (week 17–18 of culture, n = 3). Performing immunocytochemistry on this sorted cell population revealed a significant enrichment of CONE ARRESTIN/CRX+ cells (55.1% ± 30.7%; p = 0.0036) compared with the unsorted (8.4% ± 3.1%) and additional cell populations (SSEA1– CD133– CD26– CD147– 3.7% ± 1.2%; SSEA1– CD133+ CD26– CD147– 2.3% ± 0.8; SSEA1+ 2.4% ± 2.0%; Figures 6E and 6F). In addition, CONE ARRESTIN-expressing cells labeled by CD26, CD133, and CD147 were readily detected within dissociated ESC-derived retinal cells (Figure 6G, white arrows), similar to observations within the fetal retina. These data indicate that the cone CD marker panel discovered within the fetal retina can be applied to ESC-derived retinal differentiation cultures to enrich for human cone photoreceptor cells.

## Discussion

Two important pre-clinical evaluations that need to be addressed for the development of a human cone cell-replacement therapy include assessing whether iPSC-derived cone cells recapitulate a *bona fide* cone photoreceptor transcriptome and the development of a cone cell isolation strategy. Here, we provide insight into the human fetal L/M-opsin cone transcriptome and used this as a baseline reference to show similarities in cone gene expression within iPSC-derived photoreceptors. From these data, we also developed a cell surface marker sorting strategy for the enrichment of human L/M-opsin cone photoreceptors that can be translated to stem cell-derived retinal differentiation culture system.

Our definition of a unique cone-enriched gene signature for human fetal cone photoreceptors relied on the use of an AAV2/9.pR2.1:GFP reporter. This viral reporter showed preferential labeling of human L/M-opsin cone photoreceptors. Rod-associated and S-opsin transcripts were also detected, consistent with previous reports of shared molecular markers between different cone and rod cells (Applebury et al., 2000, Cornish et al., 2004, Craft et al., 2014, Kim et al., 2016). These data suggest a plastic or “noisy” nature of some human photoreceptors, which may continue to express transcripts of other photoreceptor types during fetal development. Exploring the heterogeneity of individual cone cells at a single time point revealed their separation based on maturity, identifying differences in gene expression profiles of naive and more mature cones, rather than distinguishing cells based on their specific type of cone opsin gene expression. This difference likely reflects the striking gradient of cone photoreceptor maturation observed across the central-peripheral axis in the human fetal retina (Figures 1 and S1) (Xiao and Hendrickson, 2000).

Both the bulk and single-cell RNA-seq data validated 93 cone maturation genes (Table S2, green highlighted genes), including several not previously defined to cone cells. Comparison of our cone-enriched gene signature with pre-existing human adult macula gene expression analyses showed that a number of genes overlap, including transcription factor *NR2F6* and the photoreceptor ribbon synapse protein, *CPLX4* (Bowes Rickman et al., 2006, Sharon et al., 2002). However, many identified genes were not previously reported in adult macula studies (Hornan et al., 2007, Whitmore et al., 2014), which could be due to differences in samples ages, or their inclusion of other retinal cells. Several genes expressed by human ESC-derived CRX.GFP cells (Kaewkhaw et al., 2015), including *SLC38A5, VTN*, and *AMER2*, were also shared with our cone-enriched gene signature.

In the direct comparative transcriptome analysis performed using the cone gene signature, we found similar gene expression profiles between late fetal and iPSC-derived cone cells. However, not all late enriched cone genes showed equivalent levels of expression within the iPSC-derived cells, suggesting an intermediate stage of cone cell differentiation and a relative delay in their maturation. Hence, this approach provides an informative insight into the similarities and differences in gene expression between *in*-*vivo-* and *in*-*vitro*-derived cells, and important benchmarking data, which is critical for pre-clinical assessment of stem cell-derived cells for use in clinical therapies.

Toward developing a cone cell purification strategy, we characterized putative cell surface marker genes and CD marker proteins expressed within the AAV2/9.pR2.1:GFP+ human cone populations, providing a benchmarking tool for the differentiation status of retinal cultures. We evaluated a cell isolation strategy for the late cone population, based on the presence of identified cell surface molecules. Both *ST3GAL5* and *EPHA10* genes, previously identified as putative cone surface marker from RNA-seq analysis of human ESC-derived CRX-expressing cells (Kaewkhaw et al., 2015), featured within our significantly enriched late cone dataset. However, CD73, previously used to isolate cone-like cells from Nrl^−/−^ mouse retina (Santos-Ferreira et al., 2015), was not upregulated in the human cones. We found no cell surface markers that exclusively labeled the pR2.1:GFP+ cone population and instead identified CD markers, such as CD26, CD147, and CD133, which labeled a percentage of cone cells as well as other retinal cells. Together with the rarity of cone cells within the human retina, this presents a considerable challenge to achieve high levels of enrichment. Nevertheless, by combining CD markers for positive cone selection (CD26, CD147, and CD133) and negative selection of undesirable cells (SSEA-1), we demonstrate the enrichment of human L/M-opsin cone cells from the human fetal retina. The average enrichment achieved using this combination (30% ± 16.13%) suggests further optimization would be required to increase the purity of cone cell isolation for a clinical transplantation therapy. However, these data demonstrate the feasibility of using a cell surface molecule approach to enrich for human cone photoreceptors. Furthermore, using a new ESC-derived retinal differentiation culture system with higher levels of cone differentiation (Gonzalez-Cordero et al., 2017), we demonstrate the same fetal cone sorting strategy can be applied to provide an enrichment of ESC-derived cone photoreceptors. Together with the identification of a human L/M-opsin cone-enriched gene signature, this study provides an initial pre-clinical assessment comparing *bona fide* and stem cell-derived cones toward developing a cone cell therapy, which may also be informative for other applications, including *in vitro* retinal disease modeling and basic research into human cone photoreceptor development.

## Experimental Procedures

Full experimental methods are provided within Supplemental Experimental Procedures.

### Human Tissue

Human fetal eyes were obtained from the Joint Medical Research Council UK (grant no. G0700089)/Wellcome Trust (grant no. GR082557) Human Developmental Biology Resource (http://www.hdbr.org/), and human adult eyes were obtained from Moorfields Biobank with ethics approval. iPSCs (NCUS:7) were generated by the NIHR Cambridge Biomedical Research Centre hiPSC Core Facility from an individual's skin fibroblasts with normal ophthalmological examination and with ethics approval. Work on human ESC lines was approved by the UK Stem Cell Bank Steering Committee (Medical Research Council, London, UK).

### Application of AAV2/9.pR2.1:GFP Reporter

AAV2/9.pR2.1:GFP virus was added to human fetal retinal explants and iPSC-derived retinal differentiation cultures at an MOI of 40,000–45,000. pR2.1.GFP+ cells were sorted using the BD FACSAria III cell sorter for RNA-seq analysis. CD marker profiles of pR2.1.GFP+ labeled cells were detected using BD Lyoplate screening panels and the BD FACSCalibur.

### Total and Single mRNA-Seq and Analysis

The Illumina NextSeq 500 system was used with a targeted sequencing depth of 17 million 43 bp paired end per sample for the bulk RNA-seq and 2 million 75 bp paired end reads per sample for the single-cell RNA-seq. Adjusted p values < 0.05 were used for bulk and single-cell data to determine significance.

### Statistics

ANOVA with Bonferroni correction for multiple testing was used to determine statistical significance using GraphPad Prism 6. Adjusted p values are stated within figure legends and all means are displayed ±SD. For qRT-PCR analysis, three biological samples were used for each time point excluding Figure 1, where biological sample is 1 due to the limited availability of fetal tissue. For FACS CD marker analysis, four independent fetal/ESC-derived retinal samples were analyzed. At least 50 cells were counted for FACS cell populations using the ImageJ software.

## Author Contributions

E.W., conception and design, collection and/or assembly of data, data analysis and interpretation, manuscript writing. J.L., conception and design, collection and/or assembly of data, data analysis and interpretation. V.D.F., collection and/or assembly of data. D.B., collection and/or assembly of data. A.G.-C., collection and/or assembly of data. A.T.L.L., data analysis and interpretation. M.E., data analysis and interpretation. A.P., data analysis. E.C., collection and/or assembly of data. K.K., collection and/or assembly of data. A.N., collection and/or assembly of data. F.M., data analysis and interpretation. M.H., collection and/or assembly of data. D.T.J., data analysis. J.C.M., data analysis and interpretation. R.R.A., conception and design, manuscript writing, financial support. J.C.S., conception and design, data analysis and interpretation, manuscript writing, final approval of manuscript, financial support.

## Accession Numbers

The accession number for the bulk RNA-seq data reported in this paper is ArrayExpress: E-MTAB-6057. The accession number for the single cell RNA-seq data reported in this paper is ArrayExpress: E-MTAB-6058.

## Supplemental Information

Document S1. Supplemental Experimental Procedures and Figures S1–S7

Document S2. Tables S1–S5

Document S3. Article plus Supplemental Information

## References

[bib1] Applebury M.L., Antoch M.P., Baxter L.C., Chun L.L., Falk J.D., Farhangfar F., Kage K., Krzystolik M.G., Lyass L.A., Robbins J.T. (2000). The murine cone photoreceptor: a single cone type expresses both S and M opsins with retinal spatial patterning. Neuron.

[bib2] Bartsch U., Oriyakhel W., Kenna P.F., Linke S., Richard G., Petrowitz B., Humphries P., Farrar G.J., Ader M. (2008). Retinal cells integrate into the outer nuclear layer and differentiate into mature photoreceptors after subretinal transplantation into adult mice. Exp. Eye Res..

[bib3] Bessant D.A., Ali R.R., Bhattacharya S.S. (2001). Molecular genetics and prospects for therapy of the inherited retinal dystrophies. Curr. Opin. Genet. Dev..

[bib4] Bowes Rickman C., Ebright J.N., Zavodni Z.J., Yu L., Wang T., Daiger S.P., Wistow G., Boon K., Hauser M.A. (2006). Defining the human macula transcriptome and candidate retinal disease genes using EyeSAGE. Invest. Ophthalmol. Vis. Sci..

[bib5] Cai H., Fields M.A., Hoshino R., Priore L.V. (2012). Effects of aging and anatomic location on gene expression in human retina. Front. Aging Neurosci..

[bib6] Cornish E.E., Xiao M., Yang Z., Provis J.M., Hendrickson A.E. (2004). The role of opsin expression and apoptosis in determination of cone types in human retina. Exp. Eye Res..

[bib7] Craft C.M., Huang J., Possin D.E., Hendrickson A. (2014). Primate short-wavelength cones share molecular markers with rods. Adv. Exp. Med. Biol..

[bib8] de Melo J., Peng G.H., Chen S., Blackshaw S. (2011). The Spalt family transcription factor Sall3 regulates the development of cone photoreceptors and retinal horizontal interneurons. Development.

[bib9] Decembrini S., Martin C., Sennlaub F., Chemtob S., Biel M., Samardzija M., Moulin A., Behar-Cohen F., Arsenijevic Y. (2017). Cone genesis tracing by the Chrnb4-EGFP mouse line: evidences of cellular material fusion after cone precursor transplantation. Mol. Ther..

[bib10] Eberle D., Santos-Ferreira T., Grahl S., Ader M. (2014). Subretinal transplantation of MACS purified photoreceptor precursor cells into the adult mouse retina. J. Vis. Exp..

[bib11] Eberle D., Schubert S., Postel K., Corbeil D., Ader M. (2011). Increased integration of transplanted CD73-positive photoreceptor precursors into adult mouse retina. Invest. Ophthalmol. Vis. Sci..

[bib12] Emerson M.M., Surzenko N., Goetz J.J., Trimarchi J., Cepko C.L. (2013). Otx2 and Onecut1 promote the fates of cone photoreceptors and horizontal cells and repress rod photoreceptors. Dev. Cell.

[bib13] Fei Y., Hughes T.E. (2001). Transgenic expression of the jellyfish green fluorescent protein in the cone photoreceptors of the mouse. Vis. Neurosci..

[bib14] Fujieda H., Bremner R., Mears A.J., Sasaki H. (2009). Retinoic acid receptor-related orphan receptor alpha regulates a subset of cone genes during mouse retinal development. J. Neurochem..

[bib15] Gonzalez-Cordero A., Kruczek K., Naeem A., Fernando M., Kloc M., Ribeiro J., Goh D., Duran Y., Blackford S.J.I., Abelleira-Hervas L. (2017). Recapitulation of human retinal development from human pluripotent stem cells generates transplantable populations of cone photoreceptors. Stem Cell Reports.

[bib16] Hendrickson A., Bumsted-O'brien K., Natoli R., Ramamurthy V., Possin D., Provis J. (2008). Rod photoreceptor differentiation in fetal and infant human retina. Exp. Eye Res..

[bib17] Hofer H., Carroll J., Neitz J., Neitz M., Williams D.R. (2005). Organization of the human trichromatic cone mosaic. J. Neurosci..

[bib18] Hornan D.M., Peirson S.N., Hardcastle A.J., Molday R.S., Cheetham M.E., Webster A.R. (2007). Novel retinal and cone photoreceptor transcripts revealed by human macular expression profiling. Invest. Ophthalmol. Vis. Sci..

[bib19] Kaewkhaw R., Kaya K.D., Brooks M., Homma K., Zou J., Chaitankar V., Rao M., Swaroop A. (2015). Transcriptome dynamics of developing photoreceptors in three-dimensional retina cultures recapitulates temporal sequence of human cone and rod differentiation revealing cell surface markers and gene networks. Stem Cells.

[bib20] Kim J.W., Yang H.J., Oel A.P., Brooks M.J., Jia L., Plachetzki D.C., Li W., Allison W.T., Swaroop A. (2016). Recruitment of rod photoreceptors from short-wavelength-sensitive cones during the evolution of nocturnal vision in mammals. Dev. Cell.

[bib21] Komaromy A.M., Alexander J.J., Cooper A.E., Chiodo V.A., Glushakova L.G., Acland G.M., Hauswirth W.W., Aguirre G.D. (2008). Targeting gene expression to cones with human cone opsin promoters in recombinant AAV. Gene Ther..

[bib22] Lakowski J., Gonzalez-Cordero A., West E.L., Han Y.T., Welby E., Naeem A., Blackford S.J., Bainbridge J.W., Pearson R.A., Ali R.R., Sowden J.C. (2015). Transplantation of photoreceptor precursors isolated via a cell surface biomarker panel from embryonic stem cell-derived self-forming retina. Stem Cells.

[bib23] Lakowski J., Han Y.T., Pearson R.A., Gonzalez-Cordero A., West E.L., Gualdoni S., Barber A.C., Hubank M., Ali R.R., Sowden J.C. (2011). Effective transplantation of photoreceptor precursor cells selected via cell surface antigen expression. Stem Cells.

[bib24] Lamba D.A., Gust J., Reh T.A. (2009). Transplantation of human embryonic stem cell-derived photoreceptors restores some visual function in Crx-deficient mice. Cell Stem Cell.

[bib25] Li M., Jia C., Kazmierkiewicz K.L., Bowman A.S., Tian L., Liu Y., Gupta N.A., Gudiseva H.V., Yee S.S., Kim M. (2014). Comprehensive analysis of gene expression in human retina and supporting tissues. Hum. Mol. Genet..

[bib26] Li Q., Timmers A.M., Guy J., Pang J., Hauswirth W.W. (2008). Cone-specific expression using a human red opsin promoter in recombinant AAV. Vision Res.

[bib27] Lun A.T., Mccarthy D.J., Marioni J.C. (2016). A step-by-step workflow for low-level analysis of single-cell RNA-seq data with Bioconductor. F1000Res..

[bib28] MacLaren R.E., Pearson R.A., Macneil A., Douglas R.H., Salt T.E., Akimoto M., Swaroop A., Sowden J.C., Ali R.R. (2006). Retinal repair by transplantation of photoreceptor precursors. Nature.

[bib29] Meyer J.S., Shearer R.L., Capowski E.E., Wright L.S., Wallace K.A., Mcmillan E.L., Zhang S.C., Gamm D.M. (2009). Modeling early retinal development with human embryonic and induced pluripotent stem cells. Proc. Natl. Acad. Sci. USA.

[bib30] Nakano T., Ando S., Takata N., Kawada M., Muguruma K., Sekiguchi K., Saito K., Yonemura S., Eiraku M., Sasai Y. (2012). Self-formation of optic cups and storable stratified neural retina from human ESCs. Cell Stem Cell.

[bib31] Nathans J., Davenport C.M., Maumenee I.H., Lewis R.A., Hejtmancik J.F., Litt M., Lovrien E., Weleber R., Bachynski B., Zwas F. (1989). Molecular genetics of human blue cone monochromacy. Science.

[bib32] Nathans J., Thomas D., Hogness D.S. (1986). Molecular genetics of human color vision: the genes encoding blue, green, and red pigments. Science.

[bib33] Ng L., Hurley J.B., Dierks B., Srinivas M., Salto C., Vennstrom B., Reh T.A., Forrest D. (2001). A thyroid hormone receptor that is required for the development of green cone photoreceptors. Nat. Genet..

[bib34] Pearson R.A., Barber A.C., Rizzi M., Hippert C., Xue T., West E.L., Duran Y., Smith A.J., Chuang J.Z., Azam S.A. (2012). Restoration of vision after transplantation of photoreceptors. Nature.

[bib35] Pearson R.A., Gonzalez-Cordero A., West E.L., Ribeiro J.R., Aghaizu N., Goh D., Sampson R.D., Georgiadis A., Waldron P.V., Duran Y. (2016). Donor and host photoreceptors engage in material transfer following transplantation of post-mitotic photoreceptor precursors. Nat. Commun..

[bib36] Reichman S., Terray A., Slembrouck A., Nanteau C., Orieux G., Habeler W., Nandrot E.F., Sahel J.A., Monville C., Goureau O. (2014). From confluent human iPS cells to self-forming neural retina and retinal pigmented epithelium. Proc. Natl. Acad. Sci. USA.

[bib37] Roberts M.R., Hendrickson A., Mcguire C.R., Reh T.A. (2005). Retinoid X receptor (gamma) is necessary to establish the S-opsin gradient in cone photoreceptors of the developing mouse retina. Invest. Ophthalmol. Vis. Sci..

[bib38] Roorda A., Williams D.R. (1999). The arrangement of the three cone classes in the living human eye. Nature.

[bib39] Santos-Ferreira T., Llonch S., Borsch O., Postel K., Haas J., Ader M. (2016). Retinal transplantation of photoreceptors results in donor-host cytoplasmic exchange. Nat. Commun..

[bib40] Santos-Ferreira T., Postel K., Stutzki H., Kurth T., Zeck G., Ader M. (2015). Daylight vision repair by cell transplantation. Stem Cells.

[bib41] Santos-Ferreira T., Volkner M., Borsch O., Haas J., Cimalla P., Vasudevan P., Carmeliet P., Corbeil D., Michalakis S., Koch E. (2016). Stem cell-derived photoreceptor transplants differentially integrate into mouse models of cone-rod dystrophy. Invest. Ophthalmol. Vis. Sci..

[bib42] Sharon D., Blackshaw S., Cepko C.L., Dryja T.P. (2002). Profile of the genes expressed in the human peripheral retina, macula, and retinal pigment epithelium determined through serial analysis of gene expression (SAGE). Proc. Natl. Acad. Sci. USA.

[bib43] Siegert S., Scherf B.G., Del Punta K., Didkovsky N., Heintz N., Roska B. (2009). Genetic address book for retinal cell types. Nat. Neurosci..

[bib44] Srinivas M., Ng L., Liu H., Jia L., Forrest D. (2006). Activation of the blue opsin gene in cone photoreceptor development by retinoid-related orphan receptor beta. Mol. Endocrinol..

[bib45] Suzuki S.C., Bleckert A., Williams P.R., Takechi M., Kawamura S., Wong R.O. (2013). Cone photoreceptor types in zebrafish are generated by symmetric terminal divisions of dedicated precursors. Proc. Natl. Acad. Sci. USA.

[bib46] Takahashi K., Tanabe K., Ohnuki M., Narita M., Ichisaka T., Tomoda K., Yamanaka S. (2007). Induction of pluripotent stem cells from adult human fibroblasts by defined factors. Cell.

[bib47] Tucker B.A., Park I.H., Qi S.D., Klassen H.J., Jiang C., Yao J., Redenti S., Daley G.Q., Young M.J. (2011). Transplantation of adult mouse iPS cell-derived photoreceptor precursors restores retinal structure and function in degenerative mice. PLoS One.

[bib48] Wang Y., Macke J.P., Merbs S.L., Zack D.J., Klaunberg B., Bennett J., Gearhart J., Nathans J. (1992). A locus control region adjacent to the human red and green visual pigment genes. Neuron.

[bib49] Whitmore S.S., Wagner A.H., Deluca A.P., Drack A.V., Stone E.M., Tucker B.A., Zeng S., Braun T.A., Mullins R.F., Scheetz T.E. (2014). Transcriptomic analysis across nasal, temporal, and macular regions of human neural retina and RPE/choroid by RNA-Seq. Exp. Eye Res..

[bib50] Xiao M., Hendrickson A. (2000). Spatial and temporal expression of short, long/medium, or both opsins in human fetal cones. J. Comp. Neurol..

[bib51] Zhong X., Gutierrez C., Xue T., Hampton C., Vergara M.N., Cao L.H., Peters A., Park T.S., Zambidis E.T., Meyer J.S. (2014). Generation of three-dimensional retinal tissue with functional photoreceptors from human iPSCs. Nat. Commun..

