# Supplementary material for: Isolation and Comparative Transcriptome Analysis of Human Fetal and iPSC-Derived Cone Photoreceptor Cells
Source: Stem Cell Reports. 2017 Nov 16;9(6):1898–915. doi: 10.1016/j.stemcr.2017.10.018 (PMC5785701; doi:10.1016/j.stemcr.2017.10.018)
Supplement: Document S1. Supplemental Experimental Procedures and Figures S1–S7 [file mmc1.pdf]

Stem Cell Reports, Volume *9*

Supplemental Information

Isolation and Comparative Transcriptome Analysis of Human Fetal and iPSC-Derived Cone Photoreceptor Cells

Emily Welby, Jorn Lakowski, Valentina Di Foggia, Dimitri Budinger, Anai Gonzalez-Cordero, Aaron T.L. Lun, Michael Epstein, Aara Patel, Elisa Cuevas, Kamil Kruczek, Arifa Naeem, Federico Minneci, Mike Hubank, David T. Jones, John C. Marioni, Robin R. Ali, and Jane C. Sowden

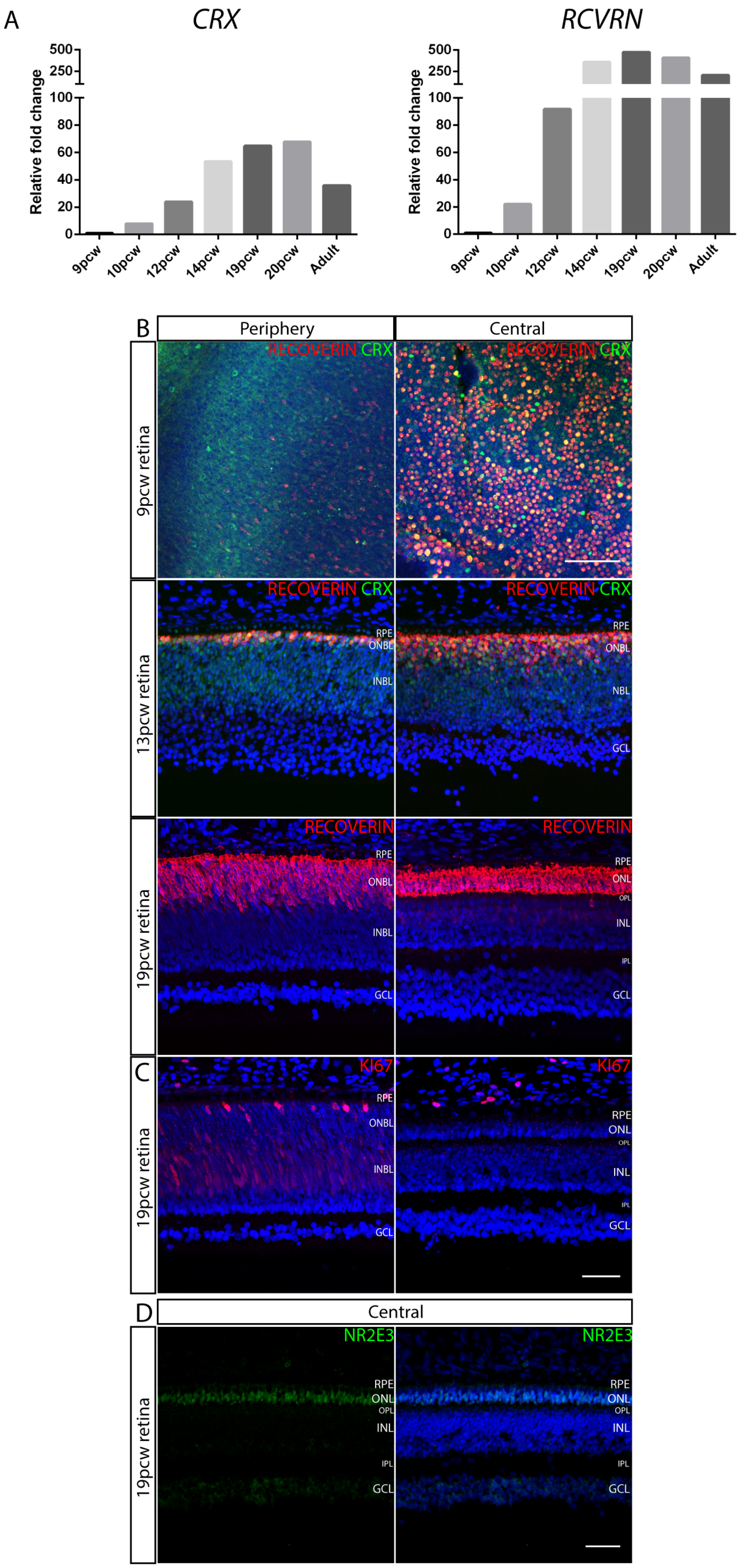

A
CRX
RCVRN
Relative fold change
500
250
100
80
60
40
20
0
9pcw
10pcw
12pcw
14pcw
19pcw
20pcw
Adult
B
Periphery
Central
9pcw retina
13pcw retina
19pcw retina
RECOVERIN CRX
RECOVERIN
RPE
ONBL
INBL
NBL
GCL
ONL
OPL
INL
IPL
C
KI67
D
NR2E3

Figure S1. Photoreceptor marker expression in the human peripheral and central retina (related to Figure 1).

(A) qRT PCR analysis of pan photoreceptor markers, cone-rod homeobox gene (*CRX*) and recoverin (*RCVRN*) in foetal (9pcw-20pcw) and adult retinal samples (n = 1 per timepoint). **(B)** RECOVERIN and CRX protein expression in the peripheral and central region of the developing retina (9pcw-19pcw). **(C)** Differences in retinal maturity are highlighted by KI67 immunostaining, present in the peripheral retina but absent in the central retina. **(D)** Rod marker, NR2E3, expression in the peripheral and central 19pcw retina. Wholemount image scale bar: 100µm; tissue section scale bar: 50µm. RPE, retinal pigment epithelium; ONBL, outer neuroblastic layer; INBL, inner neuroblastic layer; ONL, outer nuclear layer; OPL, outer plexiform layer; INL, inner nuclear layer; IPL, inner plexiform layer; GCL, ganglion cell layer.

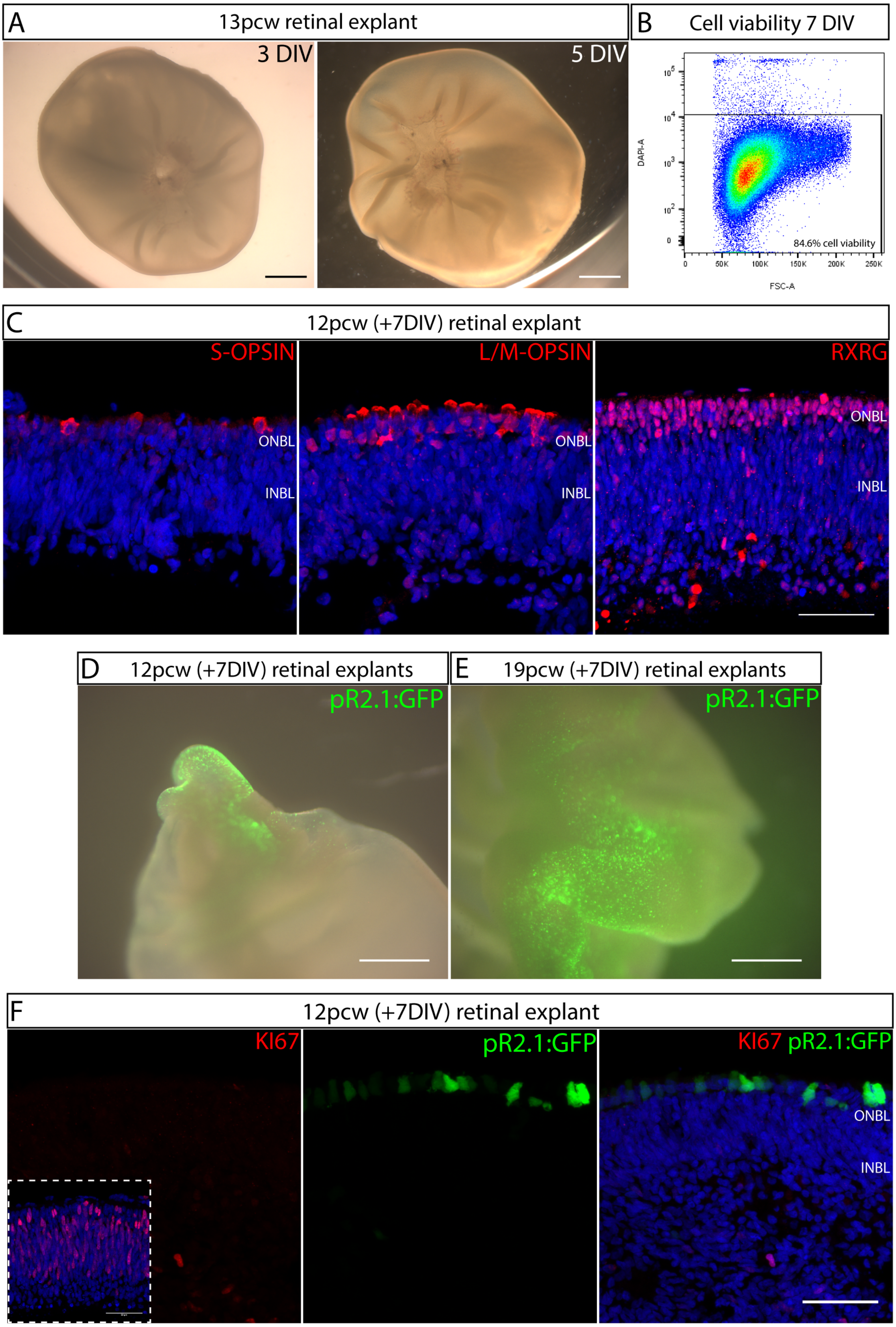

G

Marker	No. of GFP+ cells	No. of marker+ cells	No. of GFP+/marker+ cells	Percentage
L/M-OPSIN	53	93	29	54.7
RXRγ	84	233	84	100
S-OPSIN	44	15	0	0
NR2E3	55	143	0	0

Figure S2. AAV2/9.pR2.1:GFP reporter expression in human foetal retinal explant culture system (related to Figure 2).

(A) Brightfield wholemount images showing 13pcw retinal explant at 3 and 5 days *in vitro* (DIV) using a free floating culture method. **(B)** Dissociation and flow cytometry analysis of the explant on 7 DIV shows a high level of cell viability. **(C)** Representative images of 12pcw (+7DIV) retinal explant showing preserved cone photoreceptor marker staining (S-OPSIN, L/M-OPSIN, RXRG) and retinal integrity. Wholemount images showing pR2.1:GFP+ signal within cultured 12pcw **(D)** and 19pcw **(E)** retinal explants. **(F)** Sections of a 12pcw (+7DIV) retinal explant showing the absence of KI67 expression within pR2.1:GFP+ cells. Insert shows KI67 positive control for antibody. Scale bar for wholemount images of foetal retinal explants: 1mm; tissue section scale bar: 50µm. ONBL, outer neuroblastic layer; INBL, inner neuroblastic layer. **(G)** Counting of the marker + and GFP+ cells from immunofluorescence data in Figure 1L-O. Three sections of a 12pcw retinal explant transduced with the pR2.1:GFP reporter were counted for each immunostaining experiment (1 field of view per section where GFP+ cells and marker + cells could be seen). 0% of GFP+ cells co-labelled with NR2E3 (55 GFP+ cells counted in total), 0% of cells co-labelled with S-OPSIN (44 GFP+ cells counted in total), 100% of cells co-labelled with RXRG (84 GFP+/RXRG+ cells; 84 GFP+ cells counted in total) and 54.7% of cell co-labelled with L/M-OPSIN (29 GFP+/L/M-OPSIN+ cells; 53 GFP+ cells counted in total).

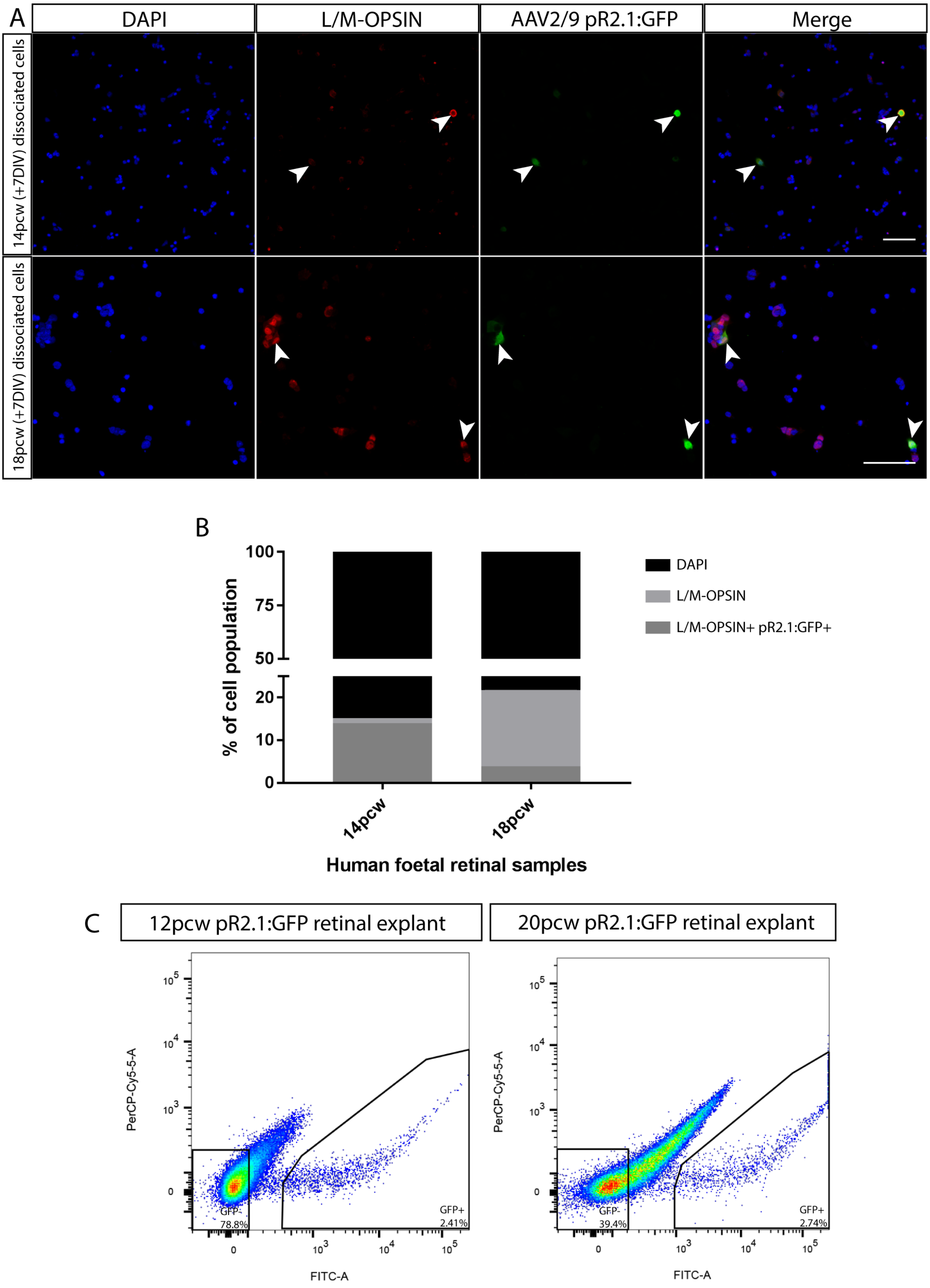

A
DAPI
L/M-OPSIN
AAV2/9 pR2.1:GFP
Merge
14pcw (+7DIV) dissociated cells
18pcw (+7DIV) dissociated cells
B
% of cell population
100
75
50
20
10
0
DAPI
L/M-OPSIN
L/M-OPSIN+ pR2.1:GFP+
14pcw
18pcw
Human foetal retinal samples
C
12pcw pR2.1:GFP retinal explant
20pcw pR2.1:GFP retinal explant
PerCP-Cy5-5-A
FITC-A
GFP-
78.8%
GFP+
2.41%
GFP-
39.4%
GFP+
2.74%

Figure S3. Efficiency of AAV2/9.pR2.1:GFP reporter and pR.2.1:GFP+ cell isolation via FACS (related to Figure 1 and 2).

(A) L/M-OPSIN immunostaining on dissociated pR2.1:GFP+ 14pcw and 18pcw (+7DIV) retinal explants show GFP+ cells co-label with L/M-OPSIN expression (white arrows). **(B)** Counting of these cells reveals the number of L/M-OPSIN+ cells and pR2.1:GFP+ cells in each sample. In test samples, reporter labelling efficiencies of 14% and 4% were achieved in 14pcw (30 GFP+/214 L/M-OPSIN+ cells) and 18pcw (9 GFP+/230 L/M-OPSIN+ cells) retinae, respectively. **(C)** Representative FACS traces of foetal pR2.1:GFP+ retinal explants showing gates used to collect GFP+ and GFP- cells for RNA seq.

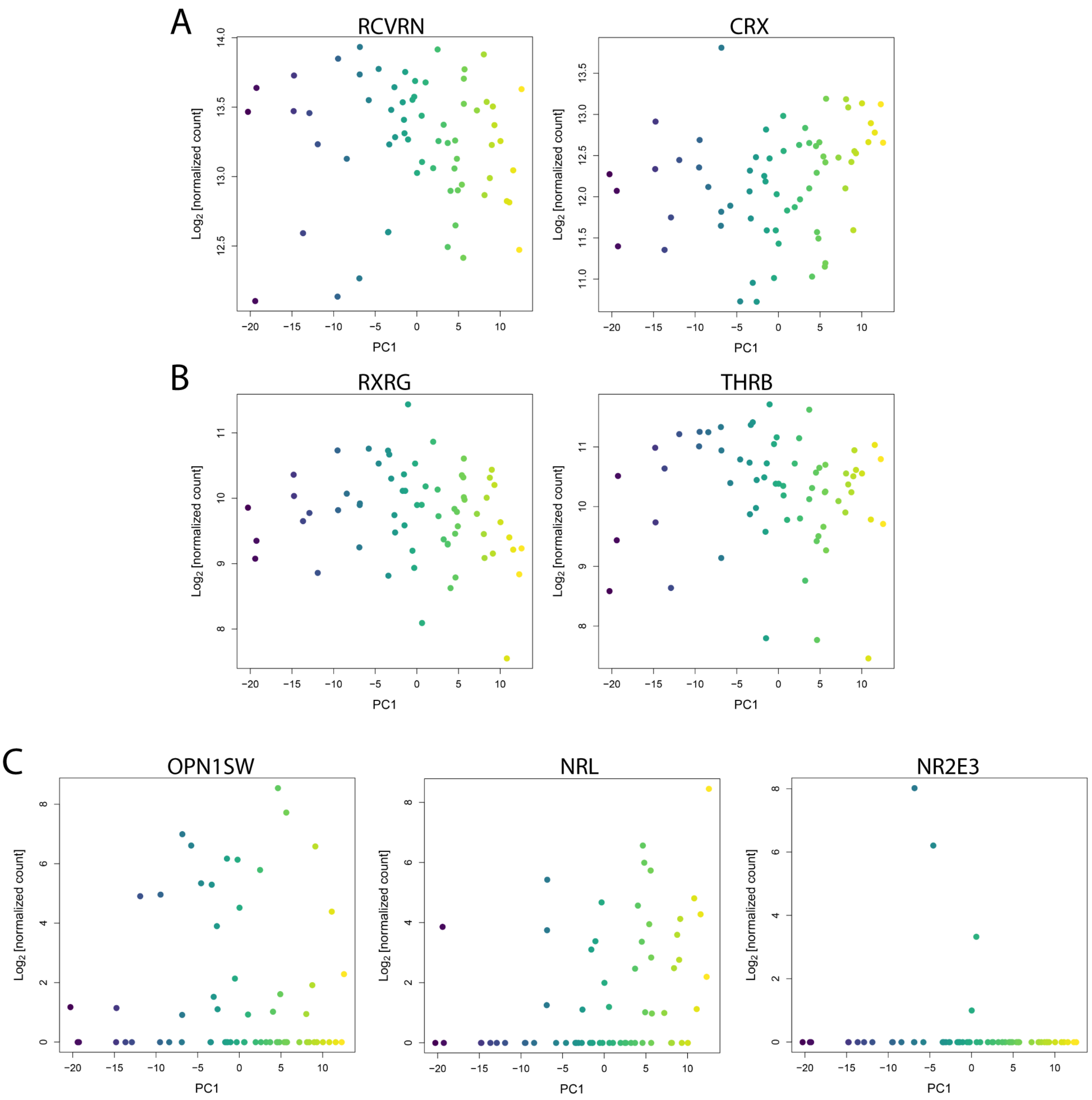
A
RCVRN
CRX
B
RXRG
THRB
C
OPN1SW
NRL
NR2E3
Log2 [normalized count]
PC1

Figure S4. Photoreceptor gene expression profile across single AAV2/9 pR2.1:GFP labelled cells (related to Figure 3).

Gene expression (log2 normalised counts) profiles of pan photoreceptor genes (*RCVRN* and *CRX*; **A)**, cone photoreceptor genes (*RXRG* and *THRB*; **B)**, S-cone (*OPN1SW*) and rod photoreceptor genes (*NRL* and *NR2E3*; **C)** across individual human foetal 15pcw pR2.1:GFP+ cells correlated to PC1. Each dot represents a cell and the cell position across PC1 (x-axis) is determined by its differential gene expression, which is indicated by colour (blue = naïve, yellow = mature). The expression level of each gene in each cell is shown on the y-axis.

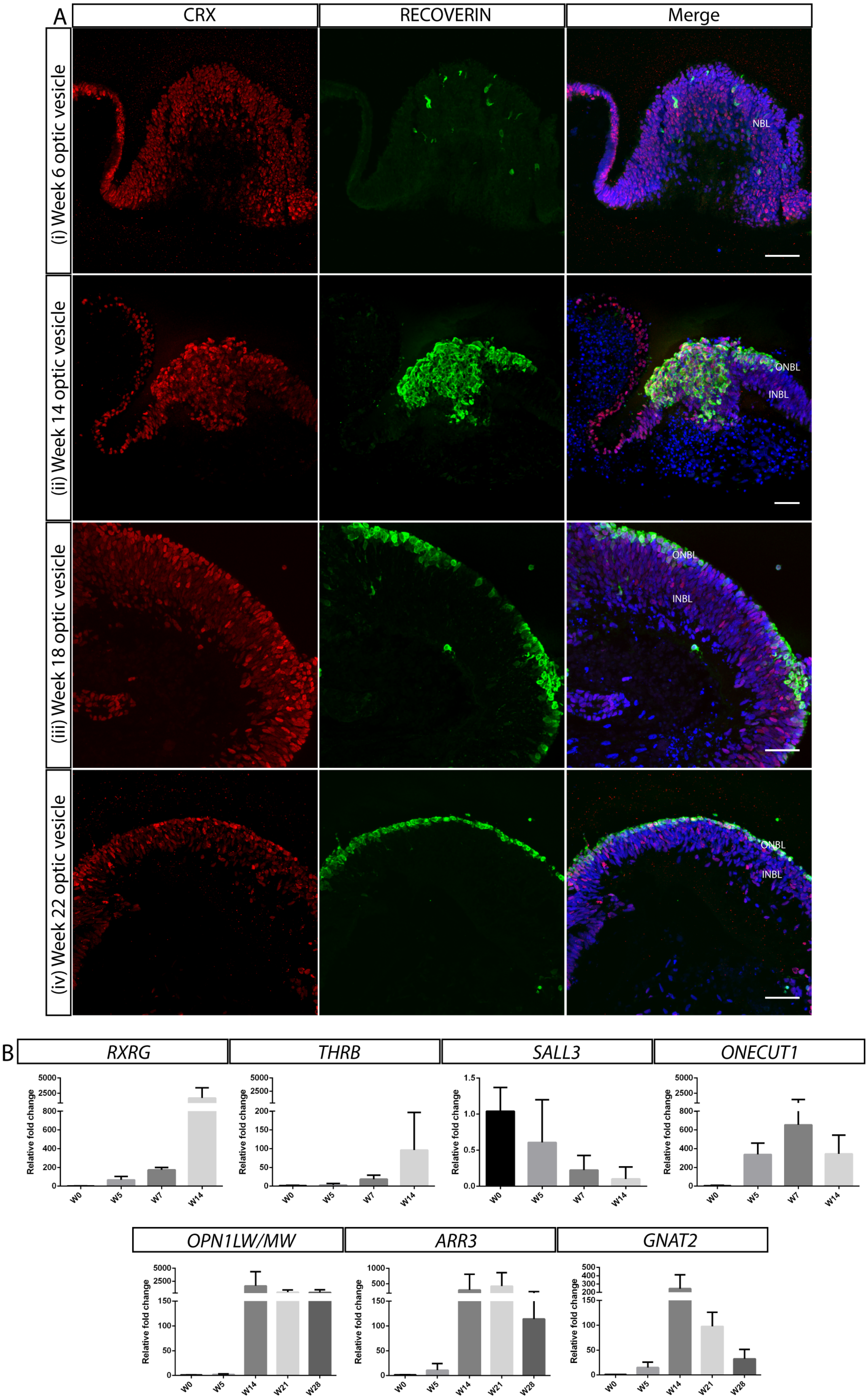
A
CRX
RECOVERIN
Merge
(i) Week 6 optic vesicle
(ii) Week 14 optic vesicle
(iii) Week 18 optic vesicle
(iv) Week 22 optic vesicle
NBL
ONBL
INBL
B
RXRG
THRB
SALL3
ONECUT1
OPN1LW/MW
ARR3
GNAT2
Relative fold change
W0
W5
W7
W14
W21
W28

Figure S5. Photoreceptor differentiation from iPSCs using 3D retinal organoid culture system (related to Figure 4)

(A i-iv) Protein expression of CRX and RECOVERIN was detected within iPSC-derived optic vesicle structures from week 6 through to week 22 of differentiation. **(B)** qRT PCR analysis of cone photoreceptor marker transcripts during retinal differentiation culture; n = 3 per timepoint. Error bars represent ± SD. Tissue section scalebar: 50µm. ONBL, outer neuroblastic layer; INBL, inner neuroblastic layer.

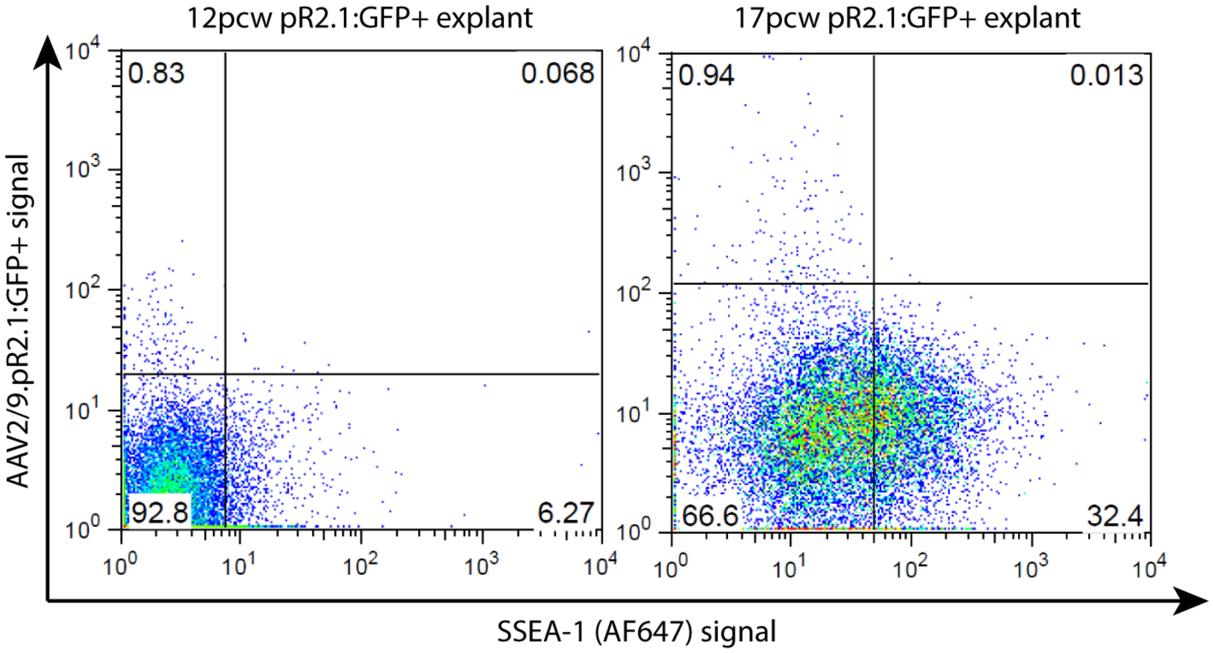
12pcw pR2.1:GFP+ explant
17pcw pR2.1:GFP+ explant
AAV2/9.pR2.1:GFP+ signal
SSEA-1 (AF647) signal
0.83
0.068
92.8
6.27
0.94
0.013
66.6
32.4

Figure S6. SSEA-1 expression in the human foetal retina (related to Figure 5)

Flow cytometry traces of pR2.1:GFP+ cells vs SSEA-1+ cells in the 12pcw and 17pcw foetal retina. Cells co-labelling for SSEA-1 and pR2.1:GFP+ cells were not detected at either timepoint in the foetal retinal samples, suggesting SSEA-1 is not expressed by pR2.1:GFP+ cone cells. In the 17pcw sample, SSEA-1 labelled 32.4% of GFP- cells.

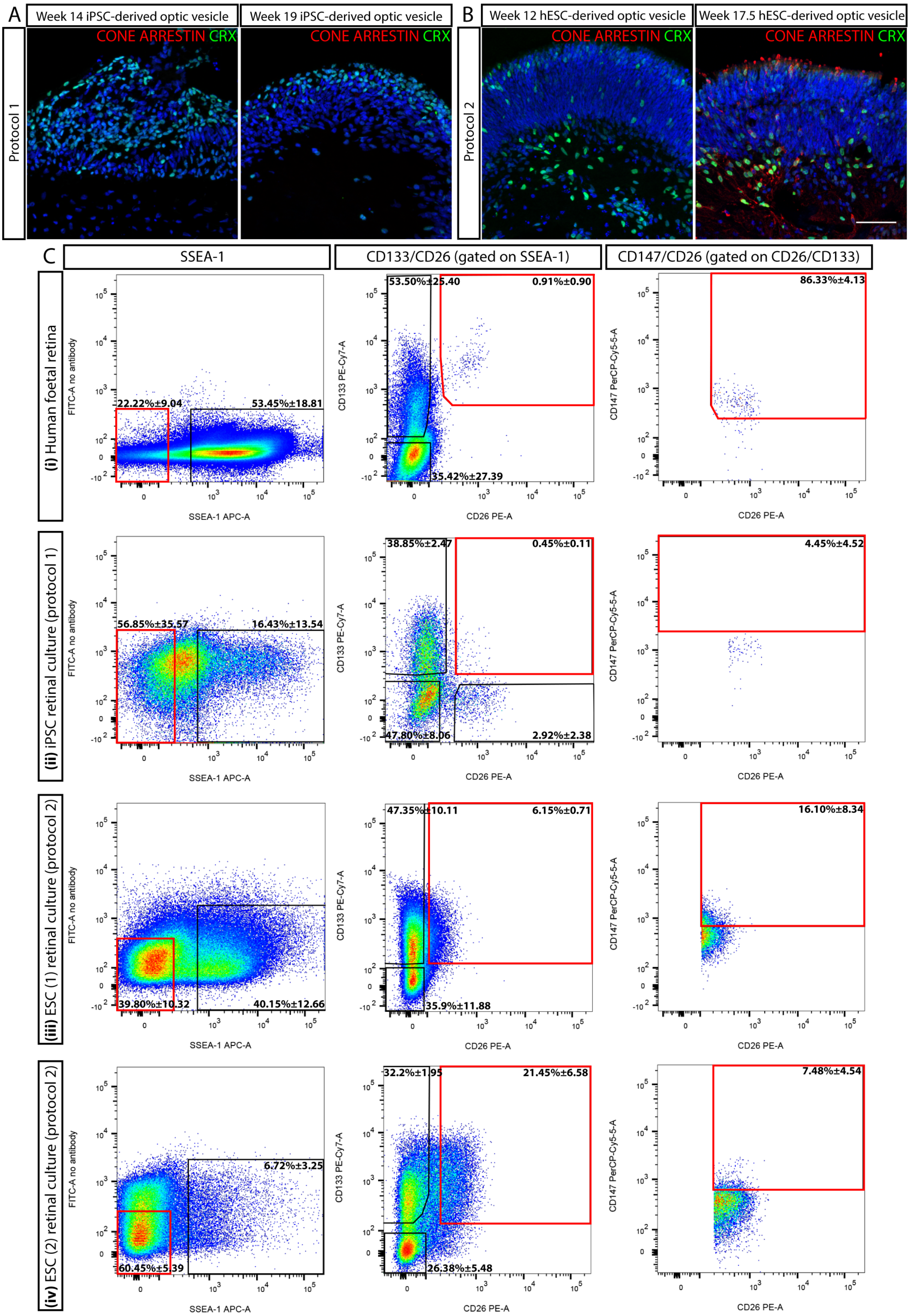
A
Week 14 iPSC-derived optic vesicle
Week 19 iPSC-derived optic vesicle
B
Week 12 hESC-derived optic vesicle
Week 17.5 hESC-derived optic vesicle
CONE ARRESTIN CRX
Protocol 1
Protocol 2
C
SSEA-1
CD133/CD26 (gated on SSEA-1)
CD147/CD26 (gated on CD26/CD133)
(i) Human foetal retina
22.22%±9.04
53.45%±18.81
53.50%±25.40
0.91%±0.90
35.42%±27.39
86.33%±4.13
(ii) iPSC retinal culture (protocol 1)
56.85%±35.57
16.43%±13.54
38.85%±2.47
0.45%±0.11
47.80%±8.06
2.92%±2.38
4.45%±4.52
(iii) ESC (1) retinal culture (protocol 2)
39.80%±10.32
40.15%±12.66
47.35%±10.11
6.15%±0.71
35.9%±11.88
16.10%±8.34
(iv) ESC (2) retinal culture (protocol 2)
60.45%±5.39
6.72%±3.25
32.2%±1.95
21.45%±6.58
26.38%±5.48
7.48%±4.54
FITC-A no antibody
SSEA-1 APC-A
CD133 PE-Cy7-A
CD26 PE-A
CD147 PerCP-Cy5-5-A

Figure S7. Cone differentiation and cone CD marker FAC-sorting from PSCs using retinal differentiation protocols (related to Figure 6)

(**A-B**) Histological sections of iPSC-derived optic vesicles generated using (**A**) the adapted Meyer et al., 2009 protocol (protocol 1) used for RNA seq analysis (refer to Figure 4) and (**B**) ESC-derived optic vesicles generated using the new protocol developed by Gonzalez-Cordero et al., 2017 (protocol 2; H9). Immunohistological analysis with CONE ARRESTIN/CRX antibodies detected CONE ARRESTIN by week 17.5 of differentiation within the ESC-derived vesicles **(B)**. Scalebar of retinal tissue section: 50µm.
(**C**) Representative flow cytometry traces showing the cell populations obtained when applying the cone CD marker combination (SSEA-1/CD133/CD26/CD147) onto **(C i)** the human foetal retina (example shows 17pcw foetal retina), **(C ii)** Week 20 iPSC-derived retinal differentiation cultures generated using protocol 1 (adapted Meyer et al., 2009 protocol), **(C iii)** Week 17-18 ESC (mShef10; ESC 1)-derived retinal differentiation cultures generated using protocol 2 (developed by Gonzalez-Cordero et al., 2017), **(C iv)** Week 17-18 ESC (H9; ESC 2)-derived retinal differentiation cultures generated using protocol 2. The foetal retina flow cytometry traces were used as a baseline reference for cone cell enrichment **(C i)**. Retinal differentiation of ESC lines with protocol 2 **(C iii and C iv)** showed SSEA1-CD133+CD26+CD147+ cell populations most similar to the foetal traces. Cell percentage is given within each gate; mean represents ±SD, n = 2-3.

SUPPLEMENTAL EXPERIMENTAL PROCEDURES

PSC maintenance and retinal differentiation

iPSCs were cultured on irradiated mouse embryonic fibroblast layer (GlobalStem) in knockout serum replacement medium (KSR; DMEM/F12 1:1 (Gibo), 20% Knockout Serum Replacement (KOSR; Life Technologies), 1% MEM non-essential amino acids (Life Technologies), 2mM L-glutamine (Life Technologies), 0.007% 2-mercaptoethanol (Life Technologies)) with 4ng/ml fibroblastic growth factor (FGF; Peprotech). H9 ESCs (Wicell) were cultured in feeder free conditions in E8 (Thermo Fisher) and geltrex coated 6 well plates. mShef10 ESCs (Centre for Stem Cell Biology, The University of Sheffield) were cultured in Nutristem (Geneflow) on Laminin-521 (BioLamina) coated 6 well plates. The retinal differentiation protocol for transcriptome profiling of iPSC-derived cones was based on Meyer et al., 2009 (Protocol 1). iPSC colonies were enzymatically lifted using 0.1% w/v dispase-DMEM/F12 (Life Technologies) on Day 0 and transfer into T25 flasks containing KSR medium without FGF to induce the formation of embryoid bodies (EBs). Medium was changed daily before substituting into neural induction medium (NIM) containing DMEM/F12 1:1 (Gibco), 1% MEM non-essential amino acids (Life Technologies), 1% N2 supplement (Gibco), 0.1% heparin (Sigma) on Day 4. To promote neural rosette formation, EBs were transferred onto 30% v/v laminin-DMEM/F12 (Sigma) coated plates in NIM on Day 6; neural rosettes appeared by differentiation day 8/9. Cultures were then fed every 2 days with NIM until Day 16, where the media was substituted for retinal differentiation media (RDM; 70% DMEM (Gibco), 30% F12 (Gibco) and 2% B27 supplement (Gibco)). Optic vesicle structure was visible from Day 18-20 onwards and media was changed every 2-3 days. 10% FBS (Gibco) was added to cultures at week 14 for late stage cultures. For CD marker FAC-sorting experiments, iPSCs were differentiated using protocol 1 (based on Meyer et al., 2009) and mShef10 and H9 lines were differentiated using the protocol as described in (Gonzalez-Cordero et al., 2017).

Human foetal retinal explant culture

Foetal retinae were isolated under sterile conditions in DMEM to remove surrounding ocular tissue using stainless steel 8mm blade curved micro scissors and 0.1mm straight forceps (World Precision Instruments). Intact foetal retina were cultured free floating in 12 or 24 well plates with retinal differentiation media (RDM) containing DMEM-F12 (Gibco), 1% Glutamax (Gibco), 1% N2 and 1% B27 neural supplements (Gibco), 10% FBS (Invitrogen) and 1.5% penicillin/streptomycin (Invitrogen). Cell culture media was changed every 2 days.

AAV2/9.pR2.1:GFP production and application

293T cells were cultured in D10 medium containing DMEM+Glutamax (Life Technologies), 10% FBS (Life Technologies) and 1% pencillin/streptomycin (Life Technologies) in 150cm plates (20 plates required for one batch of virus) and grown to 80% confluency. 100µg pd10/pR2.1.L/Mopsin-eGFP construct, 30µg pHGT1 helper vector, 100µg AAV2/9 caspid were added to 52.5ml DMEM and 1.2ml PEI polyethylenimine (PEI) prior to addition onto the 293T cells which facilitated transfection, before harvesting the cells after 72 hours using a cell scraper and collecting in TD buffer and performing four freeze (-80°C), thaw (37°C), vortex cycles for viral release. The pR2.1.L/M-opsin promoter was originally generated by the Jeremy Nathans group (Wang et al., 1992). Virus lysate underwent benzonase treatment (50 units; Sigma) and was prepared for purification through multiple centrifugation steps and sequential filtering with 5µm, 0.45µm and 0.22µm PES membranes. Virus was purified via ion exchange fast protein liquid chromatography using AKTA Prime (GE Healthcare) and anionic sephacryl S300 and POROS 50HQ columns prior to elution with an increasing salt gradient. The elution was concentrated using a Vivaspin 4 column (Sartorius) to a final volume ranging between 250-400µl. Viral genomic titer was determined through qRT PCR and the virus titer used in this study ranged from 1.48×10^{13}-1.01×10^{14}. Virus was added to foetal retinal explant and iPSC-derived retinal differentiation cultures at a MOI of 40,000-45,000.

Fluorescence-activated cell sorting (FACS) of human retinal cells

Human foetal and PSC-derived retinal tissue were enzymatically dissociated into single cells using the papain dissociation system (Worthington Biochemical, Lorne Laboratories, UK) according to manufacturer's protocol. All samples were passed through 70µm cell strainer prior to FACS. For FAC-sorting of pR2.1.GFP+ and GFP- retinal material, cells were resuspended in blocking solution (BS; 1% (w/v) BSA/PBS) with the addition of DAPI (2µl; Sigma) before sorting to allow the determination of live cell population. For cone CD marker sorting, cells were counted using a haemocytometer and resuspended in BS at a concentration of 1×10^6 cells per 100ul. After incubating cell for 1 hour on ice, conjugated antibodies (APC-conjugated SSEA-1, clone REA321, Miltenyi Biotec, Cat no. 130-104-936; PE- conjugated CD26, clone M-A261, BD Biosciences, Cat no. 555437; PE- Vio770-conjugated CD133, clone 293C3, Miltenyi Biotec, Cat no. 130-104-117; PerCP-Cy5.5-conjugated CD147, clone HIM6, BD Biosciences, Cat no. 562554) or isotype controls were added to cells using the manufacturer's recommendations and incubated for a further hour on ice in the dark. Cells were washed in PBS and resuspended in BS for sorting. The BD FACSARIA III was used to isolate cells which were collected into

50% (v/v) FBS/DMEM media. FITC-A vs PerCP-Cy5-5A channels were used to detect GFP+ signal, whereas the according channels matching the fluorophores of the conjugated CD marker antibodies were used to detect positive signal. Isotype and unstained controls were used to set gates and apply necessary compensation. Post-sorting, cells were centrifuged at 300 x g for 15 minutes at 4°C; cell pellets for RNA extraction were immediately snap frozen on dry ice, whereas cells for immunocytochemistry were plated out on to pre-coated poly-L-lysine (Sigma) and laminin (1:30; Sigma) chamber slides. For FACS analysis of foetal and ESC-derived retinal samples, 4 independent samples were analysed for each CD marker combination and at least 50 cells were counted for each FAC-sorted cell population. FlowJo flow cytometry software was used for FACS trace visualisation and analysis.

Immunocytochemistry on dissociated retinal cells

Dissociated retinal cells on chamber slides were fixed in 4% (w/v) PFA/PBS for 5 minutes at 37°C prior to a further 15 minutes incubation in 2% (w/v) PFA/ 30% (w/v) sucrose/PBS at room temperature (RT). Cells were washed three times in PBS and incubated for 1 hour at RT in BS (10% (v/v) foetal bovine serum (FBS)/PBS (Gibo), 1% (w/v) bovine serum albumin/PBS) with 0.1% (v/v) Triton X-100. Primary antibodies (L/M-opsin, Millipore, Cat no. AB5405, 1:400, CRX, Abnova, H00001406-M02, 1:800; Cone arrestin, NovusBio, NBP1-19629, 1:100) were added on to cells and incubated for 1 hour at RT. Cells were washed with three times in PBS and incubated for a further hour with secondary antibodies (Alexa Fluor 594 Goat anti-rabbit IgG (Cat no. A11032) or Alexa Fluro 488 Goat anti-mouse IgG (Cat no. A1080), Invitrogen, 1:800). The same procedure was conducted for CD marker immunocytochemistry (L/M-opsin, Millipore, Cat no. AB5405, 1:400, Cone arrestin, NovusBio, NBP1-19629, 1:100, PE- conjugated CD26, 1:500, BD Biosciences, Cat no. 555437; PE- Vio770-conjugated CD133, 1:500, Miltenyi Biotec, Cat no. 130-104-117; PerCP-Cy5.5-conjugated CD147, clone 1:500, BD Biosciences, Cat no. 562554) on dissociated cells, with the removal of Triton X-100 from BS. DAPI incubation and mounting procedure was performed as described in histology and immunohistochemistry section.

Human BD lyoplate screening panel protocol

Retinal samples previously transfected with AAV2/9.pR2.1:GFP reporter were dissociated using the papain method as described in the FACS methods section and the protocol for the human BD Lyoplate^{TM} Screening Panels was followed accordingly to manufacturer's instructions. Briefly, cells were resuspended in BD Pharmingen Stain Buffer with 5mM EDTA to a cell concentration of 25k-1x10^6 cells/100µl and aliquoted into

3x BD Falcon™ round bottom 96 well plates (100µl per well). Primary antibodies of the BD Lyoplate™ Screening Panels were reconstituted in 1x PBS (110µl per well) and 20µl of each antibody was then added to the cells and incubated for 30 minutes on ice. Primary antibody was omitted for negative control and wells were allocated for IgG/IgM positive controls. Cells were then washed in stain buffer, resuspended in 100µl of secondary antibody (Alexa Fluor 647) solution before incubating on ice for 30 minutes in the dark. Subsequently, cells were washed and centrifuged before being resuspended in 150µl of BD Pharmingen Stain Buffer + EDTA. Samples were then analysed using the BD FACSCabilur (BD Biosciences). 15,000-20,000 events were collected per well and results were analysed using the FlowJo software.

Histology and Immunohistochemistry

Human foetal eyes were fixed in 4% (w/v) PFA/PBS overnight at 4°C; retinal explants/wholemounts and PSC-derived retinal differentiation samples were fixed for 30 minutes at 4°C. For embedding, samples were washed three times in PBS and equilibrated in 30% (w/v) sucrose/PBS solution for cryo-protection at RT; once sunk, samples were then orientated and placed into foil moulds containing optimal cutting temperature (OCT) compound, before freezing in methylbutane-dry ice slurry. Tissue sections were cut to 14-16µm thickness using the Leica CM1900 UV cryostat and collected on SuperfrostTM plus glass slides (VWR). For immunohistochemistry, retinal sections were washed in PBS for 13 minutes at 37°C to remove the OCT compound (step excluded for wholemount staining) and incubated in BS (10% (v/v) foetal bovine serum (FBS)/PBS (Gibo), 1% (w/v) bovine serum albumin/PBS) with 0.1% (v/v) Triton X-100 for 1 hour at RT. Sections were subsequently incubated with primary antibody for 1 hour at RT or overnight at 4°C. Primary antibodies used in this study include Recoverin, Millipore, Cat no. AB5585, 1:1000; CRX, Abnova, Cat no. H00001406-M02, 1:800; L/M-opsin, Millipore, Cat no. AB5405, 1:500; S-opsin, Millipore, Cat No. AB5407, 1:500; Rxrg, Abcam, Cat no. AB15518, 1:300; Gnat2, Santa Cruz, Cat no. sc-390, 1:300; Ki67, Abcam, Cat no. AB15580, 1:300; Nr2e3, R&D Systems, Cat no. 2ZH7223H, 1:500; Rhodopsin; Upstate, Cat no. 06-770 1:1000; Nrl, R&D Systems, Cat no. AF2945, 1:500, Cone arrestin, NovusBio, Cat no. NBP1-19629 1:100. Primary antibody was omitted for negative controls. Sections were washed three times in 1x PBS before applying the secondary antibody for 1 hour at RT (AlexaFluor 594 Goat anti-rabbit (Cat no. A11037), AlexaFluor 488 Donkey anti-mouse (Cat no. A-21202) , AlexFluor 594 Donkey anti-goat (Cat no A-11058). Invitrogen, 1:800). Sections were washed 3 times with PBS prior to DAPI incubation (1:3000 in PBS) at RT for

5 minutes, allowing for the visualisation of cell nuclei. Sections were washed again in PBS, before applying with Citifluor AF-1 mounting medium, 1.5 coverslips and clear nail polish to seal coverslip.

Microscopy and image processing

Z-28 projection images of retinal sections and wholemount immunostainings were acquired using the Zeiss LSM710 (Zen2009, Zeiss) confocal microscope. Z-28 projection and at least 2x2 tile scan images were acquired for of FAC-sorted cells to be counted. Brightfield and fluorescent images of foetal pR2.1:GFP+ retinal explants were captured using the Leica MZFLIII fluorescence stereomicroscope and the Leica DC500 camera. Fluorescent and brightfield images of stem cell-derived cones were acquired using the inverted Olympus IX71 (Carl Zeiss, Jena, Germany) microscope with a Hamamatsu ORCA-ER digital camera (Hamamatsu Corp., Bridgewater, NJ). Images were processed using Zen2009 (Zeiss), ImageJ and Illustrator CS6 (Adobe). Cell counting was completed using ImageJ software.

RNA isolation and quantitative real-time PCR

Retinal tissue was processed with the mirVana RNA extraction kit (Ambion) according to manufacturer's recommendation. For qRT PCR analysis, RNA samples were treated with DNaseI (Invitrogen) and cDNA was synthesised using SuperScript III Reverse Transcriptase (Invitrogen). cDNA samples were diluted to the same concentration (5ng) with DEPC-treated water. RT qPCR procedure was carried out in 96 well plates with a total reaction volume of 20µl per well (2µl of cDNA sample, 17µl of TaqMan Gene Expression Master Mix (Thermo Fisher) with DEPC-treated water and 1µl of TaqMan gene expression assays (Thermo Fisher). The following gene expression assays were used in this study for detecting transcripts of interest: *RXRG* (Hs00199455_m1), *ONECUT1* (Hs00413554_m1), *THRB2* (AJ20TM1), *SALL3* (Hs00923915_m1), *GNAT2* (Hs00292542_m1), *ARR3* (Hs00182888_m1), *OPN1SW* (Hs00181790_m1), *OPN1LW/MW* (Hs00241039_m1), *RCVRN* (Hs00610056_m1), *CRX* (Hs00230899_m1). The assay used for *OPN1LW/MW* detects transcripts from all L and M-opsin genes. *GADPH* (Hs02758991_g1) was used as an internal control to normalise the expression of candidate genes and sample cDNA was replaced with DEPC-treated water for negative controls. qRT PCRs were performed on a 7500 Real-Time PCR System with the FAM settings according to manufacturer's recommendation. The following PCR cycle was used for all experiments: Stage 1, 1x cycle, 50°C for 2 minutes; Stage 2, 1x cycle, 95°C for 10 minutes; Stage 3, 40x cycles, 95°C for 15 seconds, 60°C for 60 seconds. The

Applied Biosystems 7500 Real Time PCR System software (v2.0.2) was used to assess quality of sample output before exporting and performing data analysis in Microsoft Excel.

Bulk mRNA sequencing and bioinformatics analysis

RNA concentration and quality was assessed on the Agilent Bioanalyzer or Tapestation using a RNA 6000 Pico kit (Agilent Technologies). High quality RNA (RIN:6.70-10) was used for RNA seq. cDNA quality was assessed using Qubit dsDNA HS Assay kit and Qubit 2.0 Fluorometer (ThermoFisher). pR2.1.GFP+ and GFP- FAC-sorted samples for bulk RNA-seq analysis were amplified using the SMART-Seq v4 Ultra Low Input RNA kit (Clontech Laboratories) and cDNA libraries were prepared using the Nextera XT DNA Library Preparation Kit (Illumina), before sequencing libraries using the Illumina NextSeq500 system with a targeted sequencing depth of 17million 43bp paired end per sample. FASTQ files containing raw RNA seq data were aligned using Illumina RNA Seq STAR alignment tool (version 1.1.0) to the reference human genome (GRCh37/hg19), in order to generate BAM files. Quality control was performed pre and post alignment using the RNA-Seq alignment tool in BaseSpace (Illumina). BAM files and a gene annotation file (Homo_sapiens.GRCh37.87.chr.gtf from Ensembl) were uploaded to Galaxy (version 17.01). Aligned reads were assigned to genes and quantified using featureCounts (version 1.4.6.p5 (Liao et al., 2014) to obtain a raw count matrix for each sample. The raw count matrices were loaded into Strand NGS (Strand Genomics), before performing DESeq normalisation for hierarchical clustering analysis (Euclidean similarity measure and Ward's linkage rule used), and data visualisation using Principal Component Analysis (PCA) and making heatmap representations. The raw count matrices were then used as input to the DESeq2 (Love et al., 2014) pipeline using default parameters with the Galaxy version 2.11.38 for the differential gene expression analyses, which uses Wald test statistics and Benjamini-Hochberg correction for multiple testing. log10 adjusted p-values and log2 fold changes generated by these analyses were plotted using R (version 3.2.1 (2015-06-18) to create volcano plots. Genes with total raw count matrices lower than 10 across all samples were removed from analysis. Venn diagrams were constructed using Bioinformatic & Evolutionary Genomics tool (http://bioinformatics.psb.ugent.be/webtools/Venn/). GO analysis was performed using Enrichr (Chen et al., 2013, Kuleshov et al., 2016) and enriched GO terms with a combined score >10 were represented using Revigo (Supek et al., 2011). Accession numbers E-MTAB-6057 can be used to access fastq files of all bulk RNA seq samples on ArrayExpress EMBL-EBI.

Single cell RNA sequencing and bioinformatics analysis

For single cell analysis, pR2.1.GFP+ were isolated via FACS and separated using a medium-sized (10-17µm cell diameter) integrated fluidic circuit (IFC; Fluidigm), using the Fluidigm C1 system. Prior to loading on the IFC, cell viability was assessed using a standard trypan blue exclusion test before resuspending cells at a concentration of 667cells/µl. 3µl total sample volume was loaded onto IFC and cell were imaged via phase-contrast microscopy to check for the single cell status of each chamber. 74 single cells were captured for the experiment and chambers containing debris, dead cells and multiple cells were excluded from the analysis. After collecting cell harvest from the IFC, amplification, cDNA libraries and sequencing was performed using the same bulk RNA seq procedures. A targeted sequencing depth of 2 million 75bp paired end reads per sample was used and External RNA Control Consortium (ERCC) RNA spike-in Mix (ThermoFisher) was added to each cell sample prior to sequencing. After the sequencing, FASTQ files from individual cell samples were aligned to the hg19 build of the human genome with ERCC spike-in sequences (Thermofisher) using subread version 1.5.0-p1 (Liao et al 2013). The featureCounts software (Liao et al 2014) was used to obtain a matrix of gene by cell counts using gene feature files from Ensembl GRCh37 version 82 and ThermoFisher. The count matrix was then analyzed as described by (Lun et al., 2016b). Low quality cells were defined from several quality control metrics, including the log-library sizes and log-number of expressed features (where small outlier values were removed), and the proportion of reads mapped to the ERCC spike-ins or mitochondrial genome (where large outliers were removed). This led to the inclusion of 65 cells for the downstream analysis. Outlier values were defined as three median absolute deviations above or below the median. Low-abundance genes were defined as those with average counts below 1 and were filtered out, leaving 19,650 for downstream analysis. Normalization was performed for endogenous genes by computing size factors with the deconvolution method (Lun et al., 2016a). For spike-in transcripts, size factors were computed from the sum of spike-in counts in each cell. In both cases, the size factors were used to obtain normalized log-expression values for all genes/transcripts in all cells. To identify highly variable genes (HVGs), a trend was fitted to the variance of the log-expression values against the mean for the spike-ins. This was used to decompose the variance for endogenous genes into biological and technical components. HVGs were defined as those genes where the total variance was significantly higher than the technical trend (FDR<0.05) and the biological component was at least 0.5. The log-expression values for the HVGs across all cells were then used in a principal components analysis. Genes correlated with PC1 were identified using limma (Ritchie et al., 2015) as previously described. The observation that PC1 corresponds to cell maturation arose from unbiased analysis and was not fitted to

match some criteria. Accession numbers E-MTAB-6058 can be used to access fastq files single cell RNA seq samples on ArrayExpress EMBL-EBI.

CD marker bioinformatics

The cell surface marker protein query database contains predicted protein features based on human protein sequences from release 2013_04 of the UniProtKB/Swiss-Prot database, which can be used to identify putative cell surface markers within a list of genes. To create the database, proteins with sequence length > 1,500 amino acids were excluded as they were not compatible with some of the programs used for the feature predictions. A representative set of proteins with less than 90% pair-wise sequence identity was subsequently identified using the CD-HIT program (Li and Godzik, 2006). This resulted in a set of 19281 proteins, for which predictions were obtained using the following programs: MEMSAT-SVM (transmembrane segment prediction (Nugent and Jones, 2009), PredGPI (GPI anchor prediction, (Pierleoni et al., 2008), the specific Support Vector Machine trained for GO term GO:0005886 'plasma membrane' within FFPred2 (version 2.0, (Minneci et al., 2013), and DISOPRED2 (protein disorder prediction,(Ward et al., 2004). The predictions were used to label all proteins in the database as either "positive" when it was predicted to be a putative cell surface marker, or "negative" otherwise. Criteria for assigning a putative cell surface marker required two criteria: i) at least 16 transmembrane helices predicted by MEMSAT-SVM *or* a PredGPI prediction of "Highly Probable", and ii) a "plasma membrane" FFPred2 prediction with confidence ≥ 0.8. Applying these rules to the 19281 proteins present in the database, 3367 were labelled as "positive" and 15914 as "negative". A benchmarking test was performed against a list of UniProt Identifiers for 206 known human cluster of differentiation cell surface (CD) markers to evaluate the performance of the prediction pipeline, which showed 85% sensitivity (168/206 marked as positive).

SUPPLEMENTAL REFERENCES

CHEN, E. Y., TAN, C. M., KOU, Y., DUAN, Q., WANG, Z., MEIRELLES, G. V., CLARK, N. R. & MA'AYAN, A. 2013. Enrichr: interactive and collaborative HTML5 gene list enrichment analysis tool. *BMC Bioinformatics,* 14**,** 128.

GONZALEZ-CORDERO, A., KRUCZEK, K., NAEEM, A., FERNANDO, M., KLOC, M., RIBEIRO, J., GOH, D., DURAN, Y., BLACKFORD, S. J. I., ABELLEIRA-HERVAS, L., SAMPSON, R. D., SHUM, I. O., BRANCH, M. J., GARDNER, P. J., SOWDEN, J. C., BAINBRIDGE, J. W. B., SMITH, A. J., WEST, E. L., PEARSON, R. A. & ALI, R. R. 2017. Recapitulation of Human Retinal Development from Human Pluripotent Stem Cells Generates Transplantable Populations of Cone Photoreceptors. *Stem Cell Reports*.

KULESHOV, M. V., JONES, M. R., ROUILLARD, A. D., FERNANDEZ, N. F., DUAN, Q., WANG, Z., KOPLEV, S., JENKINS, S. L., JAGODNIK, K. M., LACHMANN, A., MCDERMOTT, M. G.,

MONTEIRO, C. D., GUNDERSEN, G. W. & MA'AYAN, A. 2016. Enrichr: a comprehensive gene set enrichment analysis web server 2016 update. *Nucleic Acids Res,* 44**,** W90-7.

LI, W. & GODZIK, A. 2006. Cd-hit: a fast program for clustering and comparing large sets of protein or nucleotide sequences. *Bioinformatics,* 22**,** 1658-9.

LIAO, Y., SMYTH, G. K. & SHI, W. 2014. featureCounts: an efficient general purpose program for assigning sequence reads to genomic features. *Bioinformatics,* 30**,** 923-30.

LOVE, M. I., HUBER, W. & ANDERS, S. 2014. Moderated estimation of fold change and dispersion for RNA-seq data with DESeq2. *Genome Biol,* 15**,** 550.

LUN, A. T., BACH, K. & MARIONI, J. C. 2016a. Pooling across cells to normalize single-cell RNA sequencing data with many zero counts. *Genome Biol,* 17**,** 75.

LUN, A. T., MCCARTHY, D. J. & MARIONI, J. C. 2016b. A step-by-step workflow for low-level analysis of single-cell RNA-seq data with Bioconductor. *F1000Res,* 5**,** 2122.

MINNECI, F., PIOVESAN, D., COZZETTO, D. & JONES, D. T. 2013. FFPred 2.0: improved homology-independent prediction of gene ontology terms for eukaryotic protein sequences. *PLoS One,* 8**,** e63754.

NUGENT, T. & JONES, D. T. 2009. Transmembrane protein topology prediction using support vector machines. *BMC Bioinformatics,* 10**,** 159.

PIERLEONI, A., MARTELLI, P. L. & CASADIO, R. 2008. PredGPI: a GPI-anchor predictor. *BMC Bioinformatics,* 9**,** 392.

RITCHIE, M. E., PHIPSON, B., WU, D., HU, Y., LAW, C. W., SHI, W. & SMYTH, G. K. 2015. limma powers differential expression analyses for RNA-sequencing and microarray studies. *Nucleic Acids Res,* 43**,** e47.

SUPEK, F., BOSNJAK, M., SKUNCA, N. & SMUC, T. 2011. REVIGO summarizes and visualizes long lists of gene ontology terms. *PLoS One,* 6**,** e21800.

WANG, Y., MACKE, J. P., MERBS, S. L., ZACK, D. J., KLAUNBERG, B., BENNETT, J., GEARHART, J. & NATHANS, J. 1992. A locus control region adjacent to the human red and green visual pigment genes. *Neuron,* 9**,** 429-40.

WARD, J. J., SODHI, J. S., MCGUFFIN, L. J., BUXTON, B. F. & JONES, D. T. 2004. Prediction and functional analysis of native disorder in proteins from the three kingdoms of life. *J Mol Biol,* 337**,** 635-45.
