# Supplementary material for: Isolation and Comparative Transcriptome Analysis of Human Fetal and iPSC-Derived Cone Photoreceptor Cells
Source: Stem Cell Reports. 2017 Nov 16;9(6):1898–915. doi: 10.1016/j.stemcr.2017.10.018 (PMC5785701; doi:10.1016/j.stemcr.2017.10.018)
Supplement: Document S3. Article plus Supplemental Information [file mmc3.pdf]

Stem Cell Reports
Article
ISSCR
OPEN ACCESS

Isolation and Comparative Transcriptome Analysis of Human Fetal and iPSC-Derived Cone Photoreceptor Cells

Emily Welby,[1,2] Jorn Lakowski,[1,2] Valentina Di Foggia,[1,2] Dimitri Budinger,[1,2] Anai Gonzalez-Cordero,[3] Aaron T.L. Lun,[4] Michael Epstein,[4] Aara Patel,[1,2] Elisa Cuevas,[1,2] Kamil Kruczek,[3] Arifa Naeem,[3] Federico Minneci,[5] Mike Hubank,[6] David T. Jones,[5] John C. Marioni,[4,7] Robin R. Ali,[3] and Jane C. Sowden[1,2,*]

[1]Stem Cells and Regenerative Medicine Section
[2]NIHR Great Ormond Street Hospital Biomedical Research Centre
UCL Great Ormond Street Institute of Child Health, 30 Guilford Street, London WC1N 1EH, UK
[3]Department of Genetics, UCL Institute of Ophthalmology, 11-43 Bath Street, London EC1V 9EL, UK
[4]Cancer Research UK Cambridge Institute, University of Cambridge, Li Ka Shing Centre, Robinson Way, Cambridge CB2 0RE, UK
[5]Department of Computer Science, University College London, Gower Street, London WC1E 6BT, UK
[6]UCL Genomics, UCL Great Ormond Street Institute of Child Health, 30 Guilford Street, London WC1N 1EH, UK
[7]EMBL-European Bioinformatics Institute, Wellcome Genome Campus, Hinxton, Cambridge CB10 1SD, UK
*Correspondence: j.sowden@ucl.ac.uk
https://doi.org/10.1016/j.stemcr.2017.10.018

SUMMARY

Loss of cone photoreceptors, crucial for daylight vision, has the greatest impact on sight in retinal degeneration. Transplantation of stem cell-derived L/M-opsin cones, which form 90% of the human cone population, could provide a feasible therapy to restore vision. However, transcriptomic similarities between fetal and stem cell-derived cones remain to be defined, in addition to development of cone cell purification strategies. Here, we report an analysis of the human L/M-opsin cone photoreceptor transcriptome using an AAV2/9.pR2.1:GFP reporter. This led to the identification of a cone-enriched gene signature, which we used to demonstrate similar gene expression between fetal and stem cell-derived cones. We then defined a cluster of differentiation marker combination that, when used for cell sorting, significantly enriches for cone photoreceptors from the fetal retina and stem cell-derived retinal organoids, respectively. These data may facilitate more efficient isolation of human stem cell-derived cones for use in clinical transplantation studies.

INTRODUCTION

Retinopathies featuring the progressive degeneration of the rod and cone photoreceptor cells lead to permanent blindness. Inherited retinal dystrophies affect 1 in 3,000 people worldwide (Bessant et al., 2001), with the most common form, retinitis pigmentosa, presenting with primary rod degeneration followed by the loss of cones. Age-related macular degeneration results in the primary loss of cones in the macula. Cone degeneration has the greatest impact on sight since cones are fundamental for the detection of color, daylight vision, and high visual acuity. The human retina contains three different types of cone photoreceptors; approximately 5%–10% of the cone population express S-opsin, while the majority of cones (90%–95%) express either L-opsin or M-opsin light-sensitive proteins (Craft et al., 2014). Even though cone photoreceptors are a rare population, forming 2%–4% of total retinal cells, humans are dependent on these cells for optimal vision.

There is a paucity of effective treatments for retinal degeneration. Cell-replacement therapy offers an approach for advanced disease with extensive cone photoreceptor loss. As inner retinal neurons are initially preserved within the diseased retina, photoreceptor cell replacement might provide a feasible approach following proof-of-concept studies in the mouse (MacLaren et al., 2006; Bartsch et al., 2008; Santos-Ferreira et al., 2016b), which showed improvements in visual function following transplantation (Pearson et al., 2012; Lamba et al., 2009). However, recent studies suggest that functional recovery may be achieved by transplanted photoreceptor cells predominantly transferring genetic material, rather than making new connections with the host retina (Pearson et al., 2016; Santos-Ferreira et al., 2016a; Decembrini et al., 2017), and thus developing ways to improve functional connectivity of transplanted cells remains a priority. Combining induced pluripotent stem cell (iPSC) generation (Takahashi et al., 2007) and retinal organoid culture systems (Meyer et al., 2009; Nakano et al., 2012; Zhong et al., 2014; Reichman et al., 2014) has allowed recapitulation of human retinogenesis and the generation of photoreceptors from human somatic cell sources which could provide a renewable source of autologous cells for cone transplantation therapy. Nevertheless, there has yet to be a direct comparative study of the transcriptome of human fetal and PSC-derived cone photoreceptors to assess their degree of similarity.

In addition to providing baseline data for assessing suitability of iPSC-derived cones for therapeutic applications, establishing the transcriptome of human developing L/M-opsin cones would allow exploration of the molecular determinants needed for early specification and maintenance of the cone cell fate, which remains to be fully elucidated. In the human retina, from as early as fetal week 10.9,

This is an open access article under the CC BY license (http://creativecommons.org/licenses/by/4.0/).
CrossMark

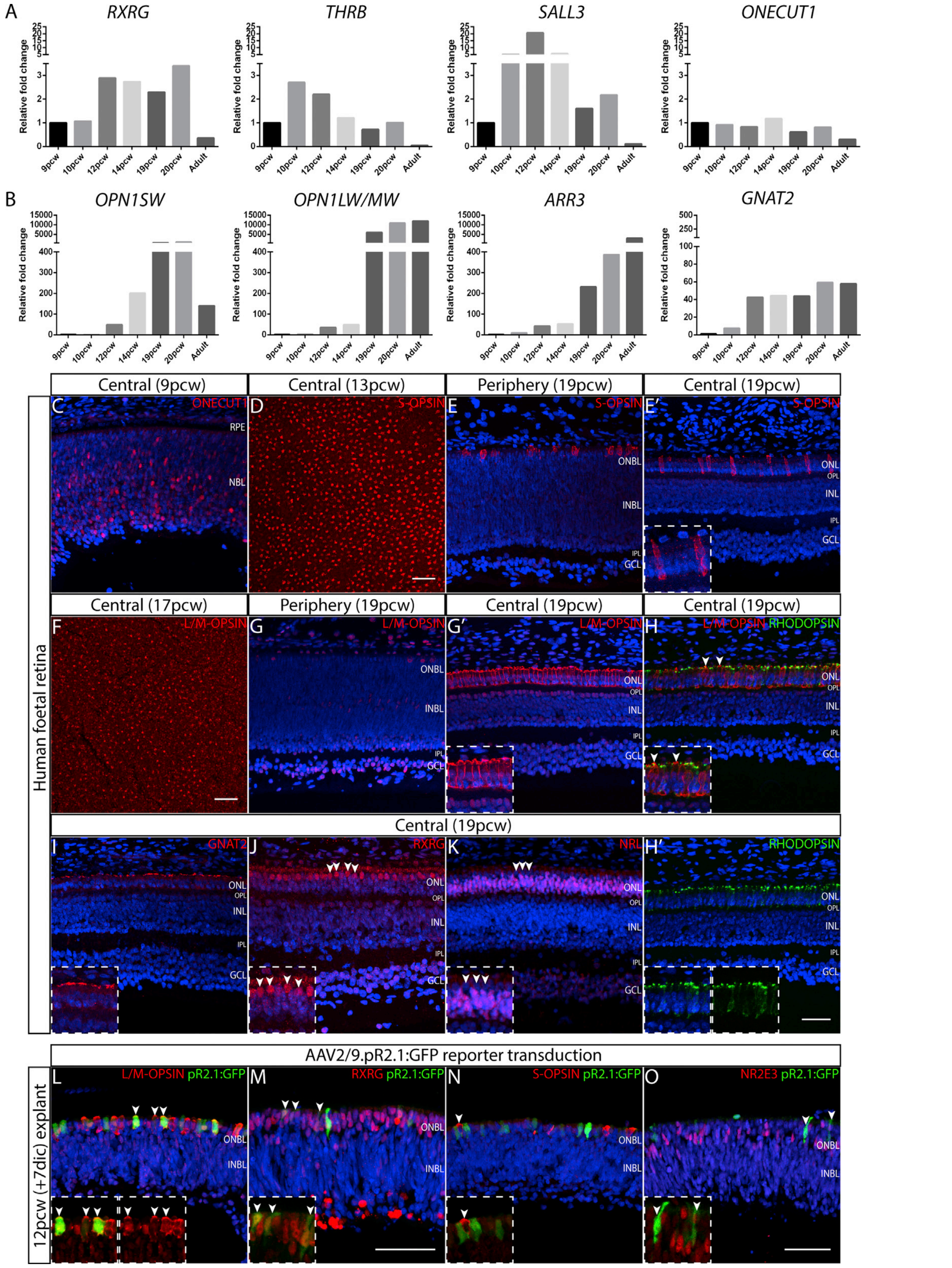

(legend on next page)

cones and rods can be distinguished based on cell morphology, nuclei condensation, and proximity to the central retina where the fovea forms (Xiao and Hendrickson, 2000; Hendrickson et al., 2008). To date, the most well-documented human cone-specific markers are the cone opsins (Xiao and Hendrickson, 2000; Cornish et al., 2004), with S-cones appearing first, followed by L/M-cones. L- and M-opsin-expressing photoreceptors can be distinguished via high-resolution retinal imaging and retinal densitometry (Roorda and Williams, 1999; Hofer et al., 2005), but cannot be separated via immunohistochemistry due to the high homology (98%) of the *OPN1LW* and *OPN1MW* genes (Nathans et al., 1986; Cornish et al., 2004). Several studies in the mouse, suggest that rod and cone photoreceptors share a common cell lineage and define S-cones as a default cell fate pathway, which is driven through the interaction of the cone-rod homeobox gene (*Crx*) and the nuclear receptor RAR-related orphan receptors (*Rora/Rorb*) (Fujieda et al., 2009; Srinivas et al., 2006; Kim et al., 2016). To become M-cones, precursor cells must express transcription factor genes, including retinoid-x-receptor gamma (*Rxrg*) and thyroid hormone receptor beta 2 (*Thrb2*), which regulate cone cell identity (Ng et al., 2001; Roberts et al., 2005). Other lines of evidence define the Onecut homeobox 1 (*ONECUT1*) and spalt-like transcription factor (*Sall3*) genes, as necessary for generating progenitors biased to produce cone and horizontal cells (Emerson et al., 2013; de Melo et al., 2011; Suzuki et al., 2013). Moreover, in the human retina the characterization of cone-associated regulatory factors remains to be explored. Cone transcriptome data only exist for adult mouse cone photoreceptors (Siegert et al., 2009); in human, only total retina (Hornan et al., 2007; Cai et al., 2012; Bowes Rickman et al., 2006; Whitmore et al., 2014; Li et al., 2014) and isolated Crx.GFP-expressing embryonic stem cell (ESC)-derived cells (Kaewkhaw et al., 2015) have been analyzed. An equivalent dataset for human fetal cone photoreceptors is currently lacking. Furthermore, single-cell RNA sequencing (RNA-seq) (Lun et al., 2016) offers a novel approach to explore the heterogeneity of human cone cell populations.

The enrichment of cone photoreceptors without genetic manipulation (e.g., GFP reporter gene labeling) will be crucial prior to transplantation into human patients. We, and others, have previously described the enrichment of mouse rod photoreceptor precursor cells by targeting antigens expressed on the outer cell membrane, with corresponding antibodies conjugated to fluorophores in cell-sorting strategies (Eberle et al., 2011, 2014; Lakowski et al., 2011; Eberle et al., 2014). An extended cell surface biomarker panel was developed to enrich rod photoreceptors from mouse ESC-derived retinal cultures prior to transplantation (Lakowski et al., 2015). However, little progress has been made toward defining cell surface markers profiles or enrichment approaches for cone photoreceptors.

In this study, we used an adeno-associated virus (AAV) GFP reporter, driven by a human L/M-opsin gene promoter (pR2.1:GFP), to specifically label and isolate human fetal cones and an equivalent population from iPSC-derived retinal differentiation cultures. We report transcriptome analysis of developing L/M-opsin cone photoreceptor populations and single cells, identifying genes that are upregulated in early and late L/M-opsin cones. A direct comparative analysis of fetal and iPSC-derived cells demonstrated similar cone gene expression. From these transcriptome data and using fluorescence-activated cell-sorting (FACS) analysis, we established the cell surface marker expression profile of L/M-opsin cone photoreceptors, which led to the identification a cone biomarker panel (SSEA1−CD26+CD133+CD147+) that positively enriches for fetal L/M-opsin cones and a stem cell-derived cone photoreceptor population.

RESULTS

Cone Photoreceptor Marker Expression in the Developing Human Fetal Retina

We first assessed the developmental expression profile of a set of cone photoreceptor markers in a series of fetal (9–20 post conception weeks [pcw]) and adult retinal samples by qRT-PCR (Figure 1). Transcription factor genes implicated

Figure 1. Analysis of Cone Photoreceptor Markers in the Developing Human Retina
qRT-PCR analysis of early cone photoreceptor genes, *RXRG*, *THRB*, *SALL3*, and *ONECUT1* (A), and mature cone photoreceptor genes *OPN1SW*, *OPN1LW/MW*, *ARR3*, and *GNAT2* (B), in fetal (9–20 pcw) and adult retinal samples (n = 1 per time point). Immunohistochemistry analysis of cone photoreceptor markers, ONECUT1 (C), S-OPSIN (D–E′), L/M-OPSIN (F–H), GNAT2 (I), and RXRG (J), and rod photoreceptor markers, NRL (K) and RHODOPSIN (H and H′), within 9–19 pcw fetal retinae. Analysis was performed on retinal tissue sections (C, E, E′, and G–H′). Scale bar, 50 μm. Whole-mount retina (D and F). Scale bar, 100 μm. White arrowheads in (H) highlight mutually exclusive expression of L/M-OPSIN and RHODOPSIN and in (J and K) indicate different location of RXRG- and NRL-expressing cells. The AAV2/9 pR2.1:GFP reporter labels L/M-opsin cones of 12 pcw (+7 days *in vitro*) fetal retinal explant (L) (arrowheads). GFP+ cells also co-label with the early cone marker RXRG (M) (arrowheads) but do not co-localize with S-OPSIN (N) (arrowheads) or NR2E3 (O) (arrowheads). RPE, retinal pigment epithelium; NBL, neuroblastic layer; ONBL, outer neuroblastic layer; INBL, inner neuroblastic layer; ONL, outer nuclear layer; OPL, outer plexiform layer; INL, inner nuclear layer; IPL, inner plexiform layer; GCL, ganglion cell layer (see Figures S1–S3).

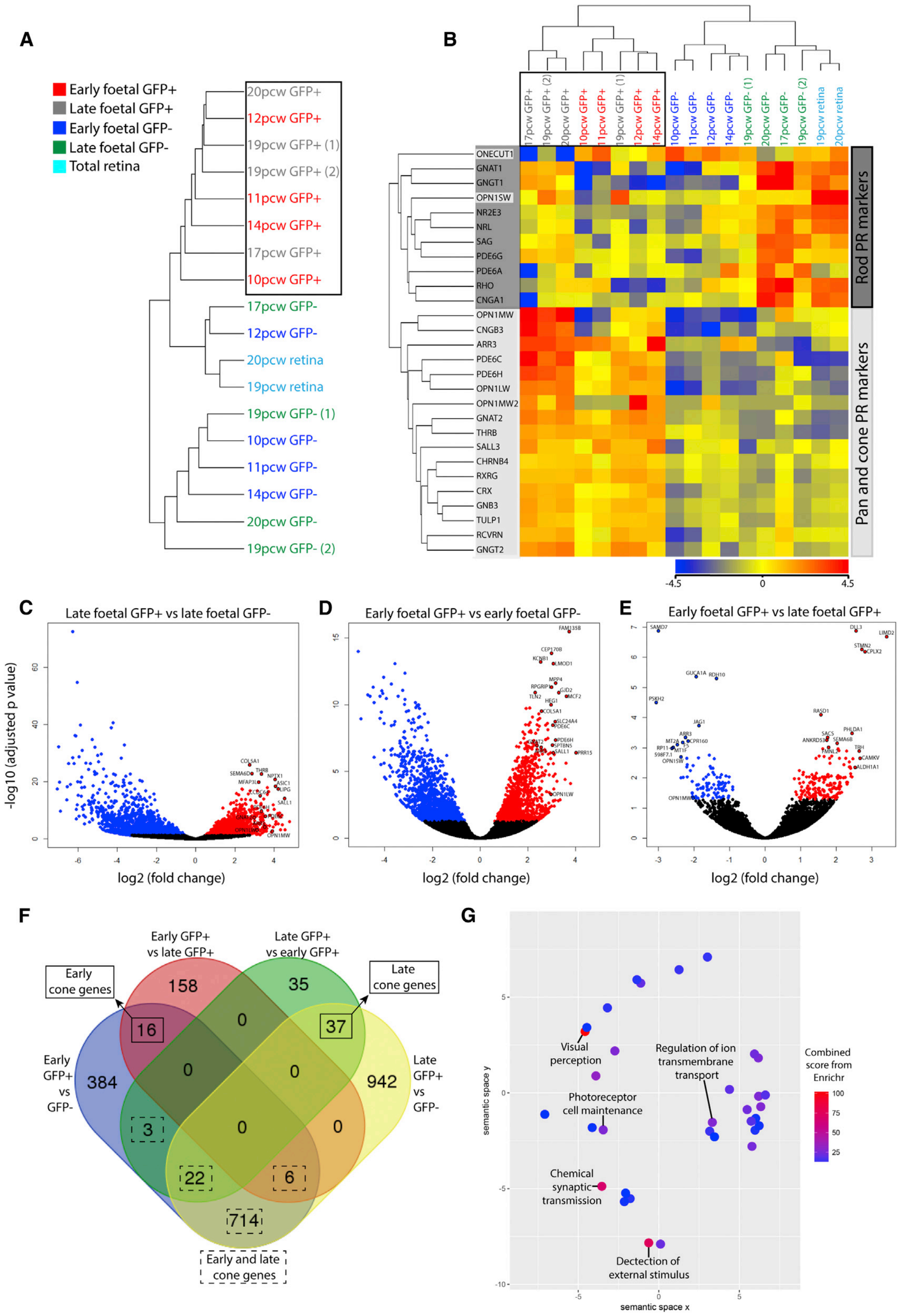

(legend on next page)

in early cone photoreceptor development, *RXRG*, *THRB*, *SALL3*, and *ONECUT1*, showed varied expression levels during retinal development relative to 9 pcw and all were downregulated in the adult retina (Figure 1A). By contrast, markers of mature cone photoreceptors, the cone opsins (*OPN1SW* and *OPN1LW/MW*) and cone arrestin (*ARR3*) showed substantial upregulation in late fetal retinal samples (19 and 20 pcw; Figure 1B) and in the mature adult retina, with *OPN1LW/MW* expression showing the greatest fold change (11,000-fold) during development. Cone transducin (*GNAT2*) and the pan-photoreceptor markers, cone-rod homeobox gene (*CRX*) and recoverin (*RCRVN*), showed modest upregulation during development (Figures 1B and S1A).

Immunohistochemical analysis of cone, pan, and rod photoreceptor markers in fetal retinal tissue highlighted differences in retinal maturity and photoreceptor cell density in the central compared with the peripheral retina, with later detection of protein compared with transcript detection (Figures 1C–1K and S1). CRX and RECOVERIN protein were detected in the 9–19 pcw retina, with a greater density of highly expressing cells in the central retinal region compared with the periphery (Figure S1B). Conversely, proliferation marker KI67 was detected in the peripheral, but not central, retinal cells by 19 pcw (Figure S1C). Of the cone markers, only ONECUT1 protein was detected by 9 pcw in cells across the neuroblastic layer (Figure 1C). S-OPSIN protein was detected from 13 pcw onward (Figures 1D–1E′), followed by L/M-OPSIN from 17 pcw, each appearing first only in the central retina (Figures 1F–1H). By 19 pcw, L/M-OPSIN- and S-OPSIN-labeled cone photoreceptors of the central retina showed a distinct morphology compared with the peripheral regions (Figures 1E, 1E′, 1G, and 1G′). L/M-OPSIN and rod marker RHODOPSIN showed mutually exclusive immunostaining (Figures 1H and 1H′). GNAT2 localized to the apical region of the outer nuclear layer (ONL) in cone developing outer segments (Figure 1I); RXRG localized to the outermost cone cell bodies of the central ONL (Figure 1J), whereas rod markers, NRL and NR2E3, showed distinct immunostaining of developing rod cell bodies (Figures 1K and S1D).

Transcriptome Analysis of Human Fetal L/M-Opsin Cone Photoreceptors

Having defined the progressive appearance of L/M-OPSIN-expressing cones in the human fetal retina and the paucity of early human cone markers, we sought to isolate and characterize the transcriptome of these cells using the AAV2/9.pR2.1:GFP reporter. The pR2.1 promoter contains the locus control region and enhancer regions found 5′ upstream of L- and M-opsin genes (*OPN1LW, OPN1MW*, and *OPN1MW2*) on the X chromosome (Nathans et al., 1989; Wang et al., 1992). It drives expression in canine and rat L/M-opsin cone photoreceptors (Komaromy et al., 2008; Li et al., 2008), but also within mouse M- and S-cones (Wang et al., 1992; Fei and Hughes, 2001). To test the specificity of the reporter within the human retina, we developed a free-floating explant culture system that supports the viability and integrity of fetal retina during the 7 day period required to achieve AAV vector expression (Figures S2A–S2C). GFP+ cells were observed 3–4 days *in vitro* after delivery of the reporter to early (12 pcw) and late (19 pcw) human fetal retinal explants (Figures S2D and S2E); cryosections showed GFP+ cells in the ONL, co-labeling with L/M-OPSIN protein (Figure 1L). GFP+ cells were also positive for early cone-specific marker, RXRG (Figure 1M), but negative for S-OPSIN (S-cone marker; Figure 1N), NR2E3 (rod marker; Figure 1O) and proliferation marker, KI67 (Figure S2F), indicating the specificity of the reporter virus to post-mitotic L/M-opsin cone cells (Figure S2G). The reporter also effectively labeled L/M-opsin cones in fetal retinae (14% of 14 pcw and 4% of 18 pcw cones labeled; Figures S3A and S3B).

Early (n = 4) and late (n = 4) fetal retinal samples labeled with the AAV2/9 pR2.1:GFP reporter were treated by FACS (Figure S3C) to isolate the GFP+ and GFP− cells for RNA-seq, enabling the identification of highly expressed and enriched genes of the human L/M-opsin cone cell transcriptome. Unbiased hierarchical clustering analysis based on normalized gene expression showed that all GFP+ samples cluster together (Figure 2A, black box). Similarly, hierarchical clustering analysis based on the expression of selected established markers of cone, pan, and rod photoreceptors (n = 28) revealed all GFP+ samples cluster together

Figure 2. Transcriptome Analysis of Human Fetal AAV2/9 pR2.1:GFP-Labeled Cells

(A–E) All fetal AAV2/9 pR2.1:GFP+ samples cluster together based on total transcript expression (A) (black box) and high expression of cone and pan-photoreceptor (PR) genes (B) (light gray box). Lower levels of rod-associated genes are detected in pR2.1:GFP+ samples (B) (dark gray box; *ONECUT1* and *OPN1SW* also group with the rod genes). Volcano plots representing the differential gene expression between late GFP+ and GFP− samples (C), early GFP+ and GFP− samples (D) and early GFP+ and late GFP+ samples (E). Significantly upregulated and downregulated genes (adjusted p value < 0.05) are highlighted in red and blue.

(F) Venn diagram represents the overlap between significantly upregulated genes identified for the fetal GFP+ samples, revealing the 798 cone-enriched gene signature.

(G) Revigo semantic plot demonstrates the enriched biological process GO terms associated with the cone gene signature. Color represents the combined score from Enrichr (see Figures S2 and S3; Table S1).

Table 1. Notable Genes from Cone-Enriched Gene Signature with High Fold Changes

Cone Population	Function	Genes	Fold Change
Upregulated in early and late cones	known cone photoreceptor associated genes	*OPN1MW*	15
		PDE6C	11.7
		GUCA1A	12.8
		PDE6H	10.3
		ARR3	12.1
		RXRG	5
		THRB	10.1
		GNAT2	7.9
		CNGB3	18.5
		PDE6H	10.3
	known photoreceptor associated genes	*MPP4*	16
		RP1L1	8.9
		CACNA2D4	10.6
		LRIT3	11.1
		SLC12A5	10.1
		SAMD7	5.8
		SLITRK6	6.2
		CC2D2A	7.8
		RGS11	18.6
		RPGRIP1	12.6
		EGFLAM	10.1
		RD3	9.1
	development	*SALL1*	23.3
		PRR15	16.1
		GREM2	9.1
		KREMEN2	11
		HEG1	9.8
		LIPG	18.5
		BMP7	7.1
		THBS4	8.5
		SLC39A5	18.1
	synaptic processing/neurotransmission	*NPTX1*	16.7
		KCNB2	13.8
		KCNB1	5.8

(*Continued on next page*)

Table 1. *Continued*

Cone Population	Function	Genes	Fold Change
		ASIC1	16.7
		KCNG2	9.6
	non-coding RNA genes	*CTD-2521M24.11*	27.5
		CTD-2034I21.1	21.5
		CTD-2050N2.1	12.4
		TNK2-AS1	9.8
Upregulated in early cones	neural function	*DDN*	6
	negative regulator of Wnt signaling	*AMER2*	2.7
	nervous system development	*SEMA6B*	3.4
	synaptic processing	*CPLX2*	2.5
Upregulated in late cones	ECM/cell adhesion	*TNC*	3.2
		PCDHAC1	8.5
		MATN2	7.2
	photoreceptor outer segment	*RGS9BP*	5.6
	phototransduction	*RGS9*	5.1
	cilia function	*RSPH4A*	4
	development	*GSC2*	19.9
	interkinetic nuclear migration	*CEP120*	3
	cell differentiation and proliferation	*ST3GAL5*	2.7

Function and fold changes are provided of significantly upregulated genes with high fold changes that feature within cone-enriched gene signature. The full cone-enriched gene signature is provided in Table S2.

(Figure 2B, black box), based on their high expression of cone- and pan-associated genes (Figure 2B, light gray genes). By contrast, the late GFP− and total retinal samples showed a higher expression of rod genes, particularly at later time points (Figure 2B, dark gray genes). Noteworthy, was the expression of *OPN1SW* in some of the late GFP+ samples. Together, these data support a cone identity for the isolated GFP+ cells from human fetal retinal explants labeled by the AAV2/9.pR2.1:GFP reporter.

Differential gene expression analyses were performed to identify genes highly enriched in early and late fetal GFP+ cones and genes differentially expressed between early and late cone populations (adjusted p value < 0.05). We identified 1,721 and 1,145 genes that were significantly upregulated in the late and early GFP+ cone populations, respectively, compared with the GFP− samples (Figures 2C and 2D, red data points; Table S1). Comparison of early versus late GFP+ expression profiles identified 180 and 96 genes upregulated in the early and late GFP+ cones, respectively (Figure 2E, red and blue data points, respectively; Table S1). Significantly upregulated genes with high fold changes are labeled on volcano plots and highlighted in Table S1. Merging these datasets identified 745 genes that are enriched in both early and late cone populations, in addition to 16 and 37 genes that are enriched in the early or late human cone populations, respectively (Figure 2F; Table S2). This set of 798 genes defines a unique cone-enriched gene signature, and includes genes with high fold enrichment that have established roles in cone and pan-photoreceptor functioning, developmental processes, synaptic processing and neurotransmission, extracellular matrix (ECM), and cell adhesion, in addition to genes with currently undefined function (Tables 1 and S2).

Gene ontology (GO) analysis on the 798 gene cone signature identified a high enrichment of biological processing GO terms for visual perception, detection of external stimulus, chemical synaptic transmission, photoreceptor cell maintenance, and regulation of ion transmembrane

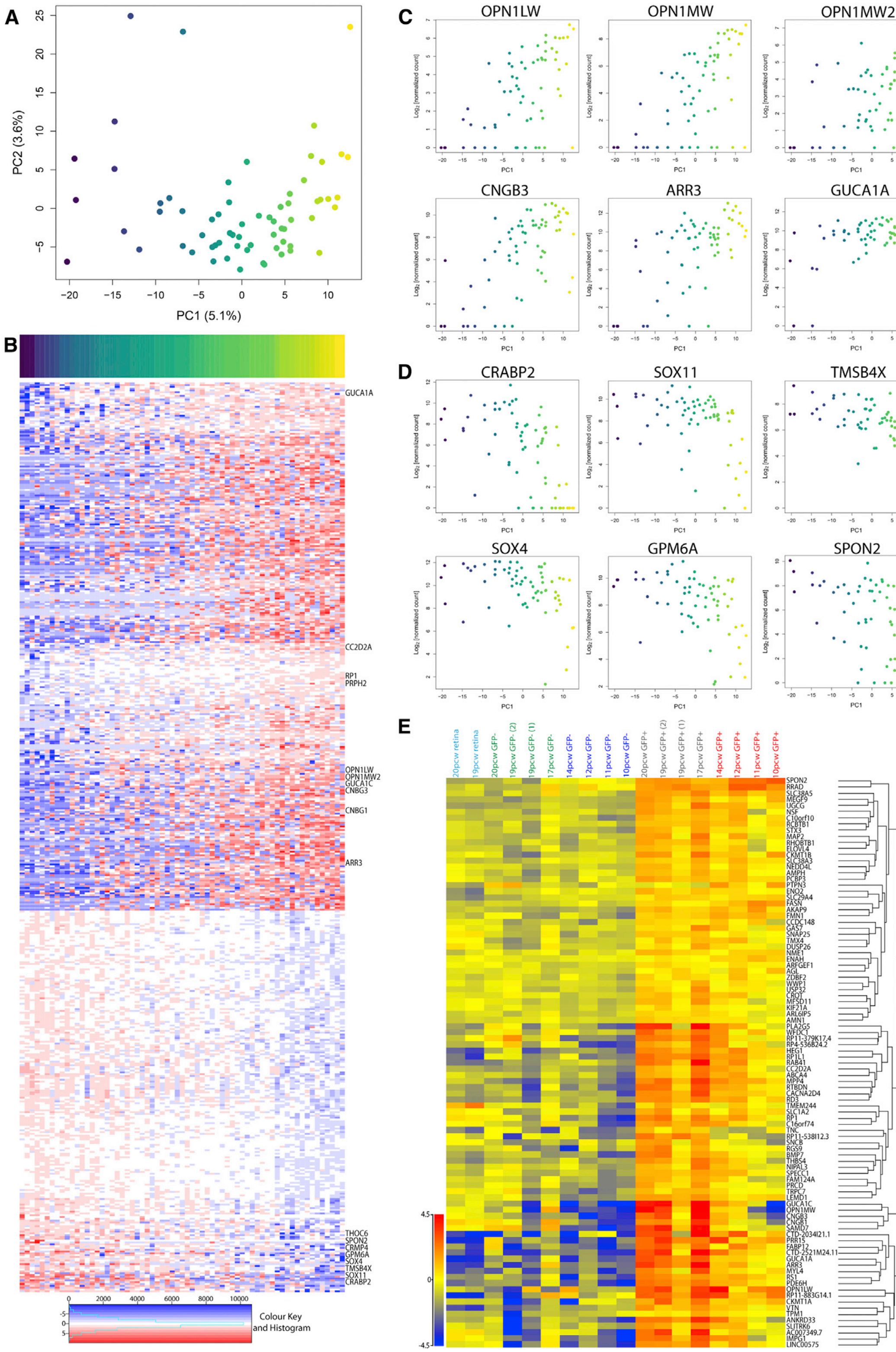

Figure 3. Single-Cell Transcriptome Analysis of AAV2/9 pR2.1:GFP+ Cells

(A) Principal component analysis (PCA) plot of 65 individual AAV2/9 pR2.1:GFP+ cells from 15 pcw fetal retina, where each cell is colored based on its PC1 location (blue to yellow). The proportion of variance explained by the first two principal components is shown in brackets.

(legend continued on next page)

transport (Figure 2G; Table S3). GO terms containing the known cone regulatory genes, *RXRG*, *THRB*, and *RORA*, identified other genes associated with the "steroid hormone-mediated signaling pathway," including *BMP7* and *NR2F6* (Table S3, genes in bold), which are without known function in cones.

Single-Cell Transcriptome Analysis of Human Fetal L/M-Opsin Cone Photoreceptors

We next performed single-cell RNA-seq on the AAV2/9. pR2.1:GFP reporter-labeled population to explore the degree of cell heterogeneity at a single time point. The bulk RNA-seq analysis indicated the labeling of cells expressing *OPN1LW*, *OPN1MW*, *OPN1MW2*, and *OPN1SW* genes. Principal component analysis was performed to determine any systematic substructure within the pR2.1:GFP+ cell population, which revealed a continuum of cells with modest variability based on principal component 1 (PC1) (Figure 3A). Differential gene expression analysis correlated to PC1 revealed 503 significantly differentially expressed genes (false discovery rate < 0.05 Table S4), which showed an upregulation or downregulation as cells progress through PC1 (Figure 3B). We found that the expression profiles of genes that were significantly differentially expressed along PC1 correspond to increasing maturation across this population. Within the upregulated gene group, we noted a number of established genes associated with photoreceptor maturation including outer segment disc morphogenesis (*RP1* and *PRPH2*), primary cilium development (*CC2D2A*), phototransduction proteins (*CNBG3*, *CNGB1*, *GUCA1A*, *GUCA1C*, and *ARR3*), in addition to the L- and M-opsin genes (*OPN1LW* and *OPN1MW2*) (Figures 3B and 3C). In contrast, a number of genes that showed an overall downregulation across PC1 were associated with developmental processes, including transcription factors, *SOX4* and *SOX11*, retinoic acid binding protein, *CRABP2*, neurodevelopment disease gene, *THOC6*, cell migratory and neural differentiation-related genes, *TMSB4X*, *GPM6A*, *CRMP4,* and neural expressed ECM gene, *SPON2* (Figures 3B and 3D). Established pan (*RCVRN* and *CRX*) and cone photoreceptor genes (*RXRG* and *THRB*) did not significantly differ across PC1 (Figures S4A and S4B), which is consistent with their cone cell identity. S-cone (*OPN1SW*) and rod photoreceptor (*NRL* and *NR2E3*) gene expression, required for other photoreceptor cell fates, was detected in a small number of individual cells (Figure S4C).

We next examined whether the continuum of naive and mature cell states identified at a single time point represent true developmental trajectories. We found that 18.5% (93/503) of the differentially expressed genes identified in the single-cell analysis showed changed expression across developmental time when considering the early and late bulk mRNA transcriptome data (Figure 3E), including a large number of genes not previously reported in cone cells (Table S2, green highlighted genes). Hence, these data are consistent with the proposal that, at a single point in developmental time, individual cone cells exist in the retina at different stages of their developmental trajectory as revealed by their gene expression profiles.

Comparative Transcriptomic Analysis with Human iPSC-Derived Cone Photoreceptors

Having defined the transcriptomic signature of a human fetal L/M-opsin cone photoreceptor population, we applied the same approach to human iPSC-derived cone photoreceptor cells to assess the degree of similarity between *in vivo* and *in vitro* generated cones. To generate cone photoreceptors from iPSCs, we used a retinal organoid differentiation culture system (Meyer et al., 2009) that generates optic vesicle structures containing RECOVERIN and CRX-expressing photoreceptor cells from week 6 of differentiation (Figure S5A). Expression of cone markers, detected in the human fetal retinal sample series, were confirmed by qRT-PCR (*RXRG, THRB, SALL3, ONECUT1*, *OPN1LW/MW, ARR3,* and *GNAT2;* Figure S5B). *OPN1LW/MW* expression was upregulated by week 14 of differentiation and maintained into later stages of culture (weeks 21–28). Immunohistochemistry revealed labeling for photoreceptor markers, RECOVERIN and CRX, in addition to cone markers, ONECUT1 and L/M-OPSIN, within optic vesicle structures by week 14 (Figures 4A–4C) and in dissociated late-stage cultures for L/M-OPSIN (Figure 4D). Comparable levels of *OPN1LW/MW* and *ARR3* expression were detected at early (12–14 pcw) and late (19–20 pcw) developmental time points compared with weeks 14 and 21 iPSC-derived retinal differentiation cultures, respectively; however, OPN1LW/MW showed significantly higher expression within the late-stage human fetal retina (Figure 4E).

(B) Heatmap of genes that are significantly differentially expressed (false discovery rate < 0.05) with respect to PC1. Columns represent single cells, which are ordered from left to right by their location on PC1. Each row represents a gene that is upregulated (blue to red; first block) or downregulated (red to blue; second block) along PC1.
(C and D) Scatterplots showing gene expression (log2 normalized counts) profiles of key significantly differentially expressed genes which show upregulation (C) or downregulation (D) along PC1.
(E) Heatmap displaying expression profiles of the 93 differentially expressed genes identified from single-cell RNA-seq analysis which feature within the cone-enriched gene signature (see Figure S4; Tables S2 and S4).

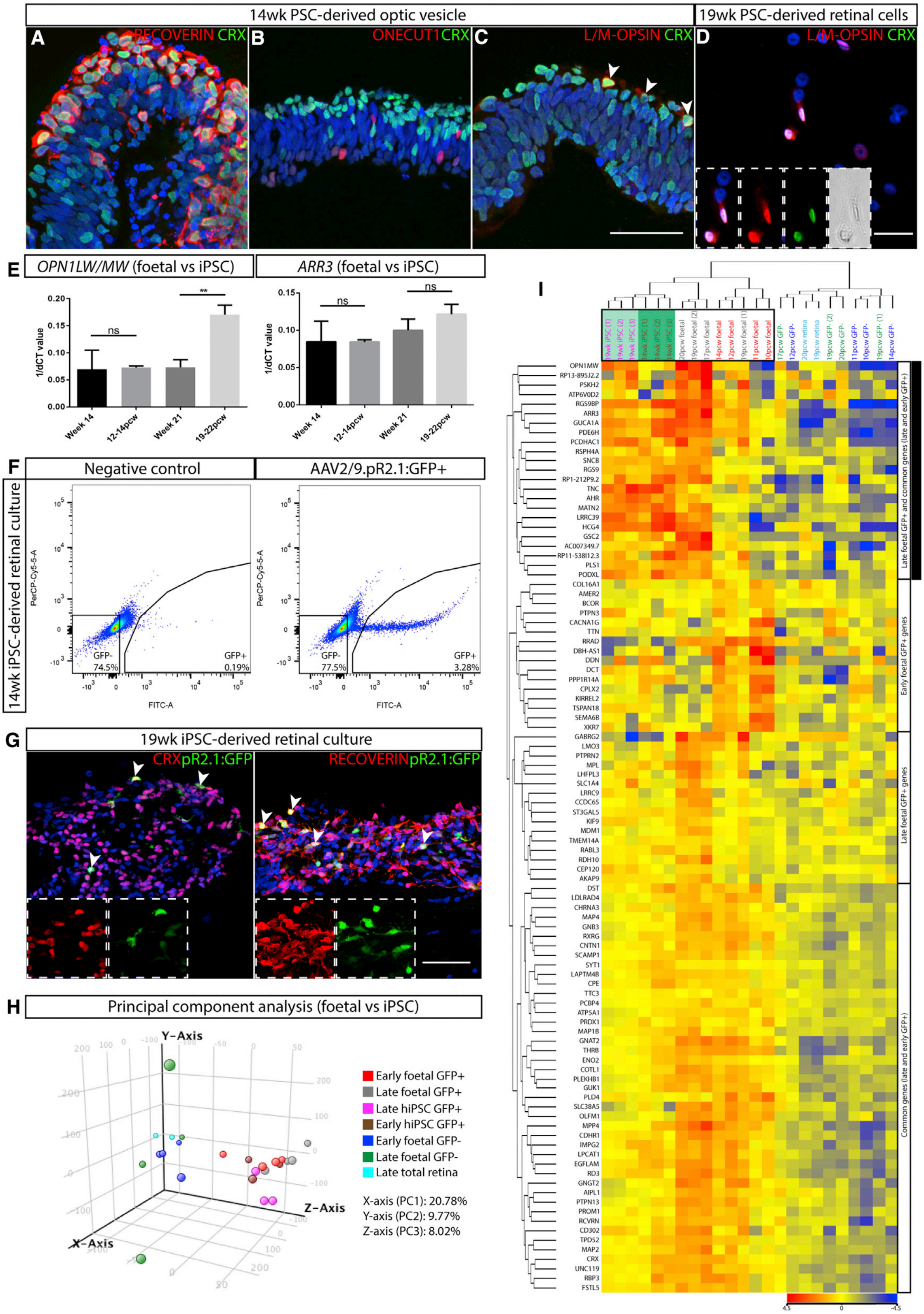

(legend on next page)

The AAV2/9.pR2.1:GFP virus was used to transduce early (weeks 14–15) and late (weeks 19–20) iPSC-derived retinal differentiation cultures, and pR2.1:GFP+ cells were isolated for RNA-seq analysis using FACS (Figure 4F) and showed co-labeling with photoreceptor markers, CRX and RECOVERIN (Figure 4G, white arrows). Principal component analysis showed clustering of the fetal and iPSC-derived GFP+ samples together, away from total fetal retina and GFP− samples (Figure 4H). Hierarchical clustering analysis performed with the newly identified fetal cone-enriched gene signature revealed that iPSC-derived GFP+ samples were most similar to the fetal GFP+ samples (Figure 4I; black box). Specifically, genes enriched within the late fetal cones show the most comparable expression profiles within both weeks 14–15 and 19–20 iPSC-derived GFP+ samples and the late-stage fetal samples (Figure 4I, 21/37 genes; top cluster; filled black box). Notably, the early cone-enriched genes (Figure 4I, 16 genes; second cluster) showed overall low expression within the iPSC-derived cells, together with the remainder of the late cone-enriched genes and the common cone gene set (Figure 4I, third and fourth clusters). Overall, these data suggest a cone identity of the iPSC-derived cells and provide valuable information on their stage of differentiation and degree of similarity to fetal cones.

Identification and Application of Cell Surface Markers to Isolate Human Fetal and iPSC-Derived Cone Photoreceptors

Next, we used a dual approach to identify cell surface markers expressed by fetal pR2.1:GFP+ cells, which could provide tools to enrich for human cone photoreceptors without the need for genetic manipulation. We created a cell surface marker protein query database of 3,367 putative cell surface proteins, which was used to identify 99 and 170 potential cell surface marker genes enriched in the early and late fetal pR2.1:GFP+ cells, respectively (Table S5). Of these, 16 were cluster of differentiation (CD) markers (Table S5; highlighted); 13 and 7 were identified in the late and early enriched cone gene sets respectively, with 4 CD markers, *DPP4* (CD26), *PROM1* (CD133), *ITGA4* (CD49d), and *JAM2* (CD322), featuring in both populations. Of these, *DPP4* (CD26) and *PROM1* (CD133) were highly expressed and significantly upregulated in the late-stage cones and the remainder represent potential cone cell surface markers (Table S5). In addition, we directly screened early (12 pcw) and late (17 pcw) fetal retinae labeled with the AAV2/9 pR2.1:GFP reporter for 242 different human CD marker antibodies using the BD Lyoplate screening panels. We identified markers that labeled and caused a discrete flow cytometry shift of at least 50% of the pR2.1:GFP+ cell population (Figure 5A). Six CD markers (CD57, CD47, CD59, CD200, CD151, and CD98) were common across the 12 and 17 pcw samples (Figure 5B), and eight additional markers (CD63, CD26, CD147, CD120a, CD81, CD49c, CD90, and CD165) labeled the 17 pcw pR2.1:GFP+ cells (Figure 5C). CD26 overlapped with the CD markers identified from the cone gene signature and the remaining 12 CD markers were detected within the cone transcriptome data, except CD165. Notably, some CD markers displayed greater specificity to pR2.1:GFP+ cells at later fetal time points, i.e., labeling a higher percentage of GFP+ cells and lower percentage of GFP− cells, including markers CD26 and CD147 (Figure 5C, black box). CD133 robustly labeled 13 and 14 pcw pR2.1:GFP+ cells (Figure 5D).

CD26, CD133, and CD147 were selected for testing to determine if they could enrich for late-stage human L/M-opsin cones. Double immunocytochemistry on dissociated human fetal retina revealed that CD26, CD133, and CD147 co-label with some, but not all, L/M-OPSIN-expressing cells (Figure 5E, white arrows). Some L/M-OPSIN-negative cells were also labeled by the CD markers. In FACS experiments, the CD markers

Figure 4. Generation of PSC-Derived Cone Photoreceptors via 3D Retinal Differentiation Culture System
(A–D) Immunohistochemistry analysis showing detection of photoreceptor markers, RECOVERIN and CRX (A), and cone markers, ONECUT1 (B) and L/M-OPSIN (C), within 14-week PSC-derived vesicles. At 26 weeks, L/M-OPSIN can also be detected within dissociated cultures (D), which also express CRX (C and D; arrowheads).
(E) Comparative qRT-PCR analysis of *OPN1LW/MW* and *ARR3* at age-matched time points between fetal and iPSC-derived retinal samples (14 weeks and 12–14 pcw; 19 weeks and 19–22 pcw), $^{**}p = 0.0008$. All error bars represent the SD.
(F) Representative FACS traces of negative control and 14 weeks iPSC-derived retinal differentiation culture labeled with the AAV2/9.pR2.1:GFP reporter.
(G) Representative images showing co-labeling of iPSC GFP+ cells with photoreceptor markers CRX and RECOVERIN (arrowheads) within 20 week retinal differentiation culture.
(H) PCA plot of fetal and iPSC-derived GFP+ samples based on total gene expression.
(I) Heatmap representation of hierarchical clustering analysis performed with fetal and iPSC-derived samples based on the cone-enriched gene signature. Genes featured on the heatmap represent all the early (16 genes), late (37 genes), and most enriched genes common to early and late cones (44 genes with $p < 0.05$, highest fold changes and high expression from early and late GFP+ versus GFP− comparisons). Scale bar, 50 μm. See Figure S5 and Table S2.

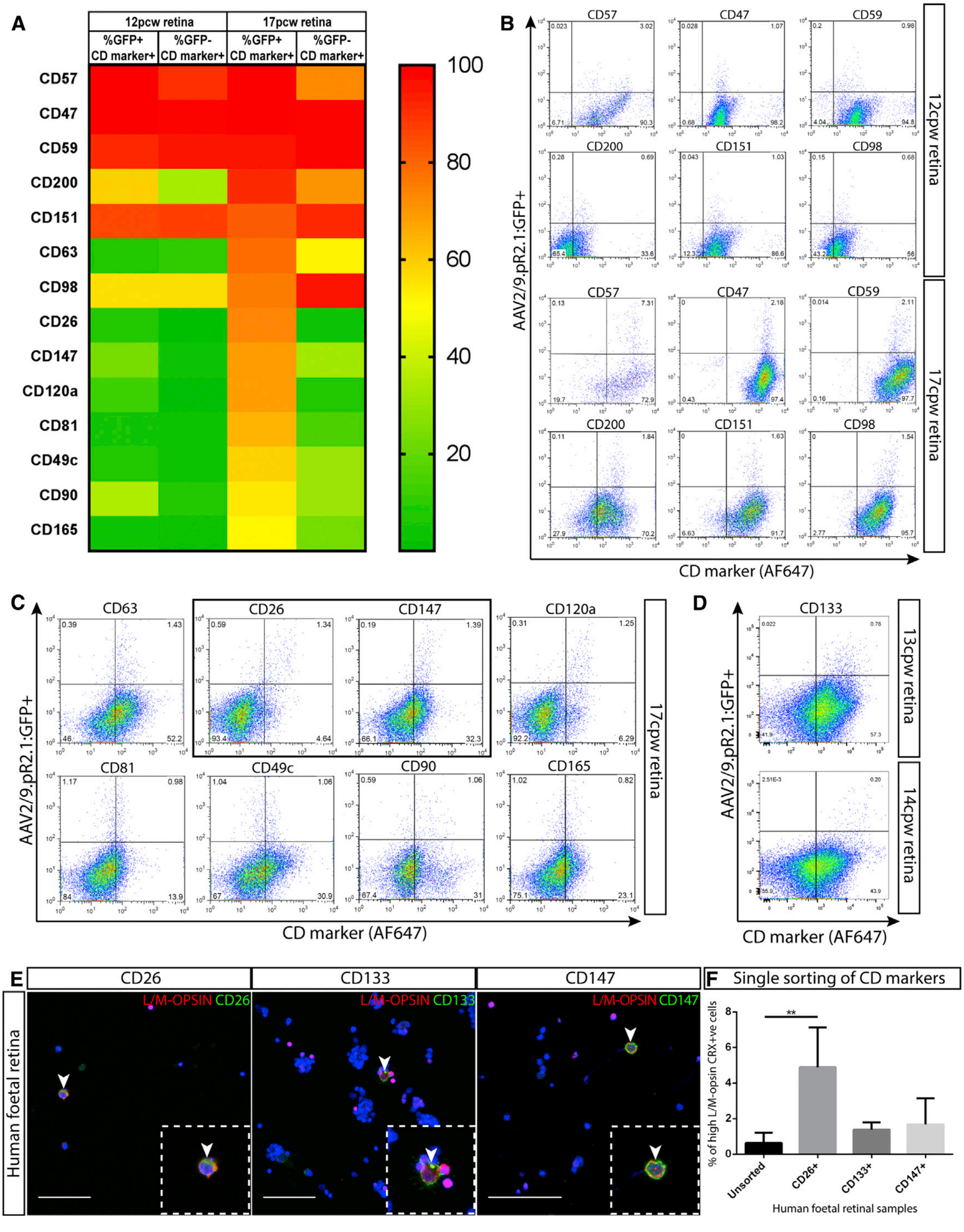

Figure 5. Identifying and Profiling CD Makers in the Human Fetal Retina

(A) Heatmap showing the cell percentage labeled by the 14 CD markers identified from the antibody screening panels. GFP+/CD marker+ percentage is representative of the total labeled pR2.1:GFP+ population and the GFP−/CD marker proportion is based on the remaining retinal cell population.

(B) Flow cytometry traces of the 6 CD markers labeling 12 and 17 pcw pR2.1:GFP+ cells.

(C) Flow cytometry traces of the 8 CD markers labeling GFP+ cells within the 17 pcw retina; CD26 and CD147 are highlighted by the black box.

(D) Flow cytometry traces of CD133 labeling of 13 and 14 pcw pR2.1:GFP+ cells.

(legend continued on next page)

individually labeled a proportion of the total fetal retinal cells (CD26+ 2.9% ± 1.6%; CD147+ 23.1% ± 12.4% and CD133+ 71.1% ± 7.2%); these sorted cells showed a greater percentage of L/M-OPSIN and CRX+ cells compared with the unsorted population (CD26+ 4.9% ± 2.23%; CD133+ 1.38% ± 0.41%; CD147+ 1.67% ± 1.47%, unsorted 0.63% ± 0.59%), with the CD26+ population showing statistical significance (Figure 5F). This overall low level of L/M-opsin cone enrichment prompted the combined sorting of these CD markers to assess if a greater enrichment could be achieved. We detected a triple-positive cell population for all three CD markers via flow cytometry within late-stage fetal retinae and performing immunocytochemistry on this population post FACS revealed enrichment of L/M-OPSIN/CRX+ cells (CD133+/CD26+/CD147+ 8.69% ± 9.62%) compared with the other collected cells (CD133–CD26–CD147– 1.67% ± 1.93%; CD133+/CD26–/CD147– 0.64% ± 0.72%) and the unsorted population (0.45% ± 0.59%; Figures 6A and 6B). To enhance enrichment, we added a further CD marker for negative selection, SSEA-1, which was not detected in pR2.1:GFP+ cells in the proteomic analysis (Figure S6) and has been previously used to remove mitotically active cells (Lakowski et al., 2015; Tucker et al., 2011). By sorting fetal retinal cells negative for SSEA-1, but positive for CD133, CD26, and CD147, an improvement in the percentage of L/M-OPSIN/CRX+ cells within the enriched cell population was achieved (30% ± 16.13%; $p < 0.0002$) when compared with the unsorted population (0.54% ± 0.8%) and the remaining sorted cell populations (CD133–/CD26–/CD147–/SSEA1– 0%; CD133+/CD26–/CD147–/SSEA1– 0.69% ± 1.2%; and SSEA1+ 0.27% ± 0.49%; Figures 6C and 6D).

Finally, we tested the fetal cone CD marker combination on stem cell-derived retinal differentiation cultures generated using the standard protocol (Meyer et al., 2009) for iPSC, or a newly developed protocol for ESC retinal differentiation, which shows improved levels of cone photoreceptor maturation (Gonzalez-Cordero et al., 2017) (Figures S7A and S7B), to assess if a similar CD marker-labeled population of cells was generated by the *in vitro* systems. Figure S7C shows representative flow cytometry traces comparing the SSEA1–CD133+ CD26+ CD147+-labeled populations between fetal and PSC-derived retina. The ESC-derived retinal cultures using the newly developed protocol showed the most similar FACS trace to those observed from the fetal retina and was used for subsequent cell-sorting experiments (week 17–18 of culture, n = 3). Performing immunocytochemistry on this sorted cell population revealed a significant enrichment of CONE ARRESTIN/CRX+ cells (55.1% ± 30.7%; $p = 0.0036$) compared with the unsorted (8.4% ± 3.1%) and additional cell populations (SSEA1– CD133– CD26– CD147– 3.7% ± 1.2%; SSEA1– CD133+ CD26– CD147– 2.3% ± 0.8; SSEA1+ 2.4% ± 2.0%; Figures 6E and 6F). In addition, CONE ARRESTIN-expressing cells labeled by CD26, CD133, and CD147 were readily detected within dissociated ESC-derived retinal cells (Figure 6G, white arrows), similar to observations within the fetal retina. These data indicate that the cone CD marker panel discovered within the fetal retina can be applied to ESC-derived retinal differentiation cultures to enrich for human cone photoreceptor cells.

DISCUSSION

Two important pre-clinical evaluations that need to be addressed for the development of a human cone cell-replacement therapy include assessing whether iPSC-derived cone cells recapitulate a *bona fide* cone photoreceptor transcriptome and the development of a cone cell isolation strategy. Here, we provide insight into the human fetal L/M-opsin cone transcriptome and used this as a baseline reference to show similarities in cone gene expression within iPSC-derived photoreceptors. From these data, we also developed a cell surface marker sorting strategy for the enrichment of human L/M-opsin cone photoreceptors that can be translated to stem cell-derived retinal differentiation culture system.

Our definition of a unique cone-enriched gene signature for human fetal cone photoreceptors relied on the use of an AAV2/9.pR2.1:GFP reporter. This viral reporter showed preferential labeling of human L/M-opsin cone photoreceptors. Rod-associated and S-opsin transcripts were also detected, consistent with previous reports of shared molecular markers between different cone and rod cells (Applebury et al., 2000; Cornish et al., 2004; Craft et al., 2014; Kim et al., 2016). These data suggest a plastic or "noisy" nature of some human photoreceptors, which may continue to express transcripts of other photoreceptor types during fetal development. Exploring the heterogeneity of individual cone cells at a single time point revealed their separation based on maturity, identifying differences in gene

(E) CD26, CD133, and CD147 labeling of L/M-OPSIN-expressing cells (arrowheads indicate double-labeled cells) in the 17 pcw fetal retina via immunocytochemistry. Scale bar, 50 μm.
(F) Counting of L/M-OPSIN/CRX+ cells in single FACS experiments with CD26, CD133, and CD147 (n = 3; 17–20 pcw), **$p = 0.0044$. All error bars represent the standard deviation. See Table S5.

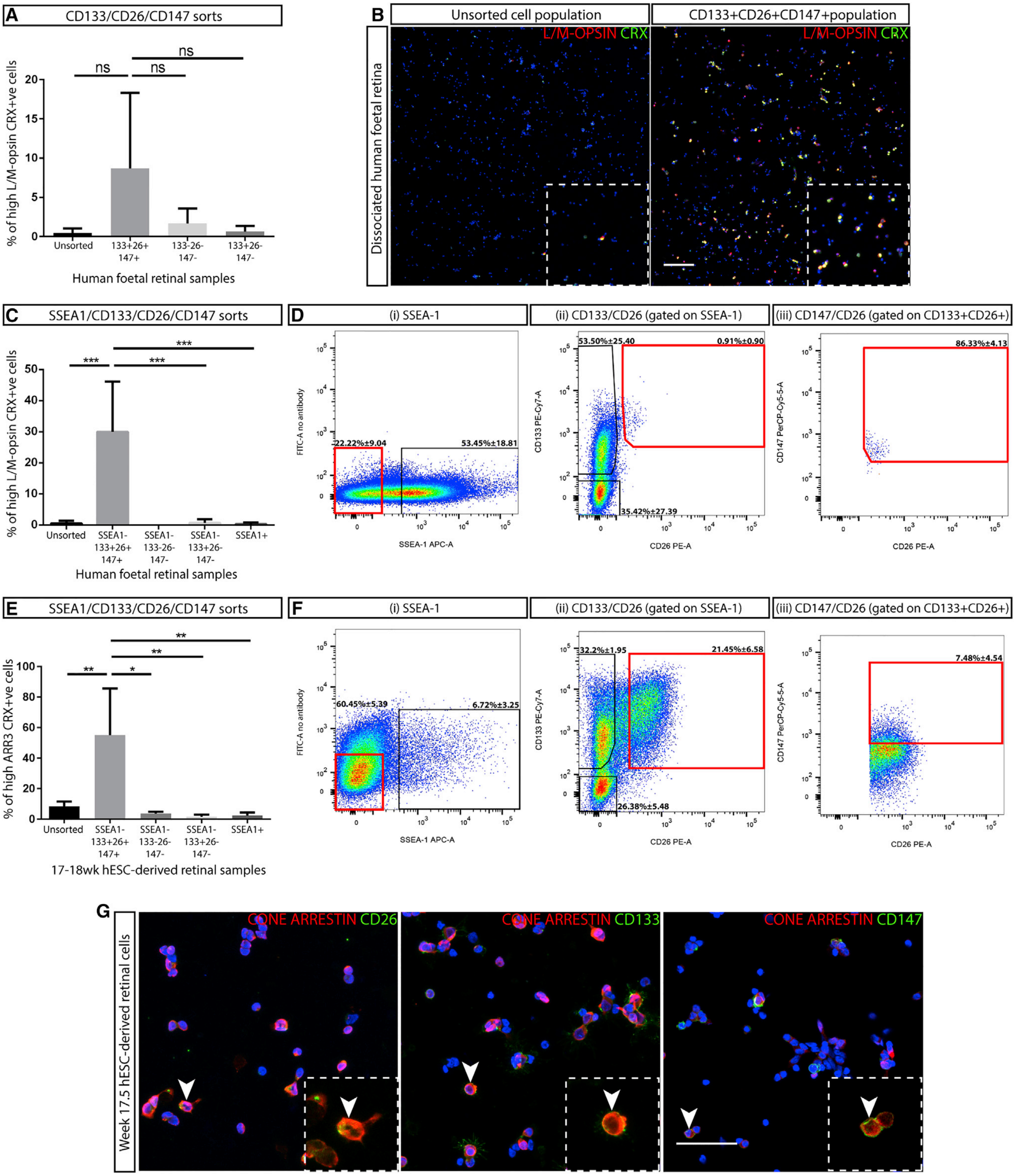

Figure 6. Application of Cone CD Marker Sorting in Human Fetal and ESC-Derived Retina

(A) Counting of fetal L/M-OPSIN/CRX+ cells post FACS with the CD133/CD26/CD147 combination (n = 4); p = 0.1067. ns, not significant. All error bars represent the SD.

(legend continued on next page)

expression profiles of naive and more mature cones, rather than distinguishing cells based on their specific type of cone opsin gene expression. This difference likely reflects the striking gradient of cone photoreceptor maturation observed across the central-peripheral axis in the human fetal retina (Figures 1 and S1) (Xiao and Hendrickson, 2000).

Both the bulk and single-cell RNA-seq data validated 93 cone maturation genes (Table S2, green highlighted genes), including several not previously defined to cone cells. Comparison of our cone-enriched gene signature with pre-existing human adult macula gene expression analyses showed that a number of genes overlap, including transcription factor *NR2F6* and the photoreceptor ribbon synapse protein, *CPLX4* (Bowes Rickman et al., 2006; Sharon et al., 2002). However, many identified genes were not previously reported in adult macula studies (Hornan et al., 2007; Whitmore et al., 2014), which could be due to differences in samples ages, or their inclusion of other retinal cells. Several genes expressed by human ESC-derived CRX.GFP cells (Kaewkhaw et al., 2015), including *SLC38A5, VTN*, and *AMER2*, were also shared with our cone-enriched gene signature.

In the direct comparative transcriptome analysis performed using the cone gene signature, we found similar gene expression profiles between late fetal and iPSC-derived cone cells. However, not all late enriched cone genes showed equivalent levels of expression within the iPSC-derived cells, suggesting an intermediate stage of cone cell differentiation and a relative delay in their maturation. Hence, this approach provides an informative insight into the similarities and differences in gene expression between *in-vivo*- and *in-vitro*-derived cells, and important benchmarking data, which is critical for pre-clinical assessment of stem cell-derived cells for use in clinical therapies.

Toward developing a cone cell purification strategy, we characterized putative cell surface marker genes and CD marker proteins expressed within the AAV2/9.pR2.1:GFP+ human cone populations, providing a benchmarking tool for the differentiation status of retinal cultures. We evaluated a cell isolation strategy for the late cone population, based on the presence of identified cell surface molecules. Both *ST3GAL5* and *EPHA10* genes, previously identified as putative cone surface marker from RNA-seq analysis of human ESC-derived CRX-expressing cells (Kaewkhaw et al., 2015), featured within our significantly enriched late cone dataset. However, CD73, previously used to isolate cone-like cells from $Nrl^{-/-}$ mouse retina (Santos-Ferreira et al., 2015), was not upregulated in the human cones. We found no cell surface markers that exclusively labeled the pR2.1:GFP+ cone population and instead identified CD markers, such as CD26, CD147, and CD133, which labeled a percentage of cone cells as well as other retinal cells. Together with the rarity of cone cells within the human retina, this presents a considerable challenge to achieve high levels of enrichment. Nevertheless, by combining CD markers for positive cone selection (CD26, CD147, and CD133) and negative selection of undesirable cells (SSEA-1), we demonstrate the enrichment of human L/M-opsin cone cells from the human fetal retina. The average enrichment achieved using this combination (30% ± 16.13%) suggests further optimization would be required to increase the purity of cone cell isolation for a clinical transplantation therapy. However, these data demonstrate the feasibility of using a cell surface molecule approach to enrich for human cone photoreceptors. Furthermore, using a new ESC-derived retinal differentiation culture system with higher levels of cone differentiation (Gonzalez-Cordero et al., 2017), we demonstrate the same fetal cone sorting strategy can be applied to provide an enrichment of ESC-derived cone photoreceptors. Together with the identification of a human L/M-opsin cone-enriched gene signature, this study provides an initial pre-clinical assessment comparing *bona fide* and stem cell-derived cones toward developing a cone cell therapy, which may also be informative for other applications, including *in vitro* retinal disease modeling and basic research into human cone photoreceptor development.

(B) Representative images of L/M-OPSIN/CRX immunocytochemistry within the unsorted and CD133+/CD26+/CD147+ sorted cell population. Scale bar, 100 μm.
(C) Counting of fetal L/M-OPSIN/CRX+ cells post FACS with the SSEA1/CD133/CD26/CD147 combination (n = 4; 17–20 pcw); $^{***}p < 0.0002$. All error bars represent the SD.
(D) Representative FACS traces for the SSEA1/CD133/CD26/CD147 combination within the fetal retinae, with cell population percentages. The red rectangular outlines represent gated cell populations in the FACS experiments.
(E) Counting of ESC-derived CONE ARRESTIN/CRX+ cells post FACS with the SSEA1/CD133/CD26/CD147 combination (n = 4, 17–18 weeks); $^{*}p = 0.0146$, $^{**}p = 0.0036$. All error bars represent the SD.
(F) Representative FACS traces for the SSEA1/CD133/CD26/CD147 combination for the ESC-derived retinal cultures. Cell population percentages are provided for (D) and (F), mean represents ± SD.
(G) Immunocytochemistry of CD26, CD133, CD147, and CONE ARRESTIN within 17.5 weeks dissociated ESC-derived retinal cells (white arrowheads indicate co-localization). Scale bar, 50 μm.

EXPERIMENTAL PROCEDURES

Full experimental methods are provided within Supplemental Experimental Procedures.

Human Tissue

Human fetal eyes were obtained from the Joint Medical Research Council UK (grant no. G0700089)/Wellcome Trust (grant no. GR082557) Human Developmental Biology Resource (http://www.hdbr.org/), and human adult eyes were obtained from Moorfields Biobank with ethics approval. iPSCs (NCUS:7) were generated by the NIHR Cambridge Biomedical Research Centre hiPSC Core Facility from an individual's skin fibroblasts with normal ophthalmological examination and with ethics approval. Work on human ESC lines was approved by the UK Stem Cell Bank Steering Committee (Medical Research Council, London, UK).

Application of AAV2/9.pR2.1:GFP Reporter

AAV2/9.pR2.1:GFP virus was added to human fetal retinal explants and iPSC-derived retinal differentiation cultures at an MOI of 40,000–45,000. pR2.1.GFP+ cells were sorted using the BD FACSAria III cell sorter for RNA-seq analysis. CD marker profiles of pR2.1.GFP+ labeled cells were detected using BD Lyoplate screening panels and the BD FACSCalibur.

Total and Single mRNA-Seq and Analysis

The Illumina NextSeq 500 system was used with a targeted sequencing depth of 17 million 43 bp paired end per sample for the bulk RNA-seq and 2 million 75 bp paired end reads per sample for the single-cell RNA-seq. Adjusted p values < 0.05 were used for bulk and single-cell data to determine significance.

Statistics

ANOVA with Bonferroni correction for multiple testing was used to determine statistical significance using GraphPad Prism 6. Adjusted p values are stated within figure legends and all means are displayed ±SD. For qRT-PCR analysis, three biological samples were used for each time point excluding Figure 1, where biological sample is 1 due to the limited availability of fetal tissue. For FACS CD marker analysis, four independent fetal/ESC-derived retinal samples were analyzed. At least 50 cells were counted for FACS cell populations using the ImageJ software.

ACCESSION NUMBERS

The accession number for the bulk RNA-seq data reported in this paper is ArrayExpress: E-MTAB-6057. The accession number for the single cell RNA-seq data reported in this paper is ArrayExpress: E-MTAB-6058.

SUPPLEMENTAL INFORMATION

Supplemental Information includes Supplemental Experimental Procedures, seven figures, and five tables and can be found with this article online at https://doi.org/10.1016/j.stemcr.2017.10.018.

AUTHOR CONTRIBUTIONS

E.W., conception and design, collection and/or assembly of data, data analysis and interpretation, manuscript writing. J.L., conception and design, collection and/or assembly of data, data analysis and interpretation. V.D.F., collection and/or assembly of data. D.B., collection and/or assembly of data. A.G.-C., collection and/or assembly of data. A.T.L.L., data analysis and interpretation. M.E., data analysis and interpretation. A.P., data analysis. E.C., collection and/or assembly of data. K.K., collection and/or assembly of data. A.N., collection and/or assembly of data. F.M., data analysis and interpretation. M.H., collection and/or assembly of data. D.T.J., data analysis. J.C.M., data analysis and interpretation. R.R.A., conception and design, manuscript writing, financial support. J.C.S., conception and design, data analysis and interpretation, manuscript writing, final approval of manuscript, financial support.

ACKNOWLEDGMENTS

We thank A. Eddaoudi and S. Canning at the Flow Cytometry Core Facility and D. Moulding at the Imaging Facility at UCL Great Ormond Street Institute of Child Health, T. Brooks at UCL Genomics, K. Wood at the Genome Center, Queen Mary University of London, and R. Maswood and S. Azam at the UCL Institute of Ophthalmology for technical support. We are very grateful to the NIHR Cambridge Biomedical Research Centre hiPSC Core Facility and to Prof. David Gamm, University of Wisconsin, for support establishing pluripotent stem cell culture and retinal differentiation. This work was supported by the Medical Research Council UK (MR/M015688/1, MR/M007871/1, and MR/J004553/1); New Life; Fight for Sight; the Rosetrees Trust; the National Institute for Health Research (NIHR) Great Ormond Street Hospital Biomedical Research Center (GOSH BRC); NIHR Moorfields Biomedical Research Center; RP Fighting Blindness (GR576); and the European Research Council (2012-ADG_20120314). J.C.S., R.R.A., J.L., E.W., and A.G.-C. with UCL Business PLC submitted a patent application (provisional patent number 1703058.6) based on this work. J.C.S. is funded by the Great Ormond Street Hospital Children's Charity. The views expressed are those of the authors and not necessarily those of the NHS, the NIHR, or the Department of Health.

Received: May 26, 2017
Revised: October 14, 2017
Accepted: October 15, 2017
Published: November 16, 2017

REFERENCES

Applebury, M.L., Antoch, M.P., Baxter, L.C., Chun, L.L., Falk, J.D., Farhangfar, F., Kage, K., Krzystolik, M.G., Lyass, L.A., and Robbins, J.T. (2000). The murine cone photoreceptor: a single cone type expresses both S and M opsins with retinal spatial patterning. Neuron *27*, 513–523.

Bartsch, U., Oriyakhel, W., Kenna, P.F., Linke, S., Richard, G., Petrowitz, B., Humphries, P., Farrar, G.J., and Ader, M. (2008). Retinal cells integrate into the outer nuclear layer and differentiate into

mature photoreceptors after subretinal transplantation into adult mice. Exp. Eye Res. *86*, 691–700.

Bessant, D.A., Ali, R.R., and Bhattacharya, S.S. (2001). Molecular genetics and prospects for therapy of the inherited retinal dystrophies. Curr. Opin. Genet. Dev. *11*, 307–316.

Bowes Rickman, C., Ebright, J.N., Zavodni, Z.J., Yu, L., Wang, T., Daiger, S.P., Wistow, G., Boon, K., and Hauser, M.A. (2006). Defining the human macula transcriptome and candidate retinal disease genes using EyeSAGE. Invest. Ophthalmol. Vis. Sci. *47*, 2305–2316.

Cai, H., Fields, M.A., Hoshino, R., and Priore, L.V. (2012). Effects of aging and anatomic location on gene expression in human retina. Front. Aging Neurosci. *4*, 8.

Cornish, E.E., Xiao, M., Yang, Z., Provis, J.M., and Hendrickson, A.E. (2004). The role of opsin expression and apoptosis in determination of cone types in human retina. Exp. Eye Res. *78*, 1143–1154.

Craft, C.M., Huang, J., Possin, D.E., and Hendrickson, A. (2014). Primate short-wavelength cones share molecular markers with rods. Adv. Exp. Med. Biol. *801*, 49–56.

de Melo, J., Peng, G.H., Chen, S., and Blackshaw, S. (2011). The Spalt family transcription factor Sall3 regulates the development of cone photoreceptors and retinal horizontal interneurons. Development *138*, 2325–2336.

Decembrini, S., Martin, C., Sennlaub, F., Chemtob, S., Biel, M., Samardzija, M., Moulin, A., Behar-Cohen, F., and Arsenijevic, Y. (2017). Cone genesis tracing by the Chrnb4-EGFP mouse line: evidences of cellular material fusion after cone precursor transplantation. Mol. Ther. *25*, 634–653.

Eberle, D., Santos-Ferreira, T., Grahl, S., and Ader, M. (2014). Subretinal transplantation of MACS purified photoreceptor precursor cells into the adult mouse retina. J. Vis. Exp., e50932.

Eberle, D., Schubert, S., Postel, K., Corbeil, D., and Ader, M. (2011). Increased integration of transplanted CD73-positive photoreceptor precursors into adult mouse retina. Invest. Ophthalmol. Vis. Sci. *52*, 6462–6471.

Emerson, M.M., Surzenko, N., Goetz, J.J., Trimarchi, J., and Cepko, C.L. (2013). Otx2 and Onecut1 promote the fates of cone photoreceptors and horizontal cells and repress rod photoreceptors. Dev. Cell *26*, 59–72.

Fei, Y., and Hughes, T.E. (2001). Transgenic expression of the jellyfish green fluorescent protein in the cone photoreceptors of the mouse. Vis. Neurosci. *18*, 615–623.

Fujieda, H., Bremner, R., Mears, A.J., and Sasaki, H. (2009). Retinoic acid receptor-related orphan receptor alpha regulates a subset of cone genes during mouse retinal development. J. Neurochem. *108*, 91–101.

Gonzalez-Cordero, A., Kruczek, K., Naeem, A., Fernando, M., Kloc, M., Ribeiro, J., Goh, D., Duran, Y., Blackford, S.J.I., Abelleira-Hervas, L., et al. (2017). Recapitulation of human retinal development from human pluripotent stem cells generates transplantable populations of cone photoreceptors. Stem Cell Reports *9*, 820–837.

Hendrickson, A., Bumsted-O'brien, K., Natoli, R., Ramamurthy, V., Possin, D., and Provis, J. (2008). Rod photoreceptor differentiation in fetal and infant human retina. Exp. Eye Res. *87*, 415–426.

Hofer, H., Carroll, J., Neitz, J., Neitz, M., and Williams, D.R. (2005). Organization of the human trichromatic cone mosaic. J. Neurosci. *25*, 9669–9679.

Hornan, D.M., Peirson, S.N., Hardcastle, A.J., Molday, R.S., Cheetham, M.E., and Webster, A.R. (2007). Novel retinal and cone photoreceptor transcripts revealed by human macular expression profiling. Invest. Ophthalmol. Vis. Sci. *48*, 5388–5396.

Kaewkhaw, R., Kaya, K.D., Brooks, M., Homma, K., Zou, J., Chaitankar, V., Rao, M., and Swaroop, A. (2015). Transcriptome dynamics of developing photoreceptors in three-dimensional retina cultures recapitulates temporal sequence of human cone and rod differentiation revealing cell surface markers and gene networks. Stem Cells *33*, 3504–3518.

Kim, J.W., Yang, H.J., Oel, A.P., Brooks, M.J., Jia, L., Plachetzki, D.C., Li, W., Allison, W.T., and Swaroop, A. (2016). Recruitment of rod photoreceptors from short-wavelength-sensitive cones during the evolution of nocturnal vision in mammals. Dev. Cell *37*, 520–532.

Komaromy, A.M., Alexander, J.J., Cooper, A.E., Chiodo, V.A., Glushakova, L.G., Acland, G.M., Hauswirth, W.W., and Aguirre, G.D. (2008). Targeting gene expression to cones with human cone opsin promoters in recombinant AAV. Gene Ther. *15*, 1049–1055.

Lakowski, J., Gonzalez-Cordero, A., West, E.L., Han, Y.T., Welby, E., Naeem, A., Blackford, S.J., Bainbridge, J.W., Pearson, R.A., Ali, R.R., and Sowden, J.C. (2015). Transplantation of photoreceptor precursors isolated via a cell surface biomarker panel from embryonic stem cell-derived self-forming retina. Stem Cells *33*, 2469–2482.

Lakowski, J., Han, Y.T., Pearson, R.A., Gonzalez-Cordero, A., West, E.L., Gualdoni, S., Barber, A.C., Hubank, M., Ali, R.R., and Sowden, J.C. (2011). Effective transplantation of photoreceptor precursor cells selected via cell surface antigen expression. Stem Cells *29*, 1391–1404.

Lamba, D.A., Gust, J., and Reh, T.A. (2009). Transplantation of human embryonic stem cell-derived photoreceptors restores some visual function in Crx-deficient mice. Cell Stem Cell *4*, 73–79.

Li, M., Jia, C., Kazmierkiewicz, K.L., Bowman, A.S., Tian, L., Liu, Y., Gupta, N.A., Gudiseva, H.V., Yee, S.S., Kim, M., et al. (2014). Comprehensive analysis of gene expression in human retina and supporting tissues. Hum. Mol. Genet. *23*, 4001–4014.

Li, Q., Timmers, A.M., Guy, J., Pang, J., and Hauswirth, W.W. (2008). Cone-specific expression using a human red opsin promoter in recombinant AAV. Vision Res *48*, 332–338.

Lun, A.T., Mccarthy, D.J., and Marioni, J.C. (2016). A step-by-step workflow for low-level analysis of single-cell RNA-seq data with Bioconductor. F1000Res. *5*, 2122.

MacLaren, R.E., Pearson, R.A., Macneil, A., Douglas, R.H., Salt, T.E., Akimoto, M., Swaroop, A., Sowden, J.C., and Ali, R.R. (2006). Retinal repair by transplantation of photoreceptor precursors. Nature *444*, 203–207.

Meyer, J.S., Shearer, R.L., Capowski, E.E., Wright, L.S., Wallace, K.A., Mcmillan, E.L., Zhang, S.C., and Gamm, D.M. (2009). Modeling early retinal development with human embryonic and induced pluripotent stem cells. Proc. Natl. Acad. Sci. USA *106*, 16698–16703.

Nakano, T., Ando, S., Takata, N., Kawada, M., Muguruma, K., Sekiguchi, K., Saito, K., Yonemura, S., Eiraku, M., and Sasai, Y. (2012). Self-formation of optic cups and storable stratified neural retina from human ESCs. Cell Stem Cell *10*, 771–785.

Nathans, J., Davenport, C.M., Maumenee, I.H., Lewis, R.A., Hejtmancik, J.F., Litt, M., Lovrien, E., Weleber, R., Bachynski, B., Zwas, F., et al. (1989). Molecular genetics of human blue cone monochromacy. Science *245*, 831–838.

Nathans, J., Thomas, D., and Hogness, D.S. (1986). Molecular genetics of human color vision: the genes encoding blue, green, and red pigments. Science *232*, 193–202.

Ng, L., Hurley, J.B., Dierks, B., Srinivas, M., Salto, C., Vennstrom, B., Reh, T.A., and Forrest, D. (2001). A thyroid hormone receptor that is required for the development of green cone photoreceptors. Nat. Genet. *27*, 94–98.

Pearson, R.A., Barber, A.C., Rizzi, M., Hippert, C., Xue, T., West, E.L., Duran, Y., Smith, A.J., Chuang, J.Z., Azam, S.A., et al. (2012). Restoration of vision after transplantation of photoreceptors. Nature *485*, 99–103.

Pearson, R.A., Gonzalez-Cordero, A., West, E.L., Ribeiro, J.R., Aghaizu, N., Goh, D., Sampson, R.D., Georgiadis, A., Waldron, P.V., Duran, Y., et al. (2016). Donor and host photoreceptors engage in material transfer following transplantation of post-mitotic photoreceptor precursors. Nat. Commun. *7*, 13029.

Reichman, S., Terray, A., Slembrouck, A., Nanteau, C., Orieux, G., Habeler, W., Nandrot, E.F., Sahel, J.A., Monville, C., and Goureau, O. (2014). From confluent human iPS cells to self-forming neural retina and retinal pigmented epithelium. Proc. Natl. Acad. Sci. USA *111*, 8518–8523.

Roberts, M.R., Hendrickson, A., Mcguire, C.R., and Reh, T.A. (2005). Retinoid X receptor (gamma) is necessary to establish the S-opsin gradient in cone photoreceptors of the developing mouse retina. Invest. Ophthalmol. Vis. Sci. *46*, 2897–2904.

Roorda, A., and Williams, D.R. (1999). The arrangement of the three cone classes in the living human eye. Nature *397*, 520–522.

Santos-Ferreira, T., Llonch, S., Borsch, O., Postel, K., Haas, J., and Ader, M. (2016a). Retinal transplantation of photoreceptors results in donor-host cytoplasmic exchange. Nat. Commun. *7*, 13028.

Santos-Ferreira, T., Postel, K., Stutzki, H., Kurth, T., Zeck, G., and Ader, M. (2015). Daylight vision repair by cell transplantation. Stem Cells *33*, 79–90.

Santos-Ferreira, T., Volkner, M., Borsch, O., Haas, J., Cimalla, P., Vasudevan, P., Carmeliet, P., Corbeil, D., Michalakis, S., Koch, E., et al. (2016b). Stem cell-derived photoreceptor transplants differentially integrate into mouse models of cone-rod dystrophy. Invest. Ophthalmol. Vis. Sci. *57*, 3509–3520.

Sharon, D., Blackshaw, S., Cepko, C.L., and Dryja, T.P. (2002). Profile of the genes expressed in the human peripheral retina, macula, and retinal pigment epithelium determined through serial analysis of gene expression (SAGE). Proc. Natl. Acad. Sci. USA *99*, 315–320.

Siegert, S., Scherf, B.G., Del Punta, K., Didkovsky, N., Heintz, N., and Roska, B. (2009). Genetic address book for retinal cell types. Nat. Neurosci. *12*, 1197–1204.

Srinivas, M., Ng, L., Liu, H., Jia, L., and Forrest, D. (2006). Activation of the blue opsin gene in cone photoreceptor development by retinoid-related orphan receptor beta. Mol. Endocrinol. *20*, 1728–1741.

Suzuki, S.C., Bleckert, A., Williams, P.R., Takechi, M., Kawamura, S., and Wong, R.O. (2013). Cone photoreceptor types in zebrafish are generated by symmetric terminal divisions of dedicated precursors. Proc. Natl. Acad. Sci. USA *110*, 15109–15114.

Takahashi, K., Tanabe, K., Ohnuki, M., Narita, M., Ichisaka, T., Tomoda, K., and Yamanaka, S. (2007). Induction of pluripotent stem cells from adult human fibroblasts by defined factors. Cell *131*, 861–872.

Tucker, B.A., Park, I.H., Qi, S.D., Klassen, H.J., Jiang, C., Yao, J., Redenti, S., Daley, G.Q., and Young, M.J. (2011). Transplantation of adult mouse iPS cell-derived photoreceptor precursors restores retinal structure and function in degenerative mice. PLoS One *6*, e18992.

Wang, Y., Macke, J.P., Merbs, S.L., Zack, D.J., Klaunberg, B., Bennett, J., Gearhart, J., and Nathans, J. (1992). A locus control region adjacent to the human red and green visual pigment genes. Neuron *9*, 429–440.

Whitmore, S.S., Wagner, A.H., Deluca, A.P., Drack, A.V., Stone, E.M., Tucker, B.A., Zeng, S., Braun, T.A., Mullins, R.F., and Scheetz, T.E. (2014). Transcriptomic analysis across nasal, temporal, and macular regions of human neural retina and RPE/choroid by RNA-Seq. Exp. Eye Res. *129*, 93–106.

Xiao, M., and Hendrickson, A. (2000). Spatial and temporal expression of short, long/medium, or both opsins in human fetal cones. J. Comp. Neurol. *425*, 545–559.

Zhong, X., Gutierrez, C., Xue, T., Hampton, C., Vergara, M.N., Cao, L.H., Peters, A., Park, T.S., Zambidis, E.T., Meyer, J.S., et al. (2014). Generation of three-dimensional retinal tissue with functional photoreceptors from human iPSCs. Nat. Commun. *5*, 4047.

Stem Cell Reports, Volume *9*

Supplemental Information

Isolation and Comparative Transcriptome Analysis of Human Fetal and iPSC-Derived Cone Photoreceptor Cells

Emily Welby, Jorn Lakowski, Valentina Di Foggia, Dimitri Budinger, Anai Gonzalez-Cordero, Aaron T.L. Lun, Michael Epstein, Aara Patel, Elisa Cuevas, Kamil Kruczek, Arifa Naeem, Federico Minneci, Mike Hubank, David T. Jones, John C. Marioni, Robin R. Ali, and Jane C. Sowden

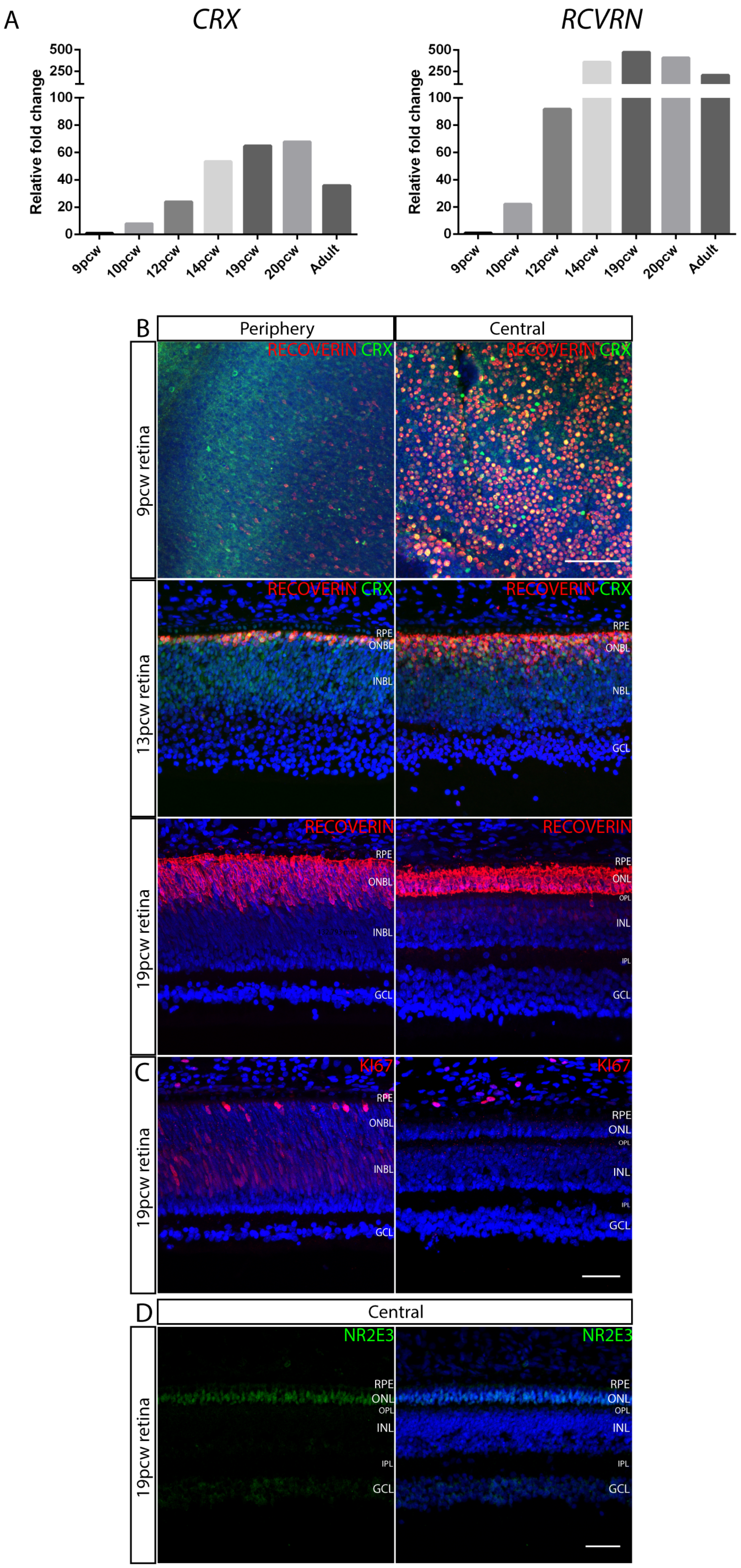

A
CRX
RCVRN
Relative fold change
500
250
100
80
60
40
20
0
9pcw
10pcw
12pcw
14pcw
19pcw
20pcw
Adult
B
Periphery
Central
9pcw retina
13pcw retina
19pcw retina
RECOVERIN CRX
RECOVERIN
RPE
ONBL
INBL
NBL
ONL
OPL
INL
IPL
GCL
C
KI67
D
NR2E3

Figure S1. Photoreceptor marker expression in the human peripheral and central retina (related to Figure 1).

(A) qRT PCR analysis of pan photoreceptor markers, cone-rod homeobox gene (*CRX*) and recoverin (*RCVRN*) in foetal (9pcw-20pcw) and adult retinal samples (n = 1 per timepoint). **(B)** RECOVERIN and CRX protein expression in the peripheral and central region of the developing retina (9pcw-19pcw). **(C)** Differences in retinal maturity are highlighted by KI67 immunostaining, present in the peripheral retina but absent in the central retina. **(D)** Rod marker, NR2E3, expression in the peripheral and central 19pcw retina. Wholemount image scale bar: 100µm; tissue section scale bar: 50µm. RPE, retinal pigment epithelium; ONBL, outer neuroblastic layer; INBL, inner neuroblastic layer; ONL, outer nuclear layer; OPL, outer plexiform layer; INL, inner nuclear layer; IPL, inner plexiform layer; GCL, ganglion cell layer.

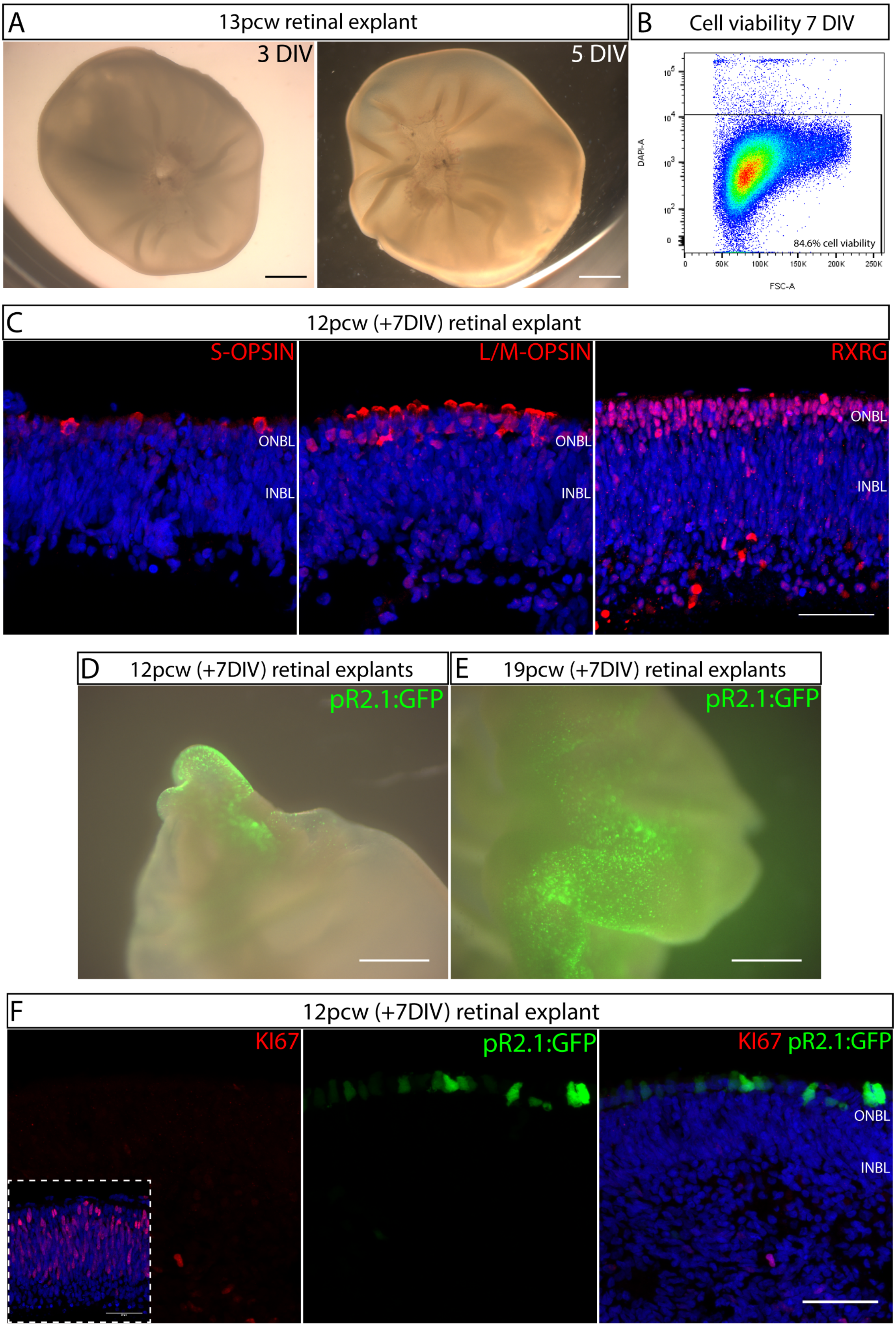

G

Marker	No. of GFP+ cells	No. of marker+ cells	No. of GFP+/marker+ cells	Percentage
L/M-OPSIN	53	93	29	54.7
RXRγ	84	233	84	100
S-OPSIN	44	15	0	0
NR2E3	55	143	0	0

Figure S2. AAV2/9.pR2.1:GFP reporter expression in human foetal retinal explant culture system (related to Figure 2).

(A) Brightfield wholemount images showing 13pcw retinal explant at 3 and 5 days *in vitro* (DIV) using a free floating culture method. **(B)** Dissociation and flow cytometry analysis of the explant on 7 DIV shows a high level of cell viability. **(C)** Representative images of 12pcw (+7DIV) retinal explant showing preserved cone photoreceptor marker staining (S-OPSIN, L/M-OPSIN, RXRG) and retinal integrity. Wholemount images showing pR2.1:GFP+ signal within cultured 12pcw **(D)** and 19pcw **(E)** retinal explants. **(F)** Sections of a 12pcw (+7DIV) retinal explant showing the absence of KI67 expression within pR2.1:GFP+ cells. Insert shows KI67 positive control for antibody. Scale bar for wholemount images of foetal retinal explants: 1mm; tissue section scale bar: 50µm. ONBL, outer neuroblastic layer; INBL, inner neuroblastic layer. **(G)** Counting of the marker + and GFP+ cells from immunofluorescence data in Figure 1L-O. Three sections of a 12pcw retinal explant transduced with the pR2.1:GFP reporter were counted for each immunostaining experiment (1 field of view per section where GFP+ cells and marker + cells could be seen). 0% of GFP+ cells co-labelled with NR2E3 (55 GFP+ cells counted in total), 0% of cells co-labelled with S-OPSIN (44 GFP+ cells counted in total), 100% of cells co-labelled with RXRG (84 GFP+/RXRG+ cells; 84 GFP+ cells counted in total) and 54.7% of cell co-labelled with L/M-OPSIN (29 GFP+/L/M-OPSIN+ cells; 53 GFP+ cells counted in total).

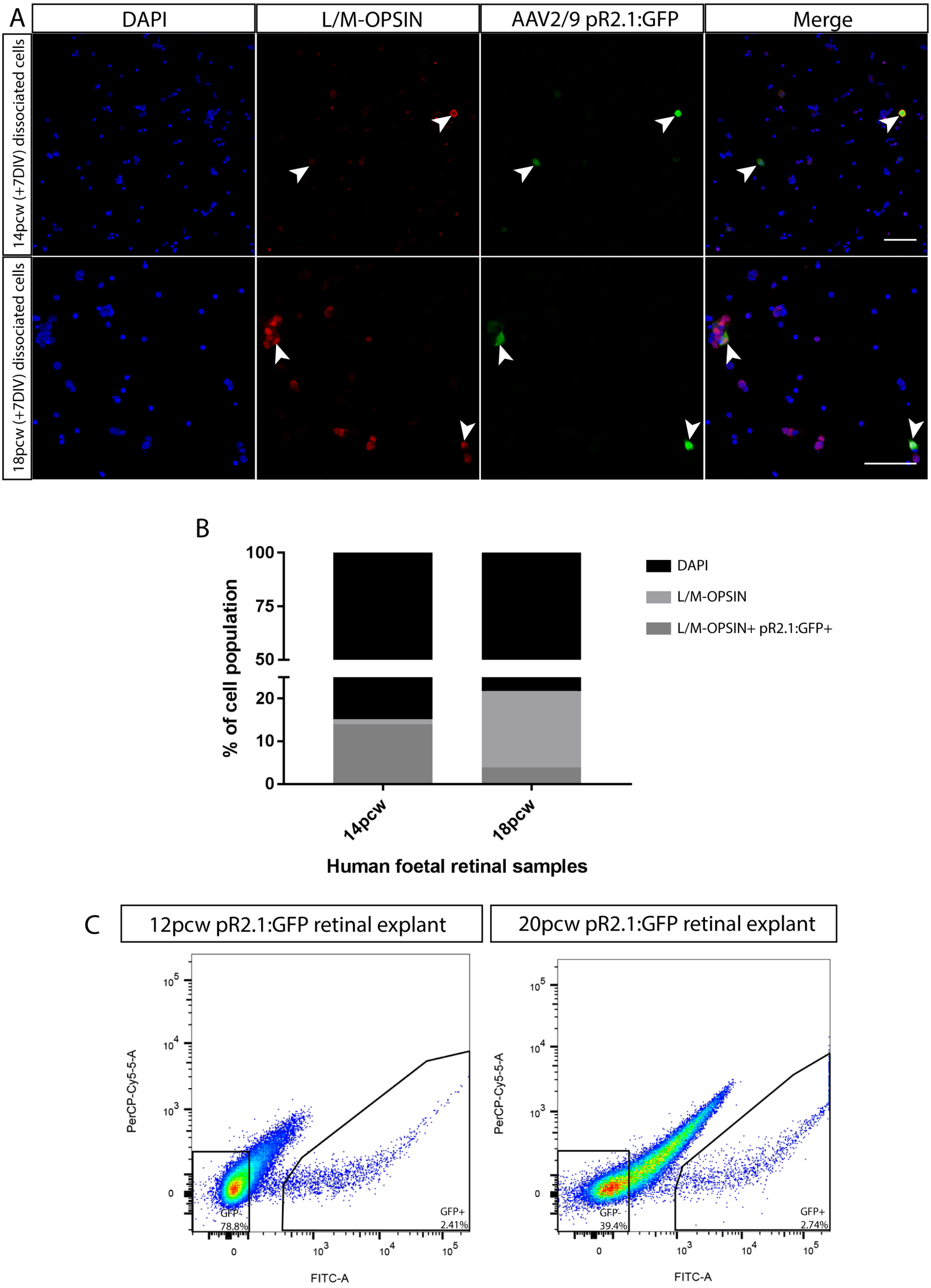

A
DAPI
L/M-OPSIN
AAV2/9 pR2.1:GFP
Merge
14pcw (+7DIV) dissociated cells
18pcw (+7DIV) dissociated cells
B
% of cell population
100
75
50
20
10
0
14pcw
18pcw
Human foetal retinal samples
DAPI
L/M-OPSIN
L/M-OPSIN+ pR2.1:GFP+
C
12pcw pR2.1:GFP retinal explant
20pcw pR2.1:GFP retinal explant
PerCP-Cy5-5-A
FITC-A
GFP-
78.8%
GFP+
2.41%
GFP-
39.4%
GFP+
2.74%

Figure S3. Efficiency of AAV2/9.pR2.1:GFP reporter and pR.2.1:GFP+ cell isolation via FACS (related to Figure 1 and 2).

(A) L/M-OPSIN immunostaining on dissociated pR2.1:GFP+ 14pcw and 18pcw (+7DIV) retinal explants show GFP+ cells co-label with L/M-OPSIN expression (white arrows). **(B)** Counting of these cells reveals the number of L/M-OPSIN+ cells and pR2.1:GFP+ cells in each sample. In test samples, reporter labelling efficiencies of 14% and 4% were achieved in 14pcw (30 GFP+/214 L/M-OPSIN+ cells) and 18pcw (9 GFP+/230 L/M-OPSIN+ cells) retinae, respectively. **(C)** Representative FACS traces of foetal pR2.1:GFP+ retinal explants showing gates used to collect GFP+ and GFP- cells for RNA seq.

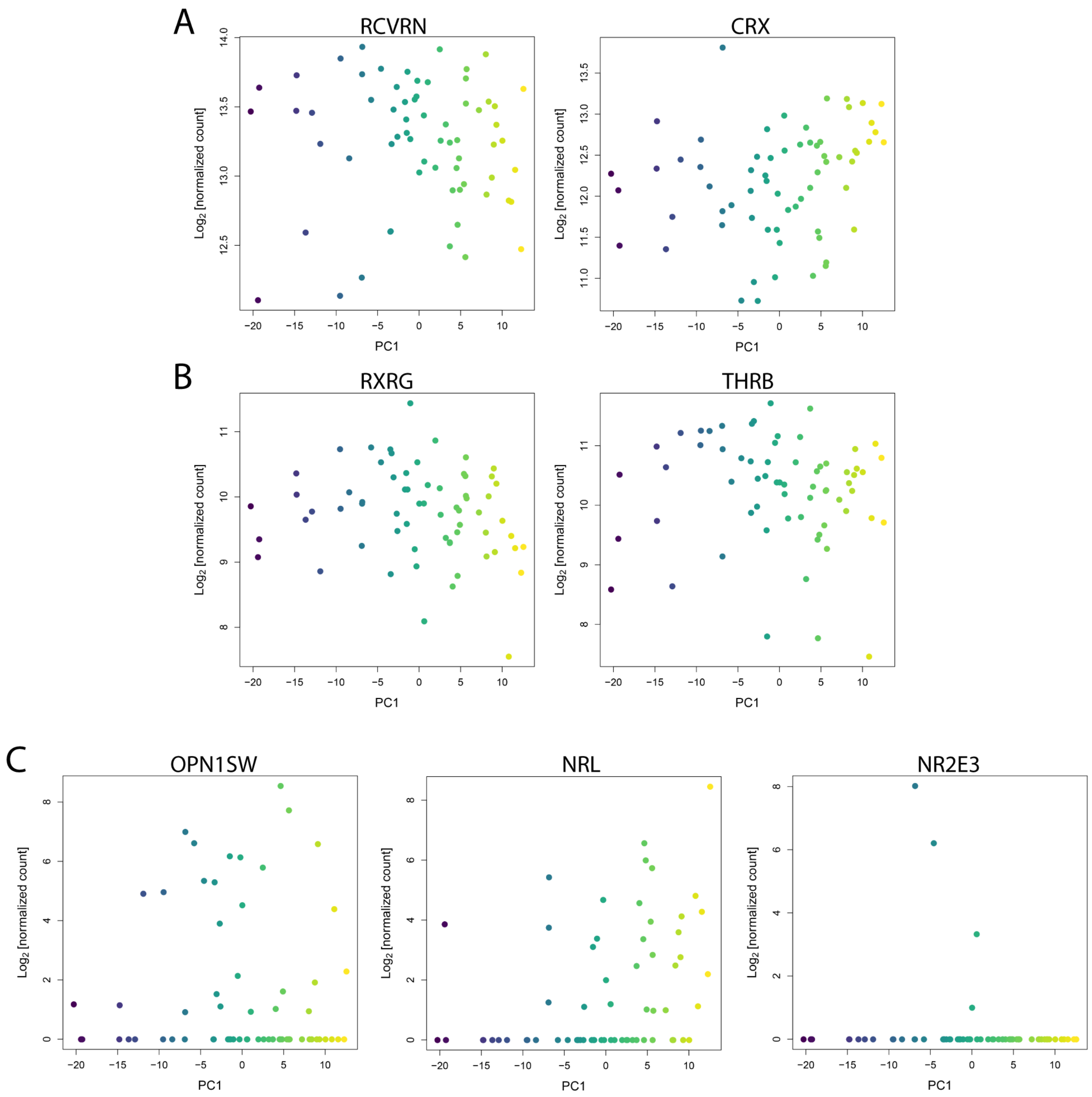
A
RCVRN
CRX
B
RXRG
THRB
C
OPN1SW
NRL
NR2E3
Log2 [normalized count]
PC1

Figure S4. Photoreceptor gene expression profile across single AAV2/9 pR2.1:GFP labelled cells (related to Figure 3).

Gene expression (log2 normalised counts) profiles of pan photoreceptor genes (*RCVRN* and *CRX*; **A)**, cone photoreceptor genes (*RXRG* and *THRB*; **B)**, S-cone (*OPN1SW*) and rod photoreceptor genes (*NRL* and *NR2E3*; **C)** across individual human foetal 15pcw pR2.1:GFP+ cells correlated to PC1. Each dot represents a cell and the cell position across PC1 (x-axis) is determined by its differential gene expression, which is indicated by colour (blue = naïve, yellow = mature). The expression level of each gene in each cell is shown on the y-axis.

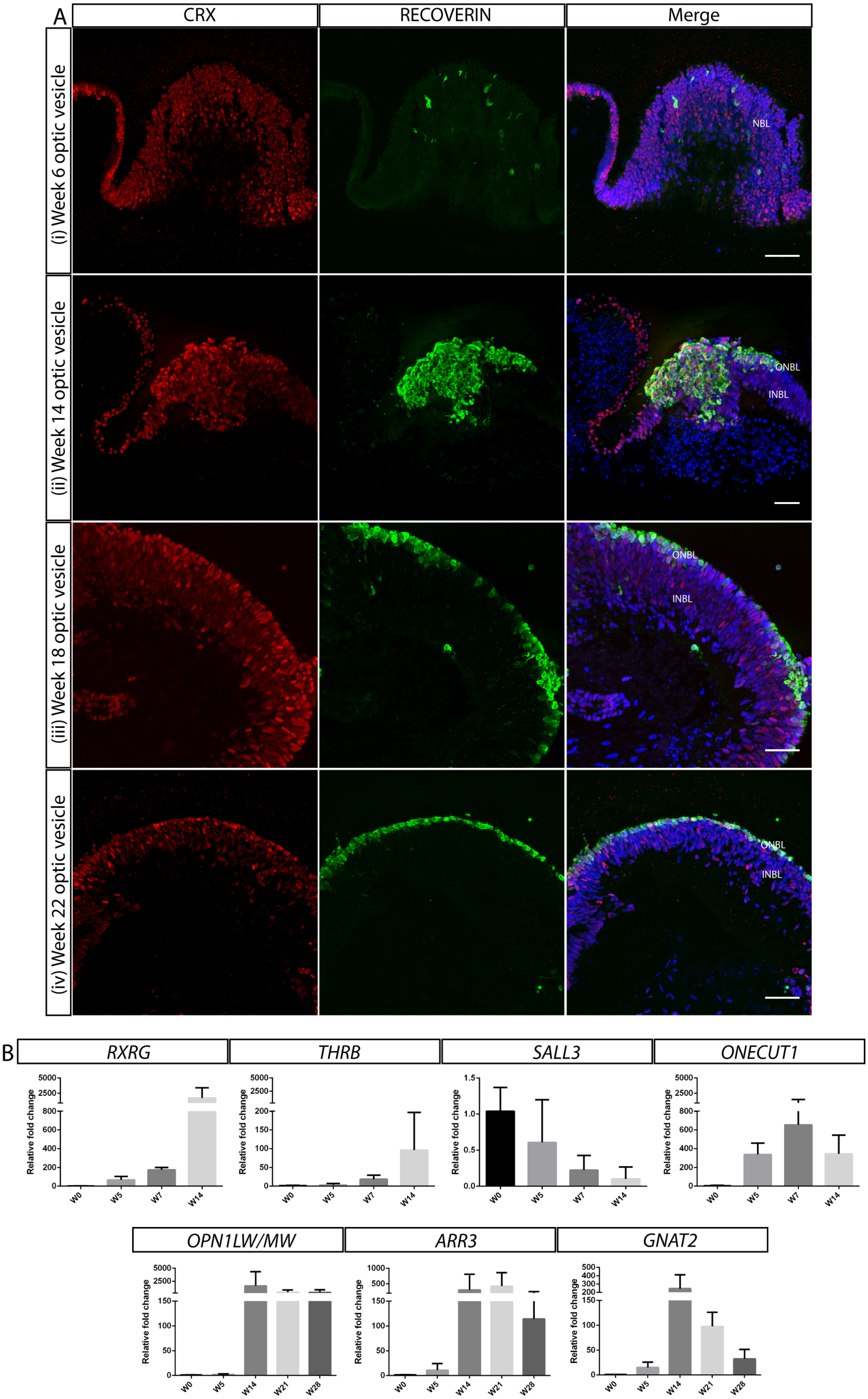
A
CRX
RECOVERIN
Merge
(i) Week 6 optic vesicle
NBL
(ii) Week 14 optic vesicle
ONBL
INBL
(iii) Week 18 optic vesicle
ONBL
INBL
(iv) Week 22 optic vesicle
ONBL
INBL
B
RXRG
THRB
SALL3
ONECUT1
Relative fold change
W0
W5
W7
W14
OPN1LW/MW
ARR3
GNAT2
W21
W28

Figure S5. Photoreceptor differentiation from iPSCs using 3D retinal organoid culture system (related to Figure 4)

(A i-iv) Protein expression of CRX and RECOVERIN was detected within iPSC-derived optic vesicle structures from week 6 through to week 22 of differentiation. **(B)** qRT PCR analysis of cone photoreceptor marker transcripts during retinal differentiation culture; n = 3 per timepoint. Error bars represent ± SD. Tissue section scalebar: 50µm. ONBL, outer neuroblastic layer; INBL, inner neuroblastic layer.

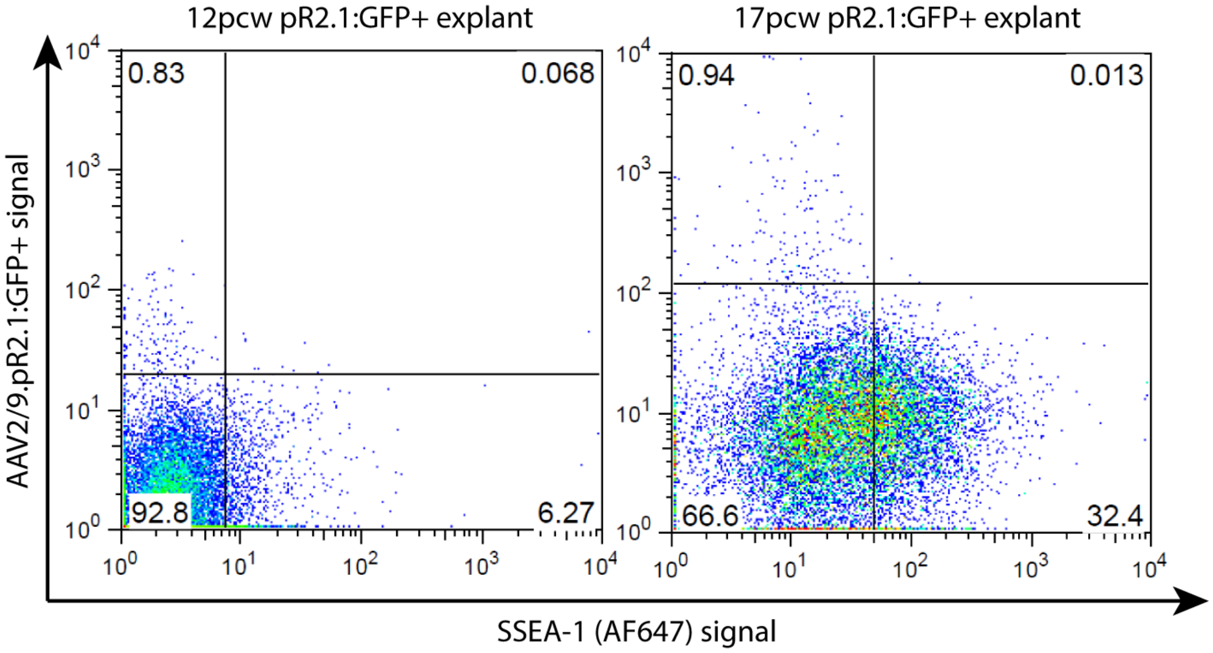

12pcw pR2.1:GFP+ explant
17pcw pR2.1:GFP+ explant
0.83
0.068
92.8
6.27
0.94
0.013
66.6
32.4
AAV2/9.pR2.1:GFP+ signal
SSEA-1 (AF647) signal

Figure S6. SSEA-1 expression in the human foetal retina (related to Figure 5)

Flow cytometry traces of pR2.1:GFP+ cells vs SSEA-1+ cells in the 12pcw and 17pcw foetal retina. Cells co-labelling for SSEA-1 and pR2.1:GFP+ cells were not detected at either timepoint in the foetal retinal samples, suggesting SSEA-1 is not expressed by pR2.1:GFP+ cone cells. In the 17pcw sample, SSEA-1 labelled 32.4% of GFP- cells.

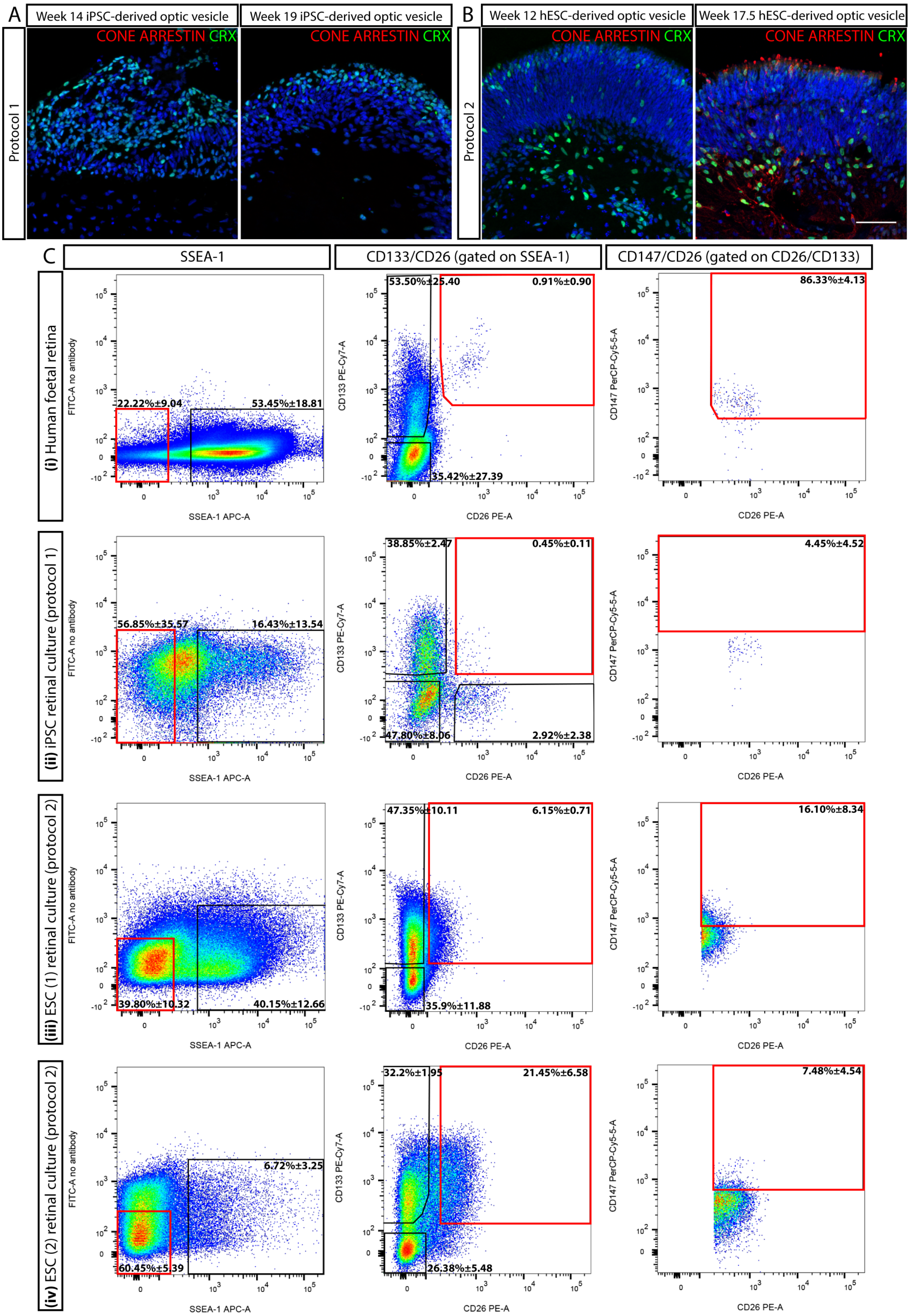
A
Week 14 iPSC-derived optic vesicle
Week 19 iPSC-derived optic vesicle
CONE ARRESTIN CRX
Protocol 1
B
Week 12 hESC-derived optic vesicle
Week 17.5 hESC-derived optic vesicle
Protocol 2
C
SSEA-1
CD133/CD26 (gated on SSEA-1)
CD147/CD26 (gated on CD26/CD133)
(i) Human foetal retina
22.22%±9.04
53.45%±18.81
53.50%±25.40
0.91%±0.90
35.42%±27.39
86.33%±4.13
(ii) iPSC retinal culture (protocol 1)
56.85%±35.57
16.43%±13.54
38.85%±2.47
0.45%±0.11
47.80%±8.06
2.92%±2.38
4.45%±4.52
(iii) ESC (1) retinal culture (protocol 2)
39.80%±10.32
40.15%±12.66
47.35%±10.11
6.15%±0.71
35.9%±11.88
16.10%±8.34
(iv) ESC (2) retinal culture (protocol 2)
60.45%±5.39
6.72%±3.25
32.2%±1.95
21.45%±6.58
26.38%±5.48
7.48%±4.54
FITC-A no antibody
SSEA-1 APC-A
CD133 PE-Cy7-A
CD26 PE-A
CD147 PerCP-Cy5-5-A

Figure S7. Cone differentiation and cone CD marker FAC-sorting from PSCs using retinal differentiation protocols (related to Figure 6)

(**A-B**) Histological sections of iPSC-derived optic vesicles generated using (**A**) the adapted Meyer et al., 2009 protocol (protocol 1) used for RNA seq analysis (refer to Figure 4) and (**B**) ESC-derived optic vesicles generated using the new protocol developed by Gonzalez-Cordero et al., 2017 (protocol 2; H9). Immunohistological analysis with CONE ARRESTIN/CRX antibodies detected CONE ARRESTIN by week 17.5 of differentiation within the ESC-derived vesicles **(B)**. Scalebar of retinal tissue section: 50µm.
(**C**) Representative flow cytometry traces showing the cell populations obtained when applying the cone CD marker combination (SSEA-1/CD133/CD26/CD147) onto **(C i)** the human foetal retina (example shows 17pcw foetal retina), **(C ii)** Week 20 iPSC-derived retinal differentiation cultures generated using protocol 1 (adapted Meyer et al., 2009 protocol), **(C iii)** Week 17-18 ESC (mShef10; ESC 1)-derived retinal differentiation cultures generated using protocol 2 (developed by Gonzalez-Cordero et al., 2017), **(C iv)** Week 17-18 ESC (H9; ESC 2)-derived retinal differentiation cultures generated using protocol 2. The foetal retina flow cytometry traces were used as a baseline reference for cone cell enrichment **(C i)**. Retinal differentiation of ESC lines with protocol 2 **(C iii and C iv)** showed SSEA1-CD133+CD26+CD147+ cell populations most similar to the foetal traces. Cell percentage is given within each gate; mean represents ±SD, n = 2-3.

SUPPLEMENTAL EXPERIMENTAL PROCEDURES

PSC maintenance and retinal differentiation

iPSCs were cultured on irradiated mouse embryonic fibroblast layer (GlobalStem) in knockout serum replacement medium (KSR; DMEM/F12 1:1 (Gibo), 20% Knockout Serum Replacement (KOSR; Life Technologies), 1% MEM non-essential amino acids (Life Technologies), 2mM L-glutamine (Life Technologies), 0.007% 2-mercaptoethanol (Life Technologies)) with 4ng/ml fibroblastic growth factor (FGF; Peprotech). H9 ESCs (Wicell) were cultured in feeder free conditions in E8 (Thermo Fisher) and geltrex coated 6 well plates. mShef10 ESCs (Centre for Stem Cell Biology, The University of Sheffield) were cultured in Nutristem (Geneflow) on Laminin-521 (BioLamina) coated 6 well plates. The retinal differentiation protocol for transcriptome profiling of iPSC-derived cones was based on Meyer et al., 2009 (Protocol 1). iPSC colonies were enzymatically lifted using 0.1% w/v dispase-DMEM/F12 (Life Technologies) on Day 0 and transfer into T25 flasks containing KSR medium without FGF to induce the formation of embryoid bodies (EBs). Medium was changed daily before substituting into neural induction medium (NIM) containing DMEM/F12 1:1 (Gibco), 1% MEM non-essential amino acids (Life Technologies), 1% N2 supplement (Gibco), 0.1% heparin (Sigma) on Day 4. To promote neural rosette formation, EBs were transferred onto 30% v/v laminin-DMEM/F12 (Sigma) coated plates in NIM on Day 6; neural rosettes appeared by differentiation day 8/9. Cultures were then fed every 2 days with NIM until Day 16, where the media was substituted for retinal differentiation media (RDM; 70% DMEM (Gibco), 30% F12 (Gibco) and 2% B27 supplement (Gibco)). Optic vesicle structure was visible from Day 18-20 onwards and media was changed every 2-3 days. 10% FBS (Gibco) was added to cultures at week 14 for late stage cultures. For CD marker FAC-sorting experiments, iPSCs were differentiated using protocol 1 (based on Meyer et al., 2009) and mShef10 and H9 lines were differentiated using the protocol as described in (Gonzalez-Cordero et al., 2017).

Human foetal retinal explant culture

Foetal retinae were isolated under sterile conditions in DMEM to remove surrounding ocular tissue using stainless steel 8mm blade curved micro scissors and 0.1mm straight forceps (World Precision Instruments). Intact foetal retina were cultured free floating in 12 or 24 well plates with retinal differentiation media (RDM) containing DMEM-F12 (Gibco), 1% Glutamax (Gibco), 1% N2 and 1% B27 neural supplements (Gibco), 10% FBS (Invitrogen) and 1.5% penicillin/streptomycin (Invitrogen). Cell culture media was changed every 2 days.

AAV2/9.pR2.1:GFP production and application

293T cells were cultured in D10 medium containing DMEM+Glutamax (Life Technologies), 10% FBS (Life Technologies) and 1% pencillin/streptomycin (Life Technologies) in 150cm plates (20 plates required for one batch of virus) and grown to 80% confluency. 100µg pd10/pR2.1.L/Mopsin-eGFP construct, 30µg pHGT1 helper vector, 100µg AAV2/9 caspid were added to 52.5ml DMEM and 1.2ml PEI polyethylenimine (PEI) prior to addition onto the 293T cells which facilitated transfection, before harvesting the cells after 72 hours using a cell scraper and collecting in TD buffer and performing four freeze (-80°C), thaw (37°C), vortex cycles for viral release. The pR2.1.L/M-opsin promoter was originally generated by the Jeremy Nathans group (Wang et al., 1992). Virus lysate underwent benzonase treatment (50 units; Sigma) and was prepared for purification through multiple centrifugation steps and sequential filtering with 5µm, 0.45µm and 0.22µm PES membranes. Virus was purified via ion exchange fast protein liquid chromatography using AKTA Prime (GE Healthcare) and anionic sephacryl S300 and POROS 50HQ columns prior to elution with an increasing salt gradient. The elution was concentrated using a Vivaspin 4 column (Sartorius) to a final volume ranging between 250-400µl. Viral genomic titer was determined through qRT PCR and the virus titer used in this study ranged from $1.48x10^{13}$-$1.01x10^{14}$. Virus was added to foetal retinal explant and iPSC-derived retinal differentiation cultures at a MOI of 40,000-45,000.

Fluorescence-activated cell sorting (FACS) of human retinal cells

Human foetal and PSC-derived retinal tissue were enzymatically dissociated into single cells using the papain dissociation system (Worthington Biochemical, Lorne Laboratories, UK) according to manufacturer's protocol. All samples were passed through 70µm cell strainer prior to FACS. For FAC-sorting of pR2.1.GFP+ and GFP- retinal material, cells were resuspended in blocking solution (BS; 1% (w/v) BSA/PBS) with the addition of DAPI (2µl; Sigma) before sorting to allow the determination of live cell population. For cone CD marker sorting, cells were counted using a haemocytometer and resuspended in BS at a concentration of $1x10^6$ cells per 100ul. After incubating cell for 1 hour on ice, conjugated antibodies (APC-conjugated SSEA-1, clone REA321, Miltenyi Biotec, Cat no. 130-104-936; PE- conjugated CD26, clone M-A261, BD Biosciences, Cat no. 555437; PE- Vio770-conjugated CD133, clone 293C3, Miltenyi Biotec, Cat no. 130-104-117; PerCP-Cy5.5-conjugated CD147, clone HIM6, BD Biosciences, Cat no. 562554) or isotype controls were added to cells using the manufacturer's recommendations and incubated for a further hour on ice in the dark. Cells were washed in PBS and resuspended in BS for sorting. The BD FACSARIA III was used to isolate cells which were collected into

50% (v/v) FBS/DMEM media. FITC-A vs PerCP-Cy5-5A channels were used to detect GFP+ signal, whereas the according channels matching the fluorophores of the conjugated CD marker antibodies were used to detect positive signal. Isotype and unstained controls were used to set gates and apply necessary compensation. Post-sorting, cells were centrifuged at 300 x g for 15 minutes at 4°C; cell pellets for RNA extraction were immediately snap frozen on dry ice, whereas cells for immunocytochemistry were plated out on to pre-coated poly-L-lysine (Sigma) and laminin (1:30; Sigma) chamber slides. For FACS analysis of foetal and ESC-derived retinal samples, 4 independent samples were analysed for each CD marker combination and at least 50 cells were counted for each FAC-sorted cell population. FlowJo flow cytometry software was used for FACS trace visualisation and analysis.

Immunocytochemistry on dissociated retinal cells

Dissociated retinal cells on chamber slides were fixed in 4% (w/v) PFA/PBS for 5 minutes at 37°C prior to a further 15 minutes incubation in 2% (w/v) PFA/ 30% (w/v) sucrose/PBS at room temperature (RT). Cells were washed three times in PBS and incubated for 1 hour at RT in BS (10% (v/v) foetal bovine serum (FBS)/PBS (Gibo), 1% (w/v) bovine serum albumin/PBS) with 0.1% (v/v) Triton X-100. Primary antibodies (L/M-opsin, Millipore, Cat no. AB5405, 1:400, CRX, Abnova, H00001406-M02, 1:800; Cone arrestin, NovusBio, NBP1-19629, 1:100) were added on to cells and incubated for 1 hour at RT. Cells were washed with three times in PBS and incubated for a further hour with secondary antibodies (Alexa Fluor 594 Goat anti-rabbit IgG (Cat no. A11032) or Alexa Fluro 488 Goat anti-mouse IgG (Cat no. A1080), Invitrogen, 1:800). The same procedure was conducted for CD marker immunocytochemistry (L/M-opsin, Millipore, Cat no. AB5405, 1:400, Cone arrestin, NovusBio, NBP1-19629, 1:100, PE- conjugated CD26, 1:500, BD Biosciences, Cat no. 555437; PE- Vio770-conjugated CD133, 1:500, Miltenyi Biotec, Cat no. 130-104-117; PerCP-Cy5.5-conjugated CD147, clone 1:500, BD Biosciences, Cat no. 562554) on dissociated cells, with the removal of Triton X-100 from BS. DAPI incubation and mounting procedure was performed as described in histology and immunohistochemistry section.

Human BD lyoplate screening panel protocol

Retinal samples previously transfected with AAV2/9.pR2.1:GFP reporter were dissociated using the papain method as described in the FACS methods section and the protocol for the human BD Lyoplate™ Screening Panels was followed accordingly to manufacturer's instructions. Briefly, cells were resuspended in BD Pharmingen Stain Buffer with 5mM EDTA to a cell concentration of 25k-1x10^6 cells/100µl and aliquoted into

3x BD Falcon™ round bottom 96 well plates (100µl per well). Primary antibodies of the BD Lyoplate™ Screening Panels were reconstituted in 1x PBS (110µl per well) and 20µl of each antibody was then added to the cells and incubated for 30 minutes on ice. Primary antibody was omitted for negative control and wells were allocated for IgG/IgM positive controls. Cells were then washed in stain buffer, resuspended in 100µl of secondary antibody (Alexa Fluor 647) solution before incubating on ice for 30 minutes in the dark. Subsequently, cells were washed and centrifuged before being resuspended in 150µl of BD Pharmingen Stain Buffer + EDTA. Samples were then analysed using the BD FACSCabilur (BD Biosciences). 15,000-20,000 events were collected per well and results were analysed using the FlowJo software.

Histology and Immunohistochemistry

Human foetal eyes were fixed in 4% (w/v) PFA/PBS overnight at 4°C; retinal explants/wholemounts and PSC-derived retinal differentiation samples were fixed for 30 minutes at 4°C. For embedding, samples were washed three times in PBS and equilibrated in 30% (w/v) sucrose/PBS solution for cryo-protection at RT; once sunk, samples were then orientated and placed into foil moulds containing optimal cutting temperature (OCT) compound, before freezing in methylbutane-dry ice slurry. Tissue sections were cut to 14-16µm thickness using the Leica CM1900 UV cryostat and collected on SuperfrostTM plus glass slides (VWR). For immunohistochemistry, retinal sections were washed in PBS for 13 minutes at 37°C to remove the OCT compound (step excluded for wholemount staining) and incubated in BS (10% (v/v) foetal bovine serum (FBS)/PBS (Gibo), 1% (w/v) bovine serum albumin/PBS) with 0.1% (v/v) Triton X-100 for 1 hour at RT. Sections were subsequently incubated with primary antibody for 1 hour at RT or overnight at 4°C. Primary antibodies used in this study include Recoverin, Millipore, Cat no. AB5585, 1:1000; CRX, Abnova, Cat no. H00001406-M02, 1:800; L/M-opsin, Millipore, Cat no. AB5405, 1:500; S-opsin, Millipore, Cat No. AB5407, 1:500; Rxrg, Abcam, Cat no. AB15518, 1:300; Gnat2, Santa Cruz, Cat no. sc-390, 1:300; Ki67, Abcam, Cat no. AB15580, 1:300; Nr2e3, R&D Systems, Cat no. 2ZH7223H, 1:500; Rhodopsin; Upstate, Cat no. 06-770 1:1000; Nrl, R&D Systems, Cat no. AF2945, 1:500, Cone arrestin, NovusBio, Cat no. NBP1-19629 1:100. Primary antibody was omitted for negative controls. Sections were washed three times in 1x PBS before applying the secondary antibody for 1 hour at RT (AlexaFluor 594 Goat anti-rabbit (Cat no. A11037), AlexaFluor 488 Donkey anti-mouse (Cat no. A-21202) , AlexFluor 594 Donkey anti-goat (Cat no A-11058). Invitrogen, 1:800). Sections were washed 3 times with PBS prior to DAPI incubation (1:3000 in PBS) at RT for

5 minutes, allowing for the visualisation of cell nuclei. Sections were washed again in PBS, before applying with Citifluor AF-1 mounting medium, 1.5 coverslips and clear nail polish to seal coverslip.

Microscopy and image processing

Z-28 projection images of retinal sections and wholemount immunostainings were acquired using the Zeiss LSM710 (Zen2009, Zeiss) confocal microscope. Z-28 projection and at least 2x2 tile scan images were acquired for of FAC-sorted cells to be counted. Brightfield and fluorescent images of foetal pR2.1:GFP+ retinal explants were captured using the Leica MZFLIII fluorescence stereomicroscope and the Leica DC500 camera. Fluorescent and brightfield images of stem cell-derived cones were acquired using the inverted Olympus IX71 (Carl Zeiss, Jena, Germany) microscope with a Hamamatsu ORCA-ER digital camera (Hamamatsu Corp., Bridgewater, NJ). Images were processed using Zen2009 (Zeiss), ImageJ and Illustrator CS6 (Adobe). Cell counting was completed using ImageJ software.

RNA isolation and quantitative real-time PCR

Retinal tissue was processed with the mirVana RNA extraction kit (Ambion) according to manufacturer's recommendation. For qRT PCR analysis, RNA samples were treated with DNaseI (Invitrogen) and cDNA was synthesised using SuperScript III Reverse Transcriptase (Invitrogen). cDNA samples were diluted to the same concentration (5ng) with DEPC-treated water. RT qPCR procedure was carried out in 96 well plates with a total reaction volume of 20µl per well (2µl of cDNA sample, 17µl of TaqMan Gene Expression Master Mix (Thermo Fisher) with DEPC-treated water and 1µl of TaqMan gene expression assays (Thermo Fisher). The following gene expression assays were used in this study for detecting transcripts of interest: *RXRG* (Hs00199455_m1), *ONECUT1* (Hs00413554_m1), *THRB2* (AJ20TM1), *SALL3* (Hs00923915_m1), *GNAT2* (Hs00292542_m1), *ARR3* (Hs00182888_m1), *OPN1SW* (Hs00181790_m1), *OPN1LW/MW* (Hs00241039_m1), *RCVRN* (Hs00610056_m1), *CRX* (Hs00230899_m1). The assay used for *OPN1LW/MW* detects transcripts from all L and M-opsin genes. *GADPH* (Hs02758991_g1) was used as an internal control to normalise the expression of candidate genes and sample cDNA was replaced with DEPC-treated water for negative controls. qRT PCRs were performed on a 7500 Real-Time PCR System with the FAM settings according to manufacturer's recommendation. The following PCR cycle was used for all experiments: Stage 1, 1x cycle, 50°C for 2 minutes; Stage 2, 1x cycle, 95°C for 10 minutes; Stage 3, 40x cycles, 95°C for 15 seconds, 60°C for 60 seconds. The

Applied Biosystems 7500 Real Time PCR System software (v2.0.2) was used to assess quality of sample output before exporting and performing data analysis in Microsoft Excel.

Bulk mRNA sequencing and bioinformatics analysis

RNA concentration and quality was assessed on the Agilent Bioanalyzer or Tapestation using a RNA 6000 Pico kit (Agilent Technologies). High quality RNA (RIN:6.70-10) was used for RNA seq. cDNA quality was assessed using Qubit dsDNA HS Assay kit and Qubit 2.0 Fluorometer (ThermoFisher). pR2.1.GFP+ and GFP- FAC-sorted samples for bulk RNA-seq analysis were amplified using the SMART-Seq v4 Ultra Low Input RNA kit (Clontech Laboratories) and cDNA libraries were prepared using the Nextera XT DNA Library Preparation Kit (Illumina), before sequencing libraries using the Illumina NextSeq500 system with a targeted sequencing depth of 17million 43bp paired end per sample. FASTQ files containing raw RNA seq data were aligned using Illumina RNA Seq STAR alignment tool (version 1.1.0) to the reference human genome (GRCh37/hg19), in order to generate BAM files. Quality control was performed pre and post alignment using the RNA-Seq alignment tool in BaseSpace (Illumina). BAM files and a gene annotation file (Homo_sapiens.GRCh37.87.chr.gtf from Ensembl) were uploaded to Galaxy (version 17.01). Aligned reads were assigned to genes and quantified using featureCounts (version 1.4.6.p5 (Liao et al., 2014) to obtain a raw count matrix for each sample. The raw count matrices were loaded into Strand NGS (Strand Genomics), before performing DESeq normalisation for hierarchical clustering analysis (Euclidean similarity measure and Ward's linkage rule used), and data visualisation using Principal Component Analysis (PCA) and making heatmap representations. The raw count matrices were then used as input to the DESeq2 (Love et al., 2014) pipeline using default parameters with the Galaxy version 2.11.38 for the differential gene expression analyses, which uses Wald test statistics and Benjamini-Hochberg correction for multiple testing. log10 adjusted p-values and log2 fold changes generated by these analyses were plotted using R (version 3.2.1 (2015-06-18) to create volcano plots. Genes with total raw count matrices lower than 10 across all samples were removed from analysis. Venn diagrams were constructed using Bioinformatic & Evolutionary Genomics tool (http://bioinformatics.psb.ugent.be/webtools/Venn/). GO analysis was performed using Enrichr (Chen et al., 2013, Kuleshov et al., 2016) and enriched GO terms with a combined score >10 were represented using Revigo (Supek et al., 2011). Accession numbers E-MTAB-6057 can be used to access fastq files of all bulk RNA seq samples on ArrayExpress EMBL-EBI.

Single cell RNA sequencing and bioinformatics analysis

For single cell analysis, pR2.1.GFP+ were isolated via FACS and separated using a medium-sized (10-17µm cell diameter) integrated fluidic circuit (IFC; Fluidigm), using the Fluidigm C1 system. Prior to loading on the IFC, cell viability was assessed using a standard trypan blue exclusion test before resuspending cells at a concentration of 667cells/µl. 3µl total sample volume was loaded onto IFC and cell were imaged via phase-contrast microscopy to check for the single cell status of each chamber. 74 single cells were captured for the experiment and chambers containing debris, dead cells and multiple cells were excluded from the analysis. After collecting cell harvest from the IFC, amplification, cDNA libraries and sequencing was performed using the same bulk RNA seq procedures. A targeted sequencing depth of 2 million 75bp paired end reads per sample was used and External RNA Control Consortium (ERCC) RNA spike-in Mix (ThermoFisher) was added to each cell sample prior to sequencing. After the sequencing, FASTQ files from individual cell samples were aligned to the hg19 build of the human genome with ERCC spike-in sequences (Thermofisher) using subread version 1.5.0-p1 (Liao et al 2013). The featureCounts software (Liao et al 2014) was used to obtain a matrix of gene by cell counts using gene feature files from Ensembl GRCh37 version 82 and ThermoFisher. The count matrix was then analyzed as described by (Lun et al., 2016b). Low quality cells were defined from several quality control metrics, including the log-library sizes and log-number of expressed features (where small outlier values were removed), and the proportion of reads mapped to the ERCC spike-ins or mitochondrial genome (where large outliers were removed). This led to the inclusion of 65 cells for the downstream analysis. Outlier values were defined as three median absolute deviations above or below the median. Low-abundance genes were defined as those with average counts below 1 and were filtered out, leaving 19,650 for downstream analysis. Normalization was performed for endogenous genes by computing size factors with the deconvolution method (Lun et al., 2016a). For spike-in transcripts, size factors were computed from the sum of spike-in counts in each cell. In both cases, the size factors were used to obtain normalized log-expression values for all genes/transcripts in all cells. To identify highly variable genes (HVGs), a trend was fitted to the variance of the log-expression values against the mean for the spike-ins. This was used to decompose the variance for endogenous genes into biological and technical components. HVGs were defined as those genes where the total variance was significantly higher than the technical trend (FDR<0.05) and the biological component was at least 0.5. The log-expression values for the HVGs across all cells were then used in a principal components analysis. Genes correlated with PC1 were identified using limma (Ritchie et al., 2015) as previously described. The observation that PC1 corresponds to cell maturation arose from unbiased analysis and was not fitted to

match some criteria. Accession numbers E-MTAB-6058 can be used to access fastq files single cell RNA seq samples on ArrayExpress EMBL-EBI.

CD marker bioinformatics

The cell surface marker protein query database contains predicted protein features based on human protein sequences from release 2013_04 of the UniProtKB/Swiss-Prot database, which can be used to identify putative cell surface markers within a list of genes. To create the database, proteins with sequence length > 1,500 amino acids were excluded as they were not compatible with some of the programs used for the feature predictions. A representative set of proteins with less than 90% pair-wise sequence identity was subsequently identified using the CD-HIT program (Li and Godzik, 2006). This resulted in a set of 19281 proteins, for which predictions were obtained using the following programs: MEMSAT-SVM (transmembrane segment prediction (Nugent and Jones, 2009), PredGPI (GPI anchor prediction, (Pierleoni et al., 2008), the specific Support Vector Machine trained for GO term GO:0005886 'plasma membrane' within FFPred2 (version 2.0, (Minneci et al., 2013), and DISOPRED2 (protein disorder prediction,(Ward et al., 2004). The predictions were used to label all proteins in the database as either "positive" when it was predicted to be a putative cell surface marker, or "negative" otherwise. Criteria for assigning a putative cell surface marker required two criteria: i) at least 16 transmembrane helices predicted by MEMSAT-SVM *or* a PredGPI prediction of "Highly Probable", and ii) a "plasma membrane" FFPred2 prediction with confidence ≥ 0.8. Applying these rules to the 19281 proteins present in the database, 3367 were labelled as "positive" and 15914 as "negative". A benchmarking test was performed against a list of UniProt Identifiers for 206 known human cluster of differentiation cell surface (CD) markers to evaluate the performance of the prediction pipeline, which showed 85% sensitivity (168/206 marked as positive).

SUPPLEMENTAL REFERENCES

CHEN, E. Y., TAN, C. M., KOU, Y., DUAN, Q., WANG, Z., MEIRELLES, G. V., CLARK, N. R. & MA'AYAN, A. 2013. Enrichr: interactive and collaborative HTML5 gene list enrichment analysis tool. *BMC Bioinformatics,* 14**,** 128.

GONZALEZ-CORDERO, A., KRUCZEK, K., NAEEM, A., FERNANDO, M., KLOC, M., RIBEIRO, J., GOH, D., DURAN, Y., BLACKFORD, S. J. I., ABELLEIRA-HERVAS, L., SAMPSON, R. D., SHUM, I. O., BRANCH, M. J., GARDNER, P. J., SOWDEN, J. C., BAINBRIDGE, J. W. B., SMITH, A. J., WEST, E. L., PEARSON, R. A. & ALI, R. R. 2017. Recapitulation of Human Retinal Development from Human Pluripotent Stem Cells Generates Transplantable Populations of Cone Photoreceptors. *Stem Cell Reports*.

KULESHOV, M. V., JONES, M. R., ROUILLARD, A. D., FERNANDEZ, N. F., DUAN, Q., WANG, Z., KOPLEV, S., JENKINS, S. L., JAGODNIK, K. M., LACHMANN, A., MCDERMOTT, M. G.,

MONTEIRO, C. D., GUNDERSEN, G. W. & MA'AYAN, A. 2016. Enrichr: a comprehensive gene set enrichment analysis web server 2016 update. *Nucleic Acids Res,* 44**,** W90-7.

LI, W. & GODZIK, A. 2006. Cd-hit: a fast program for clustering and comparing large sets of protein or nucleotide sequences. *Bioinformatics,* 22**,** 1658-9.

LIAO, Y., SMYTH, G. K. & SHI, W. 2014. featureCounts: an efficient general purpose program for assigning sequence reads to genomic features. *Bioinformatics,* 30**,** 923-30.

LOVE, M. I., HUBER, W. & ANDERS, S. 2014. Moderated estimation of fold change and dispersion for RNA-seq data with DESeq2. *Genome Biol,* 15**,** 550.

LUN, A. T., BACH, K. & MARIONI, J. C. 2016a. Pooling across cells to normalize single-cell RNA sequencing data with many zero counts. *Genome Biol,* 17**,** 75.

LUN, A. T., MCCARTHY, D. J. & MARIONI, J. C. 2016b. A step-by-step workflow for low-level analysis of single-cell RNA-seq data with Bioconductor. *F1000Res,* 5**,** 2122.

MINNECI, F., PIOVESAN, D., COZZETTO, D. & JONES, D. T. 2013. FFPred 2.0: improved homology-independent prediction of gene ontology terms for eukaryotic protein sequences. *PLoS One,* 8**,** e63754.

NUGENT, T. & JONES, D. T. 2009. Transmembrane protein topology prediction using support vector machines. *BMC Bioinformatics,* 10**,** 159.

PIERLEONI, A., MARTELLI, P. L. & CASADIO, R. 2008. PredGPI: a GPI-anchor predictor. *BMC Bioinformatics,* 9**,** 392.

RITCHIE, M. E., PHIPSON, B., WU, D., HU, Y., LAW, C. W., SHI, W. & SMYTH, G. K. 2015. limma powers differential expression analyses for RNA-sequencing and microarray studies. *Nucleic Acids Res,* 43**,** e47.

SUPEK, F., BOSNJAK, M., SKUNCA, N. & SMUC, T. 2011. REVIGO summarizes and visualizes long lists of gene ontology terms. *PLoS One,* 6**,** e21800.

WANG, Y., MACKE, J. P., MERBS, S. L., ZACK, D. J., KLAUNBERG, B., BENNETT, J., GEARHART, J. & NATHANS, J. 1992. A locus control region adjacent to the human red and green visual pigment genes. *Neuron,* 9**,** 429-40.

WARD, J. J., SODHI, J. S., MCGUFFIN, L. J., BUXTON, B. F. & JONES, D. T. 2004. Prediction and functional analysis of native disorder in proteins from the three kingdoms of life. *J Mol Biol,* 337**,** 635-45.
